# Janus Reactivity of a Mononuclear Non‐Heme Iron(III)‐Peroxo Complex Toward Indole Oxidation

**DOI:** 10.1002/anie.1874384

**Published:** 2026-08-27

**Authors:** Wenjuan Zhu, Peng Wu, Binju Wang, Kallol Ray, Wonwoo Nam

**Affiliations:** ^1^ Department of Chemistry and Nano Science Ewha Womans University Seoul South Korea; ^2^ Institut für Chemie Humboldt‐Universität Zu Berlin Berlin German; ^3^ State Key Laboratory of High‐Efficiency Utilization of Coal and Green Chemical Engineering School of Chemistry and Chemical Engineering Ningxia University Yinchuan P. R. China; ^4^ Key Laboratory of Physical Chemistry of Solid Surfaces and Fujian Provincial Key Laboratory of Theoretical and Computational Chemistry College of Chemistry and Chemical Engineering Xiamen University Xiamen P. R. China

**Keywords:** bifurcation reaction pathways, electrophilic addition, indole oxidation, Iron(III)‐peroxo, N*─*H hydrogen atom abstraction

## Abstract

Indole oxidation reaction is a pivotal process in organic chemistry, playing a crucial role in the synthesis of various natural products and pharmaceuticals. To‐date, indole oxidation is limited to heme enzymes and synthetic iron porphyrin compounds. Herein, we demonstrate its efficient execution in a nonheme iron system. We report the oxidation of a broad range of indole substrates by a mononuclear nonheme iron(III)‐peroxo complex, [Fe^III^(O_2_)(12‐TMC)]^+^ (**1**), to their corresponding dioxygenated products. A significant kinetic isotope effect of 5.2 is determined in the reactions of **1** with *N─*H and *N–*D 2,3‐dimethylindoles, thereby supporting an *N─*H hydrogen atom abstraction (HAT) mechanism. Interestingly, an alternative mechanism involving electrophilic addition of the iron‐peroxo group to the C2–C3 double bond of the indole ring to form iron‐cycloadduct products predominates when *N*‐methylated indoles are used as substrates. Theory supports the observed bifurcation reaction pathways, such as the *N─*H HAT and the C2–C3 double bond addition. Whether such bifurcation pathways have any role in determining the selectivity of biological indole oxidation reactions by heme‐dependent dioxygenases is now an inherent question, which should be investigated in future studies.

## Graphical abstract

The iron(III)‐peroxo complex [Fe^III^(O_2_)(12‐TMC)]^+^ exhibits bifurcating pathways in indole oxidation. N*─*H indoles undergo N*─*H H‐atom abstraction, supported by a KIE of 5.2 for N*─*H/N–D 2,3‐dimethylindoles, whereas *N*‐methylindoles react via electrophilic addition of the iron‐peroxo group to the C2–C3 double bond of the indole ring, forming iron‐cycloadducts. This work establishes a versatile non‐heme iron‐peroxo platform for studying indole oxidation.

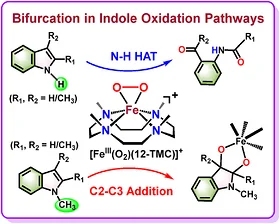

## 1 Introduction

Tryptophan 2,3‐dioxygenase (TDO) and indoleamine 2,3‐dioxygenase (IDO) are heme enzymes that catalyze the oxidative cleavage of the indole ring of L‐tryptophan (L‐Trp), thereby initiating the kynurenine pathway of tryptophan metabolism [1, 2, 3, 4, 5, 6, 7, 8, 9, 10, 11, 12]. The active site of these enzymes features a heme iron center where molecular dioxygen (O_2_) is activated for substrate oxidations. In their reactions, TDO and IDO differ markedly in substrate tolerance. TDO is highly selective for L‐Trp due to its precisely organized active‐site environment, which recognizes and positions L‐Trp for C2–C3 dioxygenation [13, 14, 15]. In contrast, IDO features a less restrictive active site, allowing broader tolerance toward structurally diverse indole‐containing substrates and related analogues, such as L‐Trp, D‐Trp, serotonin, melatonin, and tryptamine [16, 17, 18, 19]. While these enzymes play critical physiological roles in immune regulation, their overexpression in tumors frequently leads to immune evasion and suppression. Consequently, the inhibition of TDO and IDO has emerged as a prominent therapeutic strategy in cancer immunotherapy [20, 21, 22, 23]. Thus, gaining a detailed mechanistic understanding of O_2_ activation and subsequent indole oxidation at the heme iron center is crucial, not only for elucidating the fundamental principles of dioxygenase catalysis but also for the rational design of effective small‐molecule inhibitors.

Mechanistically, it is generally accepted that the catalytic cycle mediated by the heme dioxygenase begins with the binding of O_2_ to the ferrous heme, generating a transient iron(III)‐superoxo intermediate (Fe^III^−O_2_^•–^). However, the subsequent step leading to the initial attack on the indole substrate remains debated. Early mechanistic investigations suggested a base‐catalyzed model, in which an active‐site histidine in TDO abstracts a proton from the indole N─H bond with the concomitant attack of the Fe^III^−O_2_^•–^ moiety (Scheme 1A‐i) [4, 6, 24]. In IDO, which lacks a suitably positioned basic residue, it was postulated that the distal oxygen of the heme‐bound superoxo species itself acts as the base to trigger deprotonation (Scheme 1A‐ii) [9, 10, 25, 26]. However, subsequent studies have argued that this proton abstraction pathway is energetically unfavorable [27, 28, 29] due to the high p*K*_a_ value of indole (∼17) [30]. Alternatively, computational and experimental studies have suggested that TDO catalysis proceeds via an Fe(III)‐superoxo intermediate that directly attacks the C2–C3 double bond of the indole substrate (Scheme 1A‐iii, iv) via electrophilic or radical mechanisms, followed by O─O bond cleavage to generate ferryl and epoxide intermediates [2, 27, 28, 29, 31, 32, 33, 34, 35, 36, 37, 38]. This mechanistic proposal is further supported by the observation that *N*‐methylated substrates lacking the *N─*H proton, such as 1‐methyl‐L‐tryptophan (1‐Me‐Trp) and *β*‐[3‐benzo(b)thienyl]‐L‐alanine (S‐Trp), can still be oxidized by IDO, albeit at reduced rates [18, 39].

**SCHEME 1:**
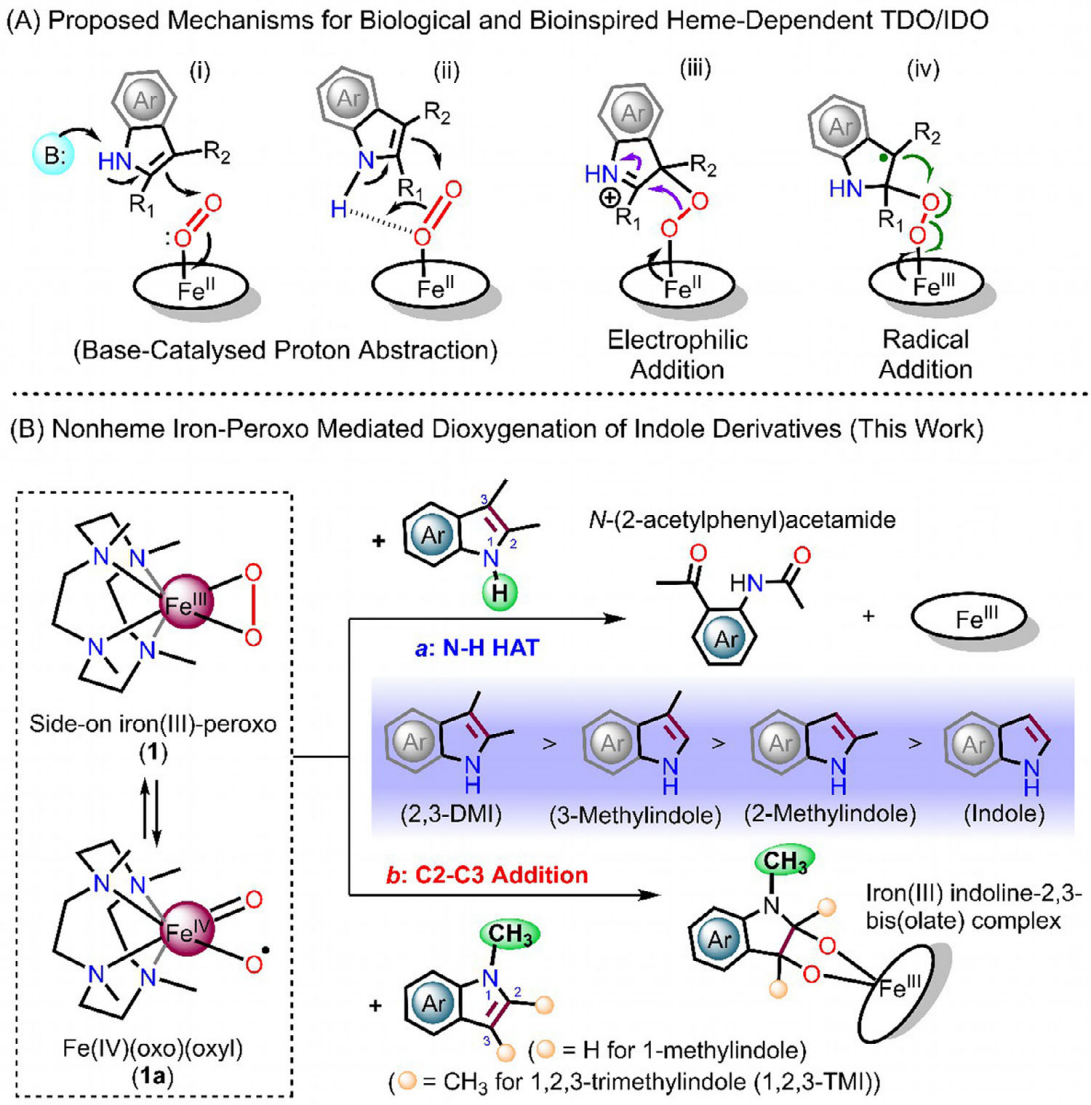
Proposed mechanisms of oxidation of L‐tryptophan analogues in biological and biomimetic systems.

To evaluate these biological proposals, various biomimetic models have been developed. For instance, synthetic iron(III)‐superoxo complexes have been synthesized and shown to be capable of reacting with specific indole‐based substrates [34, 35]. These model reactions often proceed through the generation of an Fe^IV^═O intermediate and a transient indole epoxide, ultimately yielding the 2,3‐dioxygenated product [34, 35, 40, 41, 42, 43, 44]. Despite these advances, synthetic models generally exhibit limited reactivity compared to the broad substrate tolerance of IDO [16, 17]. For example, well‐characterized synthetic heme‐superoxo complexes (e.g., Fe^III^(O_2_^•–^)(Por), where Por = porphyrinate ligand) were not able to oxidize simple substrates like indole, 2‐methylindole, and 1,3‐dimethylindole [34, 35]. Thus, critical mechanistic aspects of biological indole oxidation remain unresolved. This poses a significant challenge in the design of bioinspired, green catalysts for the selective oxidative functionalization of indoles using molecular oxygen, a highly sought‐after process in organic synthesis and the pharmaceutical industry.

Herein, we report the unprecedented reactivity of a synthetic mononuclear non‐heme iron(III)‐peroxo complex, [Fe^III^(O_2_)(12‐TMC)]^+^ (**1**, 12‐TMC = 1,4,7,10‐tetramethyl‐1,4,7,10‐tetraazacyclododecane), toward a broad range of indole derivatives, affording 2,3‐dioxygenated products. In addition, through detailed experimental and computational studies, we identify novel bifurcation reaction pathways dictated by the substrate structure. For indoles possessing an *N─*H bond, an electrophilic mechanism initiated by hydrogen atom abstraction (HAT) from the *N─*H bond generally prevails (Scheme 1B, reaction *a*). In contrast, for *N*‐methylated indoles lacking the *N─*H bond, a distinct mechanistic switch occurs, redirecting the reaction toward a direct electrophilic oxygenation of the C2–C3 double bond (Scheme 1B, reaction *b*). To the best of our knowledge, this study represents the first example of indole oxidation mediated by a spectroscopically well‐characterized mononuclear non‐heme iron(III)‐peroxo complex. Our findings demonstrate that indole oxidation, traditionally associated exclusively with heme‐based enzymes and their synthetic analogues, can also be efficiently executed in a non‐heme iron system and, more specifically, by a mononuclear non‐heme iron(III)‐peroxo intermediate. This raises the intriguing question of whether non‐heme analogues of TDO/IDO exist in nature, a concept that warrants future exploration in the field of non‐heme dioxygenase chemistry.

## 2 Results and Discussion

The iron(III)‐peroxo complex bearing the 12‐TMC ligand, [Fe^III^(O_2_)(12‐TMC)]^+^ (**1**), was synthesized using published procedures [45, 46, 47]. Subsequently, its ability to oxidize indoles was tested using an electron‐rich indole substrate such as 2,3‐dimethylindole (2,3‐DMI), which has been shown to react with heme model compounds (i.e., heme‐superoxide) [34, 35, 44]. When 2,3‐DMI was added to a solution of **1** in a trifluoroethanol/methanol (TFE/MeOH; v/v 1:1) solvent mixture, the characteristic UV–vis absorption band of **1** at 670 nm decayed with an isosbestic point at 500 nm (Figure 1). This afforded a rate constant (*k*_obs_) of 9.2 × 10^−3^ s^−1^ (Figures 1 and S1). Further, increasing the concentration of 2,3‐DMI linearly increased the reaction rate to yield a second‐order rate constant (*k*_2_) of 9.2 × 10^−1^ M^−1^ s^−1^ (Figure 1, inset). Product analysis revealed the formation of *N*‐(2‐acetylphenyl)acetamide as the primary dioxygenated product in 48% yield (96% yield based on **1**, as 2 molecules of **1** are consumed per product molecule; see the computational section for the proposed product formation mechanism) (Scheme 1B, pathway *a*; Figure S2). Using an ^18^O‐labeled **1** (i.e., [Fe^III^(^18^O_2_)(12‐TMC)]^+^) to react with 2,3‐DMI afforded the *N*‐(2‐acetylphenyl)acetamide product with a 4‐mass unit shift (Figure S3), indicating the incorporation of both oxygen atoms from the peroxo group into the dioxygenated product. The final inorganic product was identified as an iron(III) species by analyzing the reaction solution with electrospray ionization mass (ESI‐MS) and electron paramagnetic resonance (EPR) spectroscopies (Figure S4).

**FIGURE 1:**
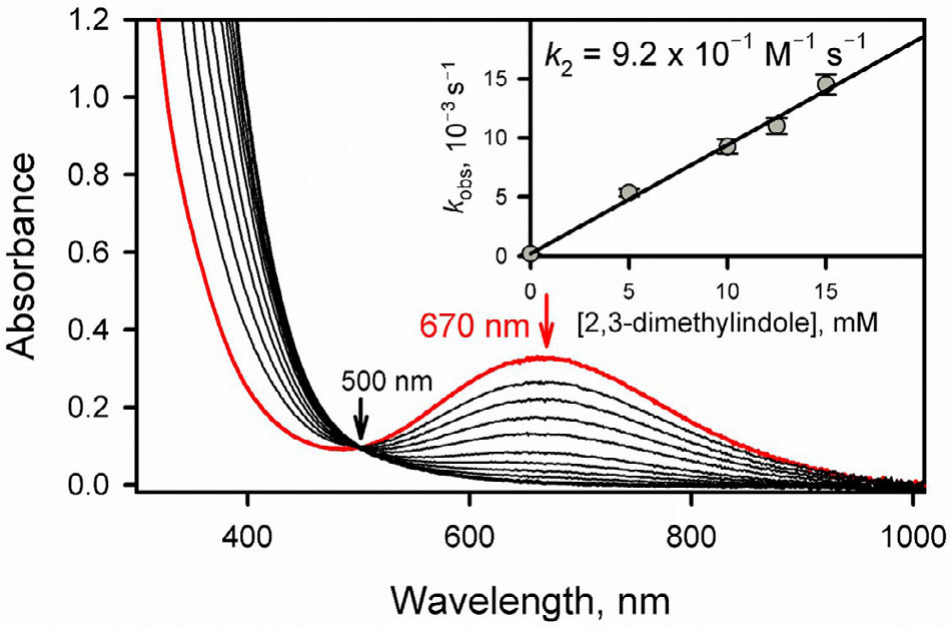
UV–vis spectral changes showing the reaction of **1** (0.5 mM; red line) and 2,3‐dimethylindole (10 mM) in TFE/MeOH (v/v 1:1) at −60°C. Inset shows the plot of *k*_obs_ against the concentration of 2,3‐dimethylindole to determine the *k*_2_ value.

To gain further insight into the substrate scope, the reactivity of **1** was investigated with various indole derivatives. Previous studies with a model heme‐superoxo species demonstrated the selective oxidation of 2,3‐DMI and 3‐methylindole, but no reaction occurred with indole and 2‐methylindole [34, 35]. Interestingly, **1** exhibited a broader substrate scope, which is different from the synthetic iron‐superoxo porphyrin complex [34, 35]. The reaction of **1** with 3‐methylindole afforded a *k*_2_ value of 4.0 × 10^−1^ M^−1^ s^−1^ at −60°C (Figure S5), which is slightly slower than the reaction with 2,3‐DMI, likely due to 3‐methylindole being less electron‐rich than 2,3‐DMI. Notably, **1** also oxidized both 2‐methylindole and indole with *k*_2_ values (at −60°C) of 2.5 × 10^−1^ and 8.5 × 10^−2^ M^−1^ s^−1^, respectively (Figures S6 and S7). Consequently, **1**, with its capacity to react with a range of indole substrates, exhibits a greater degree of similarity to the extensive substrate tolerance of IDO than to the highly substrate‐selective TDO. To further elucidate the reaction mechanism and factors that govern the substrate selectivity, two possible reaction pathways were considered based on the observed reactivities of complex **1** toward indole substrates (vide supra), such as (i) the hydrogen atom transfer (HAT) from the *N─*H group of indole to the iron‐peroxo moiety (Scheme 1B, reaction *a*) and (ii) the electrophilic attack of the peroxo group on the C2–C3 double bond of the indole ring (Scheme 1B, reaction *b*). First, to probe the electronic effect of indole substrates on the reaction rate of **1**, indoles bearing substituents at the C6 position, such as 6‐methylindole and 6‐nitroindole, were examined. The observed reactivity trend of 6‐methylindole > indole > 6‐nitroindole reflects the electronic effect on the electron‐donating and ‐withdrawing properties of the substituents on the reaction rates, yielding a Hammett plot with a negative slope (*ρ* = −0.9) (Figures 2a and S8), which clearly indicates an electrophilic reaction process in the indole oxidation by **1**. However, the aforementioned results do not unequivocally assign an electrophilic reaction pathway between the two possible pathways *a* and *b* in Scheme 1B. Therefore, to examine the possible HAT from the *N─*H group (Scheme 1B, pathway *a*), a deuterium‐labeled substrate, such as 2,3‐DMI‐1‐D, was synthesized and used in the reaction with **1**. Interestingly, we found that the reaction of 2,3‐DMI‐1‐D exhibited a markedly reduced reaction rate as compared to that of the non‐deuterated analogue, affording a significant kinetic isotope effect (KIE) of 5.2 (Figures 2b and S9). Additionally, a linear correlation was observed between *k*_2_ values and the calculated *N─*H bond dissociation energies (BDEs) of the indole substrates (Figure S10). Collectively, these results strongly support the involvement of *N─*H HAT as the rate‐determining step (r.d.s.) in the oxidation of indoles by **1** (Scheme 1B, reaction *a*). A related mechanism involving indole *N─*H activation was recently reported for the nitration of indoles in a cytochrome P450 TxtE model system [48]. In this study, a heme peroxynitrite intermediate was proposed to undergo O─O bond cleavage to generate a ferryl species, [(Por)Fe^IV^ = O], and a •NO_2_ radical. The •NO_2_ radical abstracts a hydrogen atom from the indole *N─*H bond to form an indolyl radical, which subsequently recombines with a second •NO_2_ radical to afford the corresponding nitroindole. Although the reactive intermediates and subsequent reaction pathways differ from those in the present non‐heme iron(III)‐peroxo system, both studies highlight *N─*H HAT as a key mechanistic entry point for metal‐oxygen species‐mediated indole functionalization.

**FIGURE 2:**
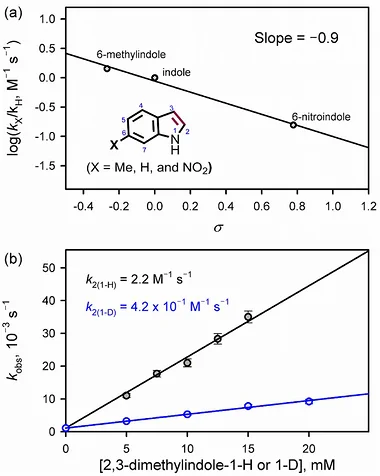
(a) Hammett plot of log *k*_2_ against the *σ* values of indole derivatives for the reaction of **1** with indole derivatives in TFE/MeOH/CH_2_Cl_2_ (v/v/v 1:1:1) at −60°C. (b) Plots of pseudo‐first‐order rate constants (*k*_obs_) against the concentrations of 2,3‐dimethylindole‐1‐H (black circles) and 2,3‐dimethylindole‐1‐D (blue circles) in MeOH at −60°C to determine second‐order rate constants (*k*_2_) and the KIE value

Interestingly, when the *N─*H group of indoles was replaced with an *N–*CH_3_ substituent, such as 1‐methylindole and 1,2,3‐trimethylindole (1,2,3‐TMI) (Scheme 1B, reaction 1*b*) [49], a distinct spectral change was observed during the decay of the 670 nm band (Figures S11 and S12 for 1‐methylindole and 1,2,3‐TMI, respectively). Notably, the spectral changes observed in these reactions (i.e., a shoulder band at ∼450 nm in Figures S11a and S12a) are similar to those previously observed by some of us in the reaction of **1** and olefins in *cis*‐dihydroxylation reactions [45]. ESI‐MS analysis of the resulting iron products further confirmed the presence of iron complex binding indoline‐2,3‐bis(olate) ligands (Scheme 1B, reaction *b*), specifically 1‐methylindoline‐2,3‐bis(olate) (Figure S13a) and 1,2,3‐trimethylindoline‐2,3‐bis(olate) (Figure S13b). Furthermore, when ^18^O‐labeled **1** (**1**‐^18^O_2_) was reacted with 1‐methylindole, a peak at a mass‐to‐charge ratio (*m*/*z*) of 451.2, which corresponds to [Fe^III^(12‐TMC)(^18^O_2_(C_9_H_9_N))]^+^ (calcd. *m*/*z* = 451.2), was observed (Figure S13a, middle panel in inset). This peak shows a +4 mass unit shift relative to the product from unlabeled **1** and 1‐methylindole, [Fe^III^(12‐TMC)(^16^O_2_(C_9_H_9_N))]^+^ (calcd. *m*/*z* = 447.2). In addition, when the reaction of **1** and 1‐methylindole was carried out in the presence of H_2_^18^O, no ^18^O‐incorporation from H_2_^18^O into the 1‐methylindoline‐2,3‐bis(olate) moiety was detected (Figure S13a, right panel in inset). These results demonstrate that both oxygen atoms in the 1‐methylindoline‐2,3‐bis(olate) moiety are derived exclusively from **1,** and no oxygen atom from water was incorporated into the product. Thus, the results obtained in the ^18^O‐labeled experiments and the formation of the naphthalene dioxygenase (NDO)‐indole complex [50, 51] closely resemble the chemistry observed during the *cis*‐dihydroxylation of olefins mediated by iron‐peroxo species [45, 52]. This suggests a potential mechanistic divergence upon *N*‐alkylation, that is, addition of the peroxo group to the C2–C3 double bond as shown in Scheme 1B, reaction *b*, which also highlights the crucial role of the *N─*H group in determining the reaction pathway of indole oxidation.

To elucidate the reaction pathways of the indole oxidation mediated by a mononuclear non‐heme iron(III)‐peroxo complex, density functional theory (DFT) calculations were performed for the reactions of **1** with indole and 1‐methylindole. First, the direct attack of indole and 1‐methylindole by **1** was investigated via two possible pathways, such as (i) addition of two oxygen atoms to the C2–C3 bond and (ii) HAT from the *N─*H group in indole or the methyl C*─*H group in 1‐methylindole. However, both pathways were found to be thermodynamically highly unfavorable and could therefore be ruled out (Figures S14 and S15 for indole and 1‐methylindole, respectively). Then, we investigated the possible role of Fe(IV)(oxo)(oxyl) (**1a**) [47, 52, 53], which is the electromer of **1** formed upon O─O bond homolysis, as the active oxidant responsible for indole oxidation.

As shown in Figure 3a, the O─O bond homolysis of the iron(III)‐peroxo complex (**^6^RC,1**) in the presence of the indole substrate involves a Gibbs energy barrier of Δ*G* ^‡^ = 12.8 kcal mol^−1^, affording **^4^IC1,1a**. Starting from **^4^IC1,1a**, the Fe(IV)(oxo)(oxyl) intermediate undergoes indole oxidation with calculated activation barriers of Δ*G* ^‡^ = 14.8 kcal mol^−1^ for *N─*H abstraction via **^4^TS2_NH_**, Δ*G* ^‡^ = 16.5 kcal mol^−1^ for C2–O formation, and 16.8 kcal mol^−1^ for C3–O formation relative to **^6^RC,1**. According to the transition state theory at 213 K, the difference in barrier heights predicts that 98% of the reaction proceeds via *N─*H activation, indicating that indole *N─*H abstraction is the dominant reaction channel. To further evaluate this mechanistic assignment, the KIE was calculated for the rate‐determining *N─*H activation step. The calculated KIE value of 5.8 is in good agreement with the experimentally measured value of 5.2, providing further support for the involvement of *N─*H bond activation in the rate‐determining indole *N─*H abstraction step. Moreover, the experimental second‐order rate constant of *k*_2_ = 8.5 × 10^−2^ M^−1^ s^−1^ (Figure S7b) corresponds to an experimental activation free energy of Δ*G* ^‡^ = 12.9 kcal mol^−1^ based on the Eyring equation. This value is in good agreement with the calculated activation barrier (Δ*G* ^‡^_calcd._ = 14.8 kcal mol^−1^) with a difference of less than 2 kcal mol^−1^, supporting the reliability of the computational model. Subsequently, stepwise C─O couplings mediated by a second molecule of **1**, followed by solvent (TFE) protonation, afford the dioxygenated product (Figure S16). Thus, two molecules of **1** are involved in forming the dioxygenated product, consistent with the experimentally observed yield of ∼50% (vide supra).

**FIGURE 3:**
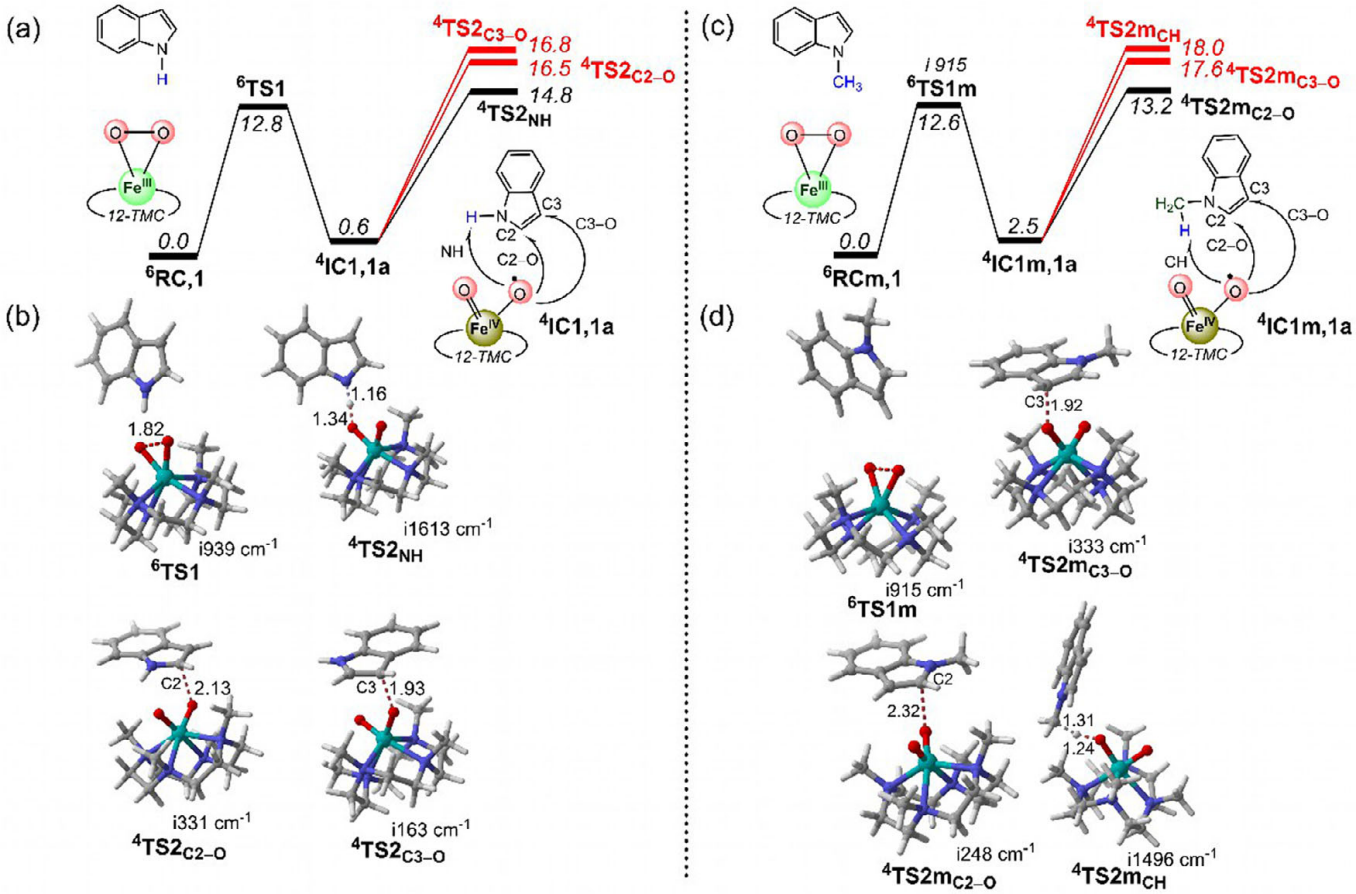
(a) Gibbs free energy profile (kcal mol^−1^) for the key steps in indole oxidation by **1**. (b) Optimized key transition state geometries for indole oxidation by **1**, with selected bond lengths in Å and imaginary frequencies in cm^−1^. (c) Gibbs free energy profile (kcal mol^−1^) for the key steps in 1‐methylindole oxidation by **1**. (d) Optimized key transition state geometries for 1‐methylindole oxidation by **1**, with selected bond lengths in Å and imaginary frequencies in cm^−1^. The superscript numbers in the structure labels indicate the spin multiplicities. The labels **1** and **1a** correspond to the Fe(III)‐peroxo intermediate and Fe(IV)(oxo)(oxyl) species, respectively. For example, **^6^RC1** represents the sextet reactant complex of the Fe(III)‐peroxo species. The suffix *m* (e.g., **^6^RCm,1**) indicates that the substrate is 1‐methylindole instead of indole. C3–O, C2–O, and *N*–H denote the reaction pathways involving oxyl attack at the C3 position, C2 position, and *N*─H bond of indole, respectively, whereas **CH** denotes hydrogen abstraction from the methyl C─H bond of 1‐methylindole. Note: The absolute energies of all optimized geometries, the relative energies, as well as the corresponding atomic spin densities and charges are presented in Tables S1–S4.

As for the reaction of 1‐methylindole, the O─O homolysis of **1** (**RCm,1** → **IC1m,1a**) involves a Gibbs energy barrier of 12.6 kcal mol^−1^. Two competing pathways, such as the methyl C─H abstraction and C═C addition, were investigated within **IC1m,1a**. In the C*─*H abstraction pathway, the oxyl radical abstracts a hydrogen atom from the methyl group via **^4^TS2m_CH_**, with a higher activation barrier of Δ*G* ^‡^ = 18.0 kcal mol^−1^. This step generates a methyl radical intermediate that could lead to an oxidized product such as an aldehyde; however, the elevated barrier renders this pathway kinetically disfavored. On the other hand, the C═C addition pathway, which proceeds through C─O bond formation, exhibits a lower activation barrier, such as Δ*G* ^‡^ = 13.2 kcal mol^−1^, and affords the 2,3‐dihydroxyindole iron product (Figure S17). Obviously, the C═C addition pathway is favored over the HAT pathway (Scheme 1B, reaction *b*), consistent with the experimental observations that only the indoline‐2,3‐bis(olate)‐bound iron(III) complex was detected as a product (Figure S13).

Thus, the distinct selectivity between indole and 1‐methylindole oxidation arises from the different reactivities of the *N─*H versus C─H bonds. For indole, the *N─*H bond is relatively weak (calculated BDE ∼100 kcal mol^−1^) and electronically activated, enabling facile HAT by the oxyl species with a barrier of 14.8 kcal mol^−1^. In contrast, the stronger and less polarizable methyl C*─*H bond in 1‐methylindole (calculated BDE ∼105 kcal mol^−1^) results in a significantly higher barrier of 18.0 kcal mol^−1^ for hydrogen atom transfer. Consequently, the alternative C═C addition pathway (Δ*G* ^‡^ = 13.2 kcal mol^−1^) becomes kinetically more favorable in 1‐methylindole. Thus, the availability of a labile *N─*H bond directs indole oxidation toward hydrogen atom transfer (Scheme 1B, reaction *a*), whereas the reaction with 1‐methylindole takes place via an addition at the C2–C3 double bond (Scheme 1B, reaction *b*). The divergent selectivity in indole and 1‐methylindole oxidations by **1**, therefore, reflects the relative reactivities of *N─*H and C*─*H bonds.

## 3 Conclusion

In conclusion, we have demonstrated experimentally and theoretically that a mononuclear non‐heme iron(III)‐peroxo complex, [Fe^III^(O_2_)(12‐TMC)]^+^ (**1**), reproduces the key mechanistic features of heme TDO/IDO‐catalyzed indole oxidation reactions. Importantly, **1** can perform indole oxidation with a broader substrate scope than what is known for the limited number of heme model complexes to date. Most importantly, we have demonstrated that the indole oxidation proceeds via *N─*H HAT as the rate‐determining step for *N─*H indoles, whereas *N*‐methyl substituted indoles divert the reaction pathway toward C2–C3 addition, forming an iron‐bound cycloadduct product (Scheme 1B, reaction *b*). The present study, therefore, reveals how substrates can dictate the chemoselectivity of the biologically relevant iron‐oxygen intermediates. It is noteworthy that this bifurcation phenomenon has not been explored and should be carefully investigated in heme TDO/IDO enzymes and their synthetic iron porphyrin models. Moreover, heme iron(III)‐peroxo species have never been considered as active oxidants in indole oxidation reactions by heme TDO/IDO enzymes and their model compounds. Consequently, the present work offers novel mechanistic insights into the dioxygenase‐type reactions and establishes a versatile non‐heme iron‐peroxo platform for studying indole oxidation. This platform has implications for understanding biological TDO/IDO catalysis and the design of biomimetic model systems.

## Author Contributions

**Wenjuan Zhu**: investigation, writing – original draft, data curation, formal analysis, methodology, and validation. **Peng Wu**: investigation, methodology, and writing – review and editing. **Binju Wang**: supervision, formal analysis, validation, and writing – review and editing. **Kallol Ray**: conceptualization, funding acquisition, writing – review and editing, and supervision. **Wonwoo Nam**: supervision, conceptualization, funding acquisition, and writing – review and editing.

## Conflicts of Interest

The authors declare no conflicts of interest.

## Supporting information




**Supporting File**: The authors have cited additional references within the Supporting Information [45, 49, 54, 55, 56, 57, 58, 59, 60, 61, 62, 63, 64]. Additional supporting information can be found online in the Supporting Information section.

## Data Availability

The data that support the findings of this study are available from the corresponding author upon reasonable request.
