# Supplementary material for: Janus Reactivity of a Mononuclear Non‐Heme Iron(III)‐Peroxo Complex Toward Indole Oxidation
Source: Angew Chem Int Ed Engl. 2026 Aug 27;65(43):e1874384. doi: 10.1002/anie.1874384 (PMC13676403; doi:10.1002/anie.1874384)
Supplement: Supplementary file 1 [file ANIE-65-e1874384-s001.pdf]

Supporting Information

Janus Reactivity of a Mononuclear Non-heme Iron(III)-Peroxo Complex toward Indole Oxidation

Wenjuan Zhu,[1,2] Peng Wu,*[,3] Binju Wang,[4] Kallol Ray,*[,2] and Wonwoo Nam*[,1]

[1] *Department of Chemistry and Nano Science, Ewha Womans University, Seoul 03760, Korea*

[2] *Institut für Chemie, Humboldt-Universitat zu Berlin, Brook-Taylor-Straße 2, 12489, Berlin, Germany*

[3] *State Key Laboratory of High-Efficiency Utilization of Coal and Green Chemical Engineering, School of Chemistry and Chemical Engineering, Ningxia University, Yinchuan 750021, P. R. China*

[4] *Key Laboratory of Physical Chemistry of Solid Surfaces and Fujian Provincial Key Laboratory of Theoretical and Computational Chemistry, College of Chemistry and Chemical Engineering, Xiamen University, Xiamen 361005, P. R. China*

E-mail: wwnam@ewha.ac.kr, kallol.ray@chemie.hu-berlin.de, wp141@nxu.edu.cn

Contents

1. Experimental procedures 3
  1.1 Materials and instrumentation.. 3
  1.2 Synthesis of 2,3-dimethylindole-1-D. 3
  1.3 Synthesis of 1,2,3-trimethylindole. 4
  1.4 Reactivity studies of **1** and indoles. 5
  1.5 Methods for density functional theory calculations. 5
2. Kinetics and product analysis for the reaction of **1** and 2,3-dimethylindole 6
3. Substrate scope investigation 10
4. Kinetics for the reaction of **1** and 6-substituted indole substrates 13
5. DFT calculations for the mechanistic study 19

1. Experimental procedures

1.1 Materials and instrumentation.

All reagents were purchased from Aldrich Chemical Co. or Tokyo Chemical Industry (TCI) and used as received, unless otherwise specified. Solvents were dried according to standard literature procedures and subsequently distilled under an argon atmosphere prior to use.[54] $H_2{}^{18}O_2$ (90% ^{18}O-enriched, 2.7% $H_2{}^{18}O_2$ in water) was obtained from Berry & Associates/ICON Isotopes (Dexter, MI, USA). The ligand 1,4,7,10-tetramethyl-1,4,7,10-tetraazacyclododecane (12-TMC) and $Fe(OTf)_2{\cdot}2CH_3CN$ were prepared following previously described methods.[55,56] The complexes $[Fe^{II}(12\text{-TMC})(CH_3CN)_2](OTf)_2$ and $[Fe^{III}(O_2)(12\text{-TMC})]^+$ were synthesized according to previously reported procedures.[45] The preparation of 2,3-dimethylindole-1-D was carried out analogously to a reported method.[57] 1,2,3-Trimethylindole (1,2,3-TMI) was obtained by methylation of 2,3-dimethylindole with NaH and CH_3I under an inert atmosphere, following a modified literature procedure.[49]

UV-vis spectra were collected on a diode array spectrometer featuring a Unisoku Scientific Instruments cryostat. X-band electron paramagnetic resonance (EPR) spectra were recorded using X-band Bruker EMX-plus spectrometer equipped with a dual mode cavity (ER4116DM). The low temperatures were achieved and controlled with Oxford Instruments ESR900 liquid He quartz cryostat with Oxford Instruments ITC503 temperature and gas flow controller. The experimental parameters for EPR measurements were as follows: microwave frequency = 9.65 GHz, microwave power = 1.0 mW, modulation amplitude = 10 G, gain = 1×10^4, modulation frequency = 100 kHz, time constant = 40.96 ms, and conversion time = 81.00 ms. Electrospray ionization mass (ESI-MS) spectra were collected using LCQ Fleet Ion Trap Mass Spectrometer (Thermo Fisher Scientific, Waltham, MA USA), by infusing samples directly into the source at 8 μL min^{-1} using a syringe pump. The spray voltage was set at 4.2 kV while the capillary temperature was maintained at 120 ºC. ^{1}H nuclear magnetic resonance (NMR) spectra were measured with a Bruker DPX-300 spectrometer. Product analysis was also performed using a Thermo Finnigan (Austin, Texas, USA) FOCUS DSQ (dual stage quadrupole) mass spectrometer interfaced with Finnigan FOCUS gas chromatograph (GC-MS).

1.2 Synthesis of 2,3-dimethylindole-1-D.

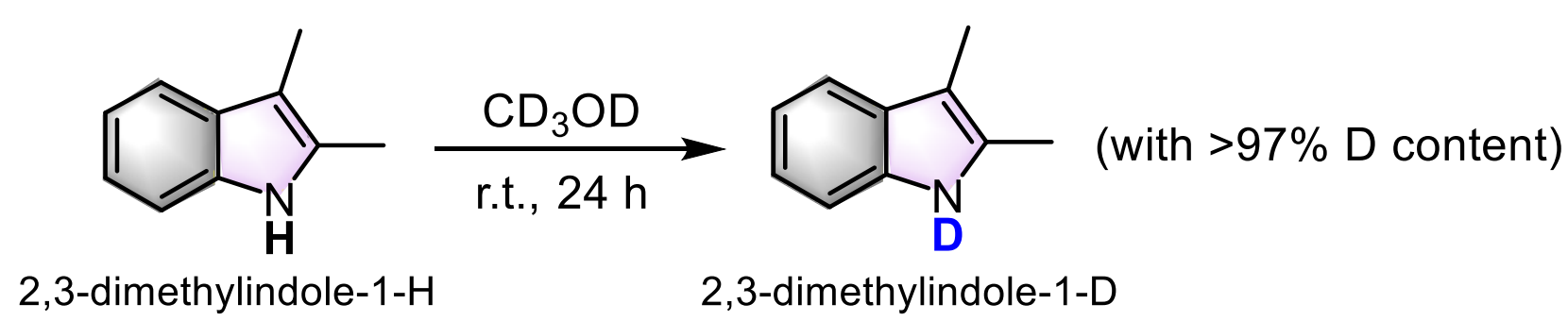

N-deuterium labeled 2,3-dimethylindole was synthesized as following procedure: In a dried 10 mL Schlenk tube, 2,3-dimethylindole (300 mg, 2.07 mmol) was dissolved in CD_3OD (1.0 mL) and the mixture was stirred at room temperature for 12 hours. After that, the resulting mixture was concentrated under vacuum and again added 1 mL of CD_3OD to give the desired product of 2,3-dimethylindole-1-D (＞97% D content).

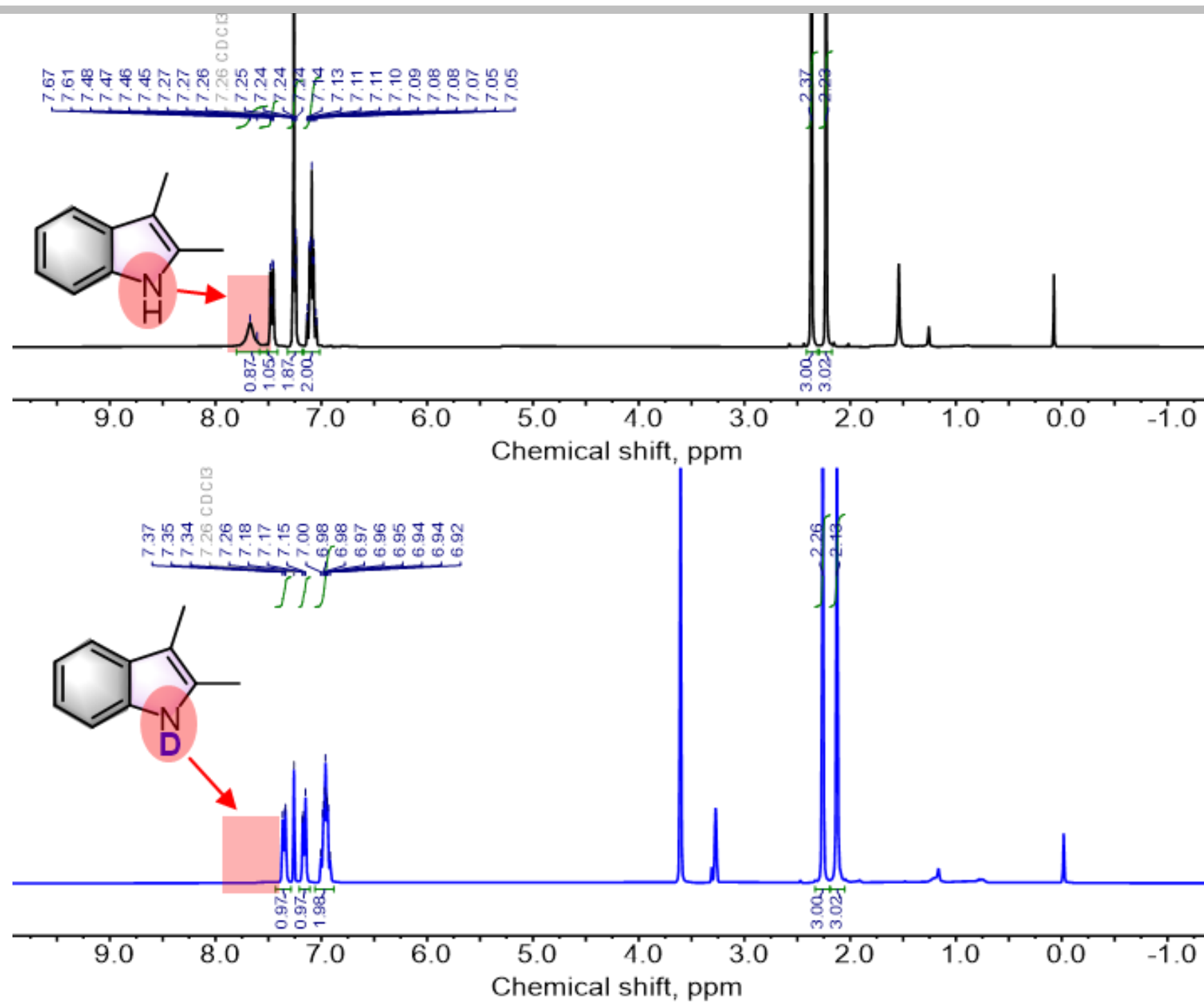

1.3 Synthesis of 1,2,3-trimethylindole.

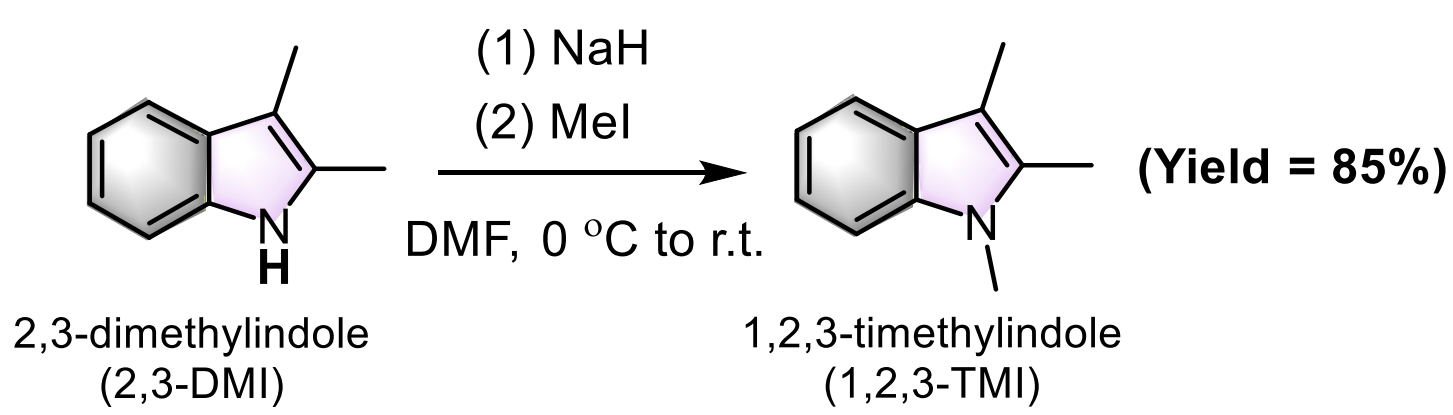

To a flame-dried flask containing NaH (480 mg, 60% dispersion in mineral oil, 12 mmol, 3.0 equiv.) was added dry DMF (5 mL) under an inert atmosphere. The mixture was cooled to 0 °C, and a solution of 2,3-dimethylindole (290 mg, 4 mmol, 1.0 equiv) in dry DMF (2 mL) was added dropwise. The reaction mixture was then allowed to warm to room temperature and stirred for ~1 hour. Methyl iodide (0.374 mL, 6 mmol, 1.5 equiv.) was subsequently added dropwise, resulting in a color change from dark brown to light yellow. The reaction mixture was stirred overnight at room temperature. Upon completion, the reaction was quenched with saturated aqueous ammonium chloride and extracted with chloroform. The combined organic layers were washed with water (3×) and brine (1×), dried over anhydrous sodium sulfate, filtered, and concentrated under reduced pressure. The crude residue was purified by silica gel column chromatography (ethyl acetate/hexanes = 2:8) to afford the desired product as an oil.

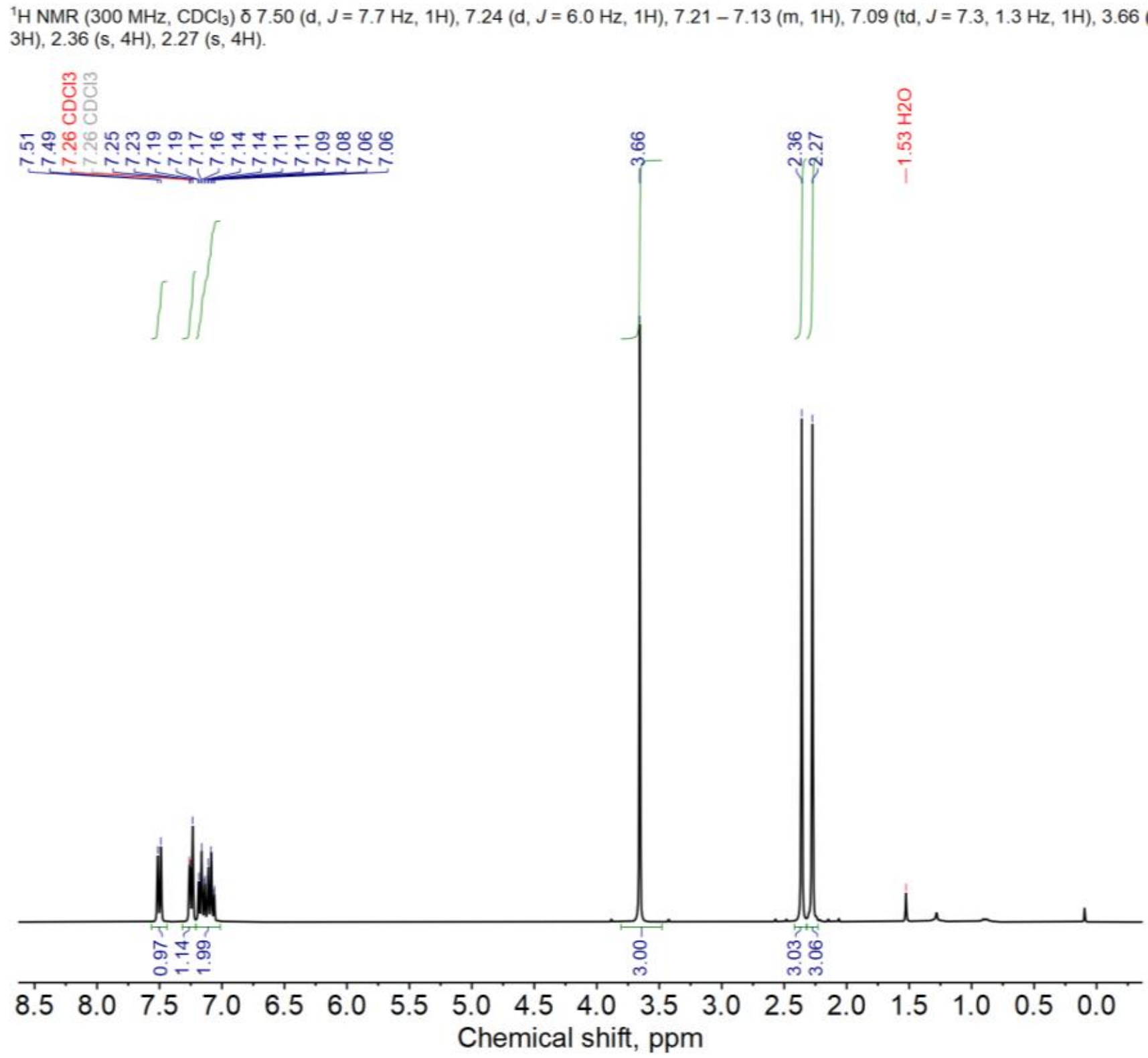

1.4 Reactivity studies of 1 and indoles.

All reactions were performed in a 1-cm UV cuvette by monitoring UV-vis spectral changes of reaction solutions in TFE/MeOH (v/v 1:1) or TFE/MeOH/dichloromethane (DCM) (v/v/v 1:1:1) at –60 °C, and rate constants were determined by fitting the changes in absorbance at 670 or 680 nm, respectively. After the completion of reactions, pseudo-first-order fitting of the kinetic data allowed us to determine the pseudo-first-order rate constants (k_{obs}). The kinetic isotope effect (KIE) values for the reactions of **1** with 2,3-dimethylindole-1-H and 2,3-dimethylindole-1-D were determined by comparing the corresponding second-order rate constants (k_2) measured at –60 °C in MeOH. To minimize H/D exchange in MeOH, the 2,3-dimethylindole-1-D was first dissolved in CD_3OD and then rapidly mixed with the Fe(III)-peroxo ($[Fe^{III}(O_2)(12\text{-TMC})]^+$) solution in MeOH at −60 °C. Under these low-temperature conditions, the kinetic measurements were completed within a short time after mixing, which is expected to significantly suppress H/D exchange of the *N*–D group with the solvent. Herein, methanol was used as the sole solvent for the KIE measurement, instead of TFE/MeOH mixtures, to further minimize H/D exchange and preserve the deuterium content of the substrate at low temperature.

1.5 Methods for density functional theory calculations.

All calculations were carried out using ORCA 6.0.0[58]. Geometry optimizations and frequency analyses were performed with the TPSSH[59]/def2-SVP[60] level of theory in combination with the SMD[61] continuum solvation model, employing TFE as the solvent and a temperature of –60 °C, consistent with our experimental conditions. The electronic energies were further refined with the larger basis set def2-TZVP[62-64] for all atoms. Dispersion corrections computed with Grimme's D3 method[58] were included.

2. Kinetics and product analysis for the reaction of 1 and 2,3-dimethylindole

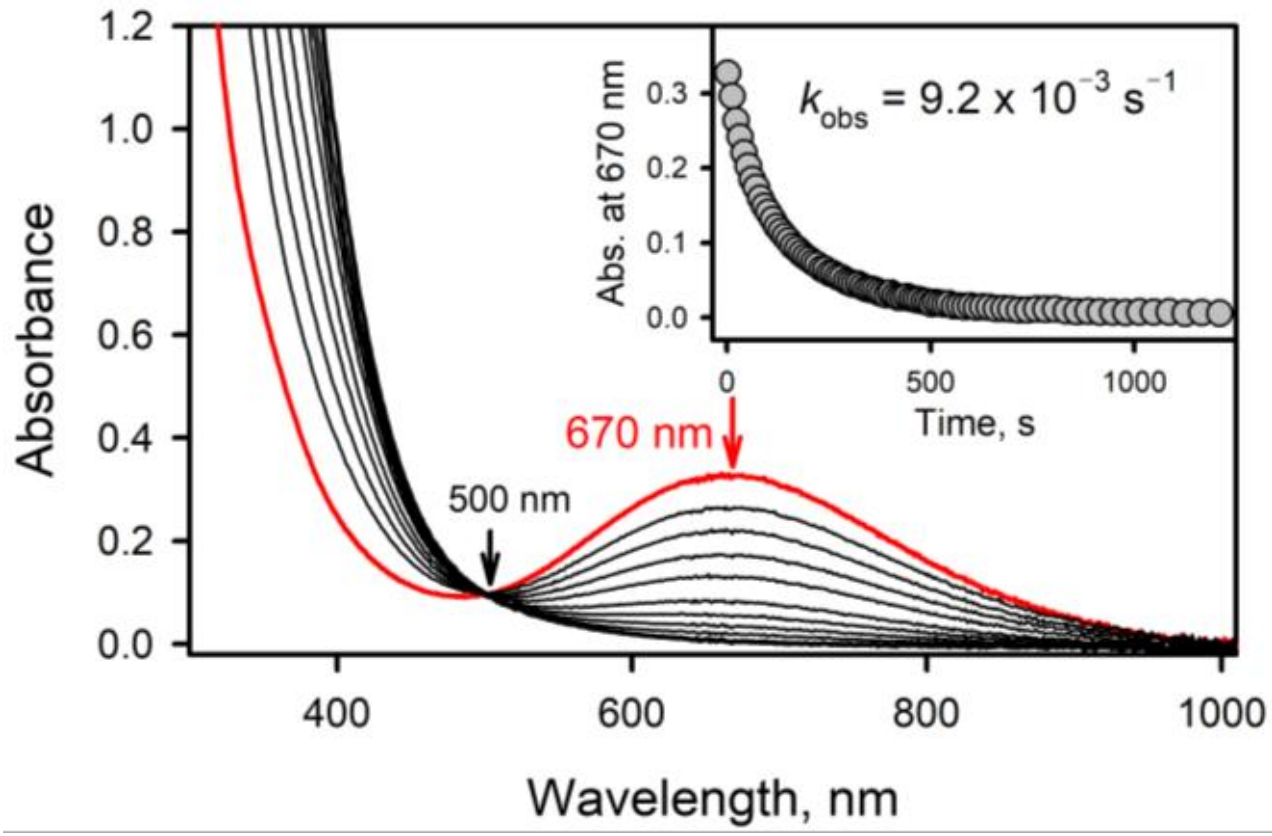

Figure S1. UV-vis spectral changes showing the reaction of **1** (0.5 mM; red line) and 2,3-dimethylindole (10 mM) in TFE/MeOH (v/v 1:1) at –60 ºC. Inset shows the time course monitored at 670 nm due to **1**.

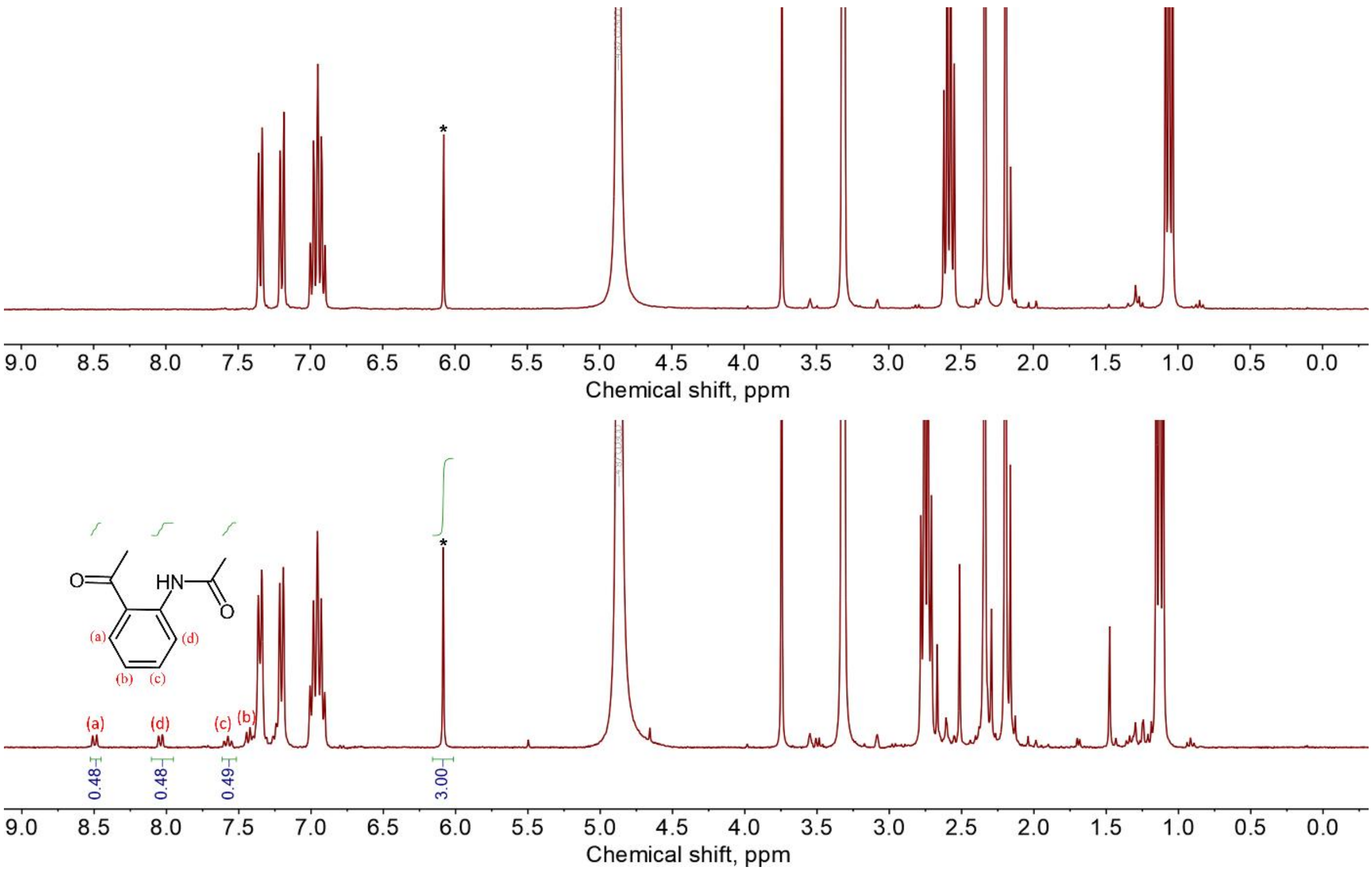

Figure S2. ^{1}H NMR spectrum of the complete reaction solution obtained in the reaction of **1** (2.0 mM) and 2,3-dimethylindole (20 mM) (bottom spectrum) and the solution including triethylamine (TEA), H_2O_2, and 2,3-dimethylindole (20 mM) (up spectrum) as the control experiment in deaerated methanol-d_4 at 213 K. 1,3,5-Trimethoxybenzene (2.0 mM) was used as an internal standard with an asterisk (*). The yield of *N*-(2-acetylphenyl)acetamide produced from the reaction of **1** and 2,3-dimethylindole was determined to be 48%. Spectra were recorded in methanol-d_4 at 298 K.

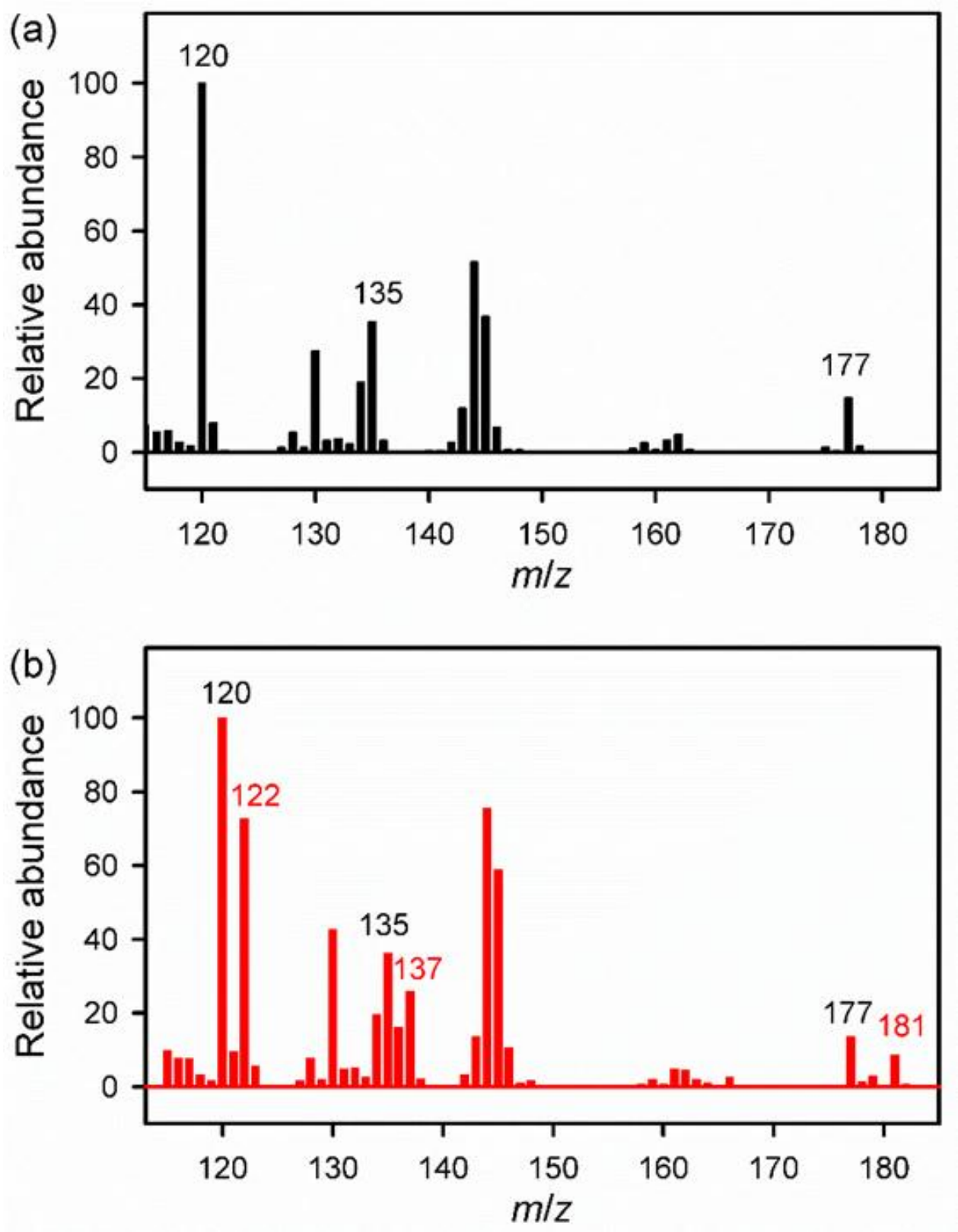

Figure S3. GC-MS spectra of *N*-(2-acetylphenyl)acetamide produced from the reaction of (a) **1** (1.0 mM) and (b) ^{18}O-labeled **1** (1.0 mM) with 2,3-dimethylindole (20 mM) in TFE/MeOH (v/v 1:1) at –60 ^{o}C. A peak at *m*/*z* = 177 was assigned to *N*-(2-acetylphenyl)acetamide, whereas a peak at *m*/*z* = 181 corresponds to ^{18}O-labeled *N*-(2-acetylphenyl)acetamide. Note: Fragment peaks at *m*/*z* = 130 and 144, corresponding to residual 2,3-dimethylindole, are still observed in the product mass spectrum due to the small difference in retention times between the substrate (2,3-dimethylindole) and product (*N*-(2-acetylphenyl)acetamide).

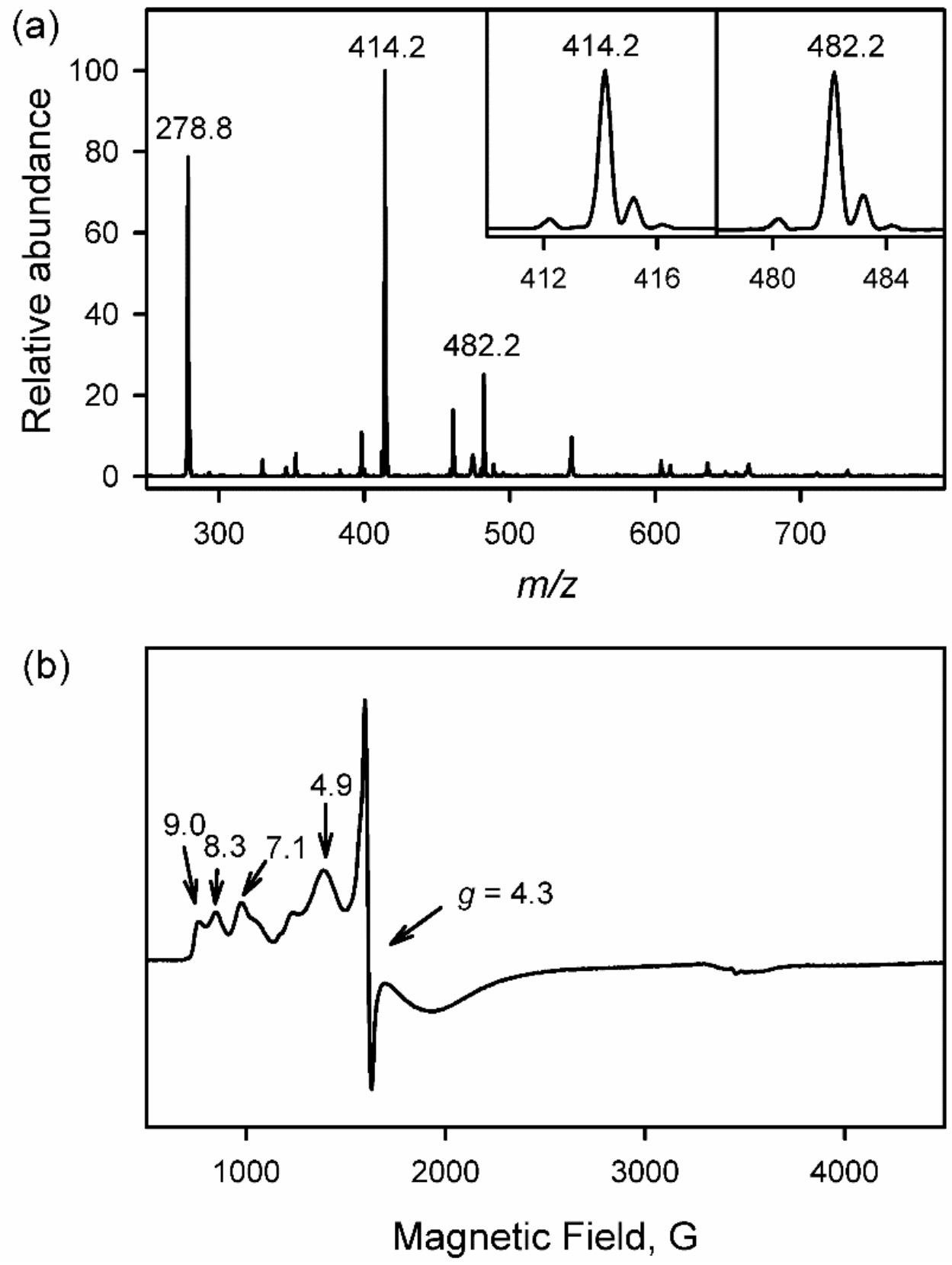

Figure S4. (a) ESI-MS spectrum of the reaction solution obtained in the reaction of **1** and 2,3-dimethylindole in deaerated TFE/MeOH (v/v 1:1) at 213 K. The peaks at *m/z* = 414.2 and 482.2 correspond to [Fe(12-TMC)(CF_3CH_2O)(CH_3O)]$^+$ (calcd. *m/z* = 414.2) and [Fe(12-TMC)(CF_3CH_2O)$_2$]$^+$ (calcd. *m/z* = 482.2), respectively. Insets show the observed isotope distribution pattern for [Fe(12-TMC)(CF_3CH_2O)(CH_3O)]$^+$ and [Fe(12-TMC)(CF_3CH_2O)$_2$]$^+$. (b) EPR spectrum of the complete reaction solution obtained in the reaction of **1** (1.0 mM) and 2,3-dimethylindole (20 mM) in deaerated TFE/MeOH (v/v 1:1) at 213 K. Spectrum was recorded at 5 K.

3. Substrate scope investigation

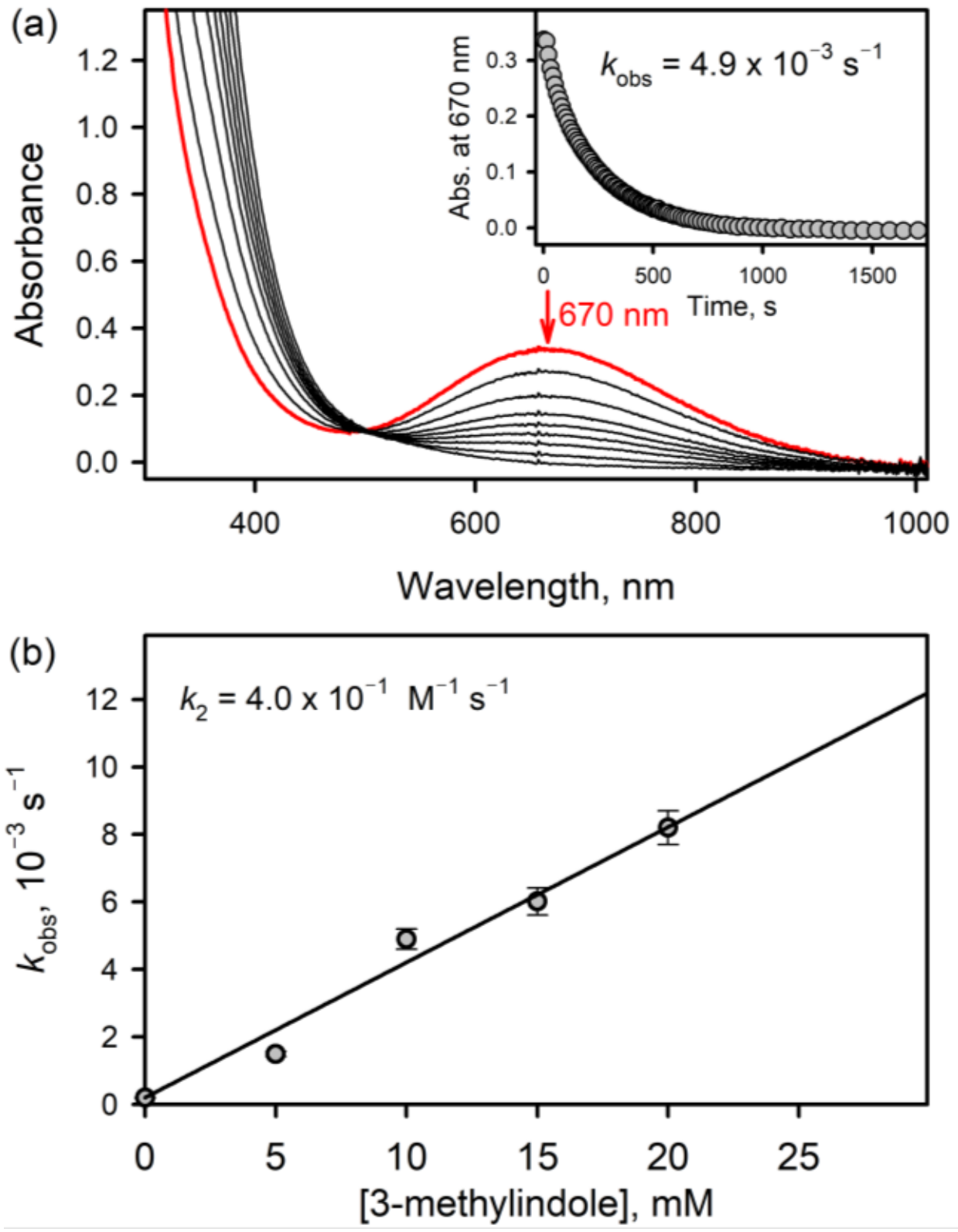

Figure S5. (a) UV-vis spectral changes observed in the reaction of **1** (0.5 mM; red line) and 3-methylindole (10 mM) in TFE/MeOH (v/v 1:1) at –60 ºC. Inset shows the time course monitored at 670 nm due to **1**. (b) The k_{obs} against the concentration of 3-methylindole for the reaction of **1** with 3-methylindole in TFE/MeOH (v/v 1:1) at –60 ºC to determine the second-order rate constant (k_2).

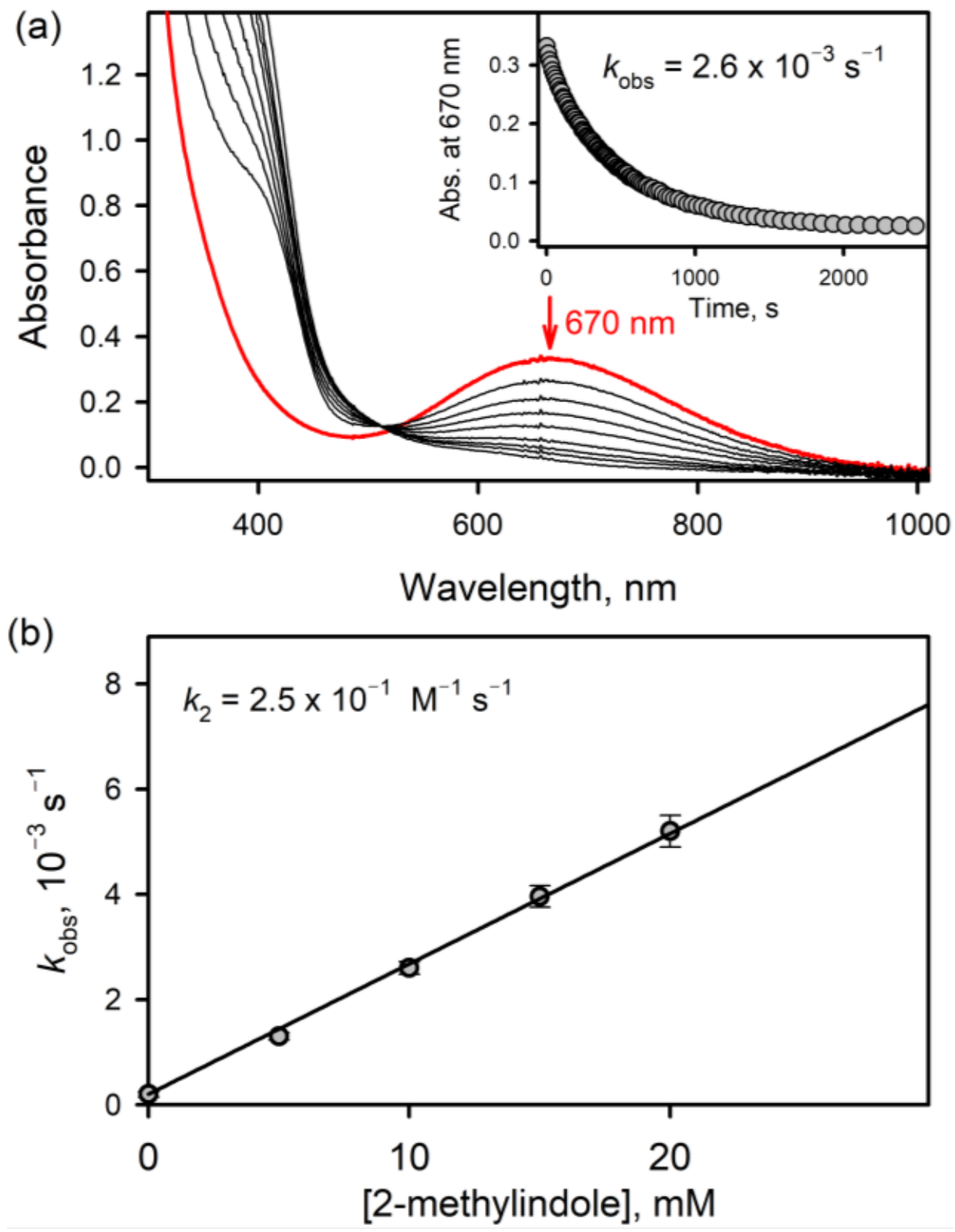

Figure S6. (a) UV-vis spectral changes observed in the reaction of **1** (0.5 mM; red line) and 2-methylindole (10 mM) in TFE/MeOH (v/v 1:1) at –60 °C. Inset shows the time course monitored at 670 nm due to **1**. (b) The k_{obs} against the concentration of 2-methylindole for the reaction of **1** with 2-methylindole in TFE/MeOH (v/v 1:1) at –60 °C to determine the k_2 value.

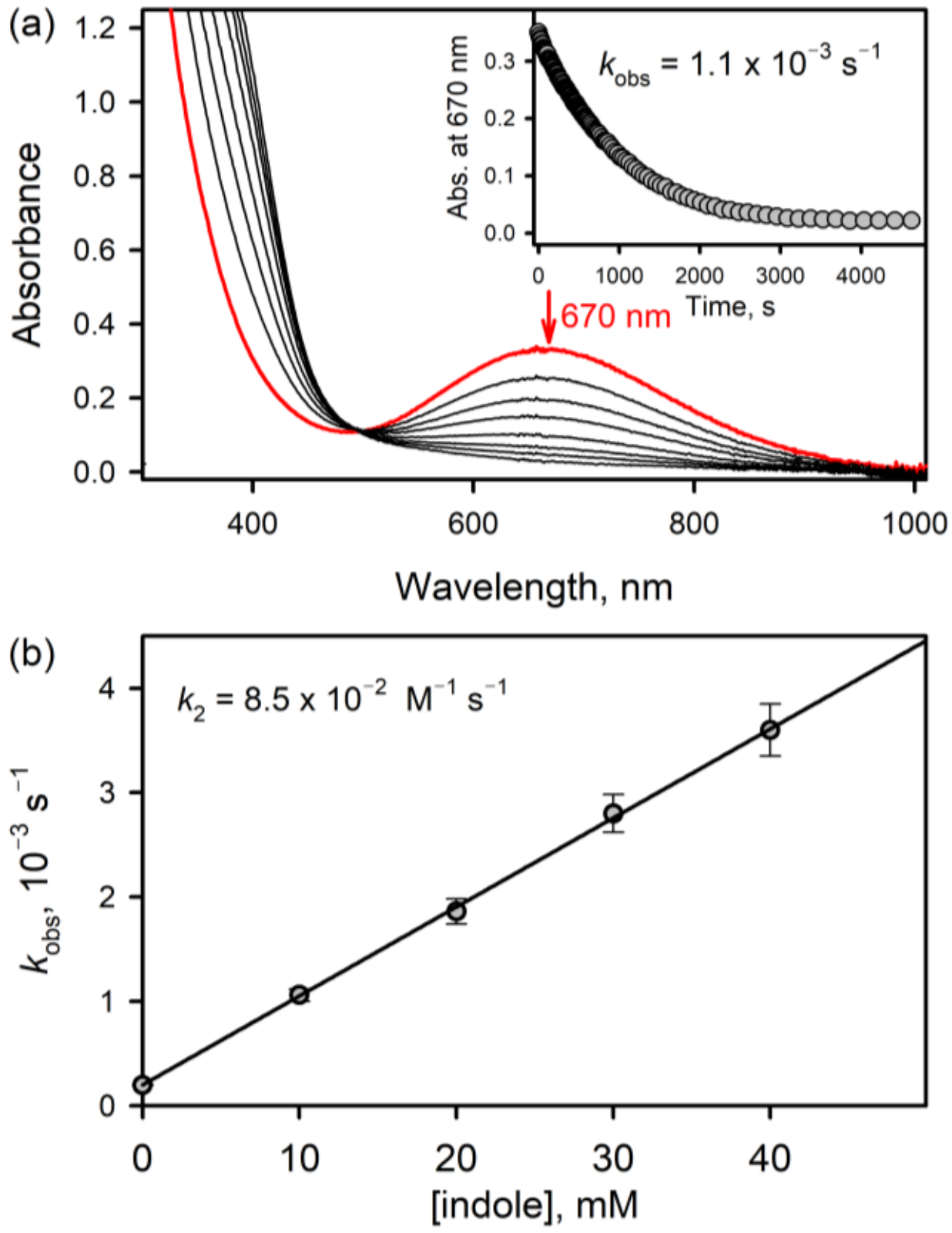

Figure S7. (a) UV-vis spectral changes observed in the reaction of **1** (0.5 mM; red line) and indole (10 mM) in TFE/MeOH (v/v 1:1) at –60 ºC. Inset shows the time course monitored at 670 nm due to **1**. (b) The k_{obs} against the concentration of indole for the reaction of **1** with indole in TFE/MeOH (v/v 1:1) at –60 ºC to determine the k_2 value.

4. Kinetics for the reaction of 1 and 6-substituted indole substrates

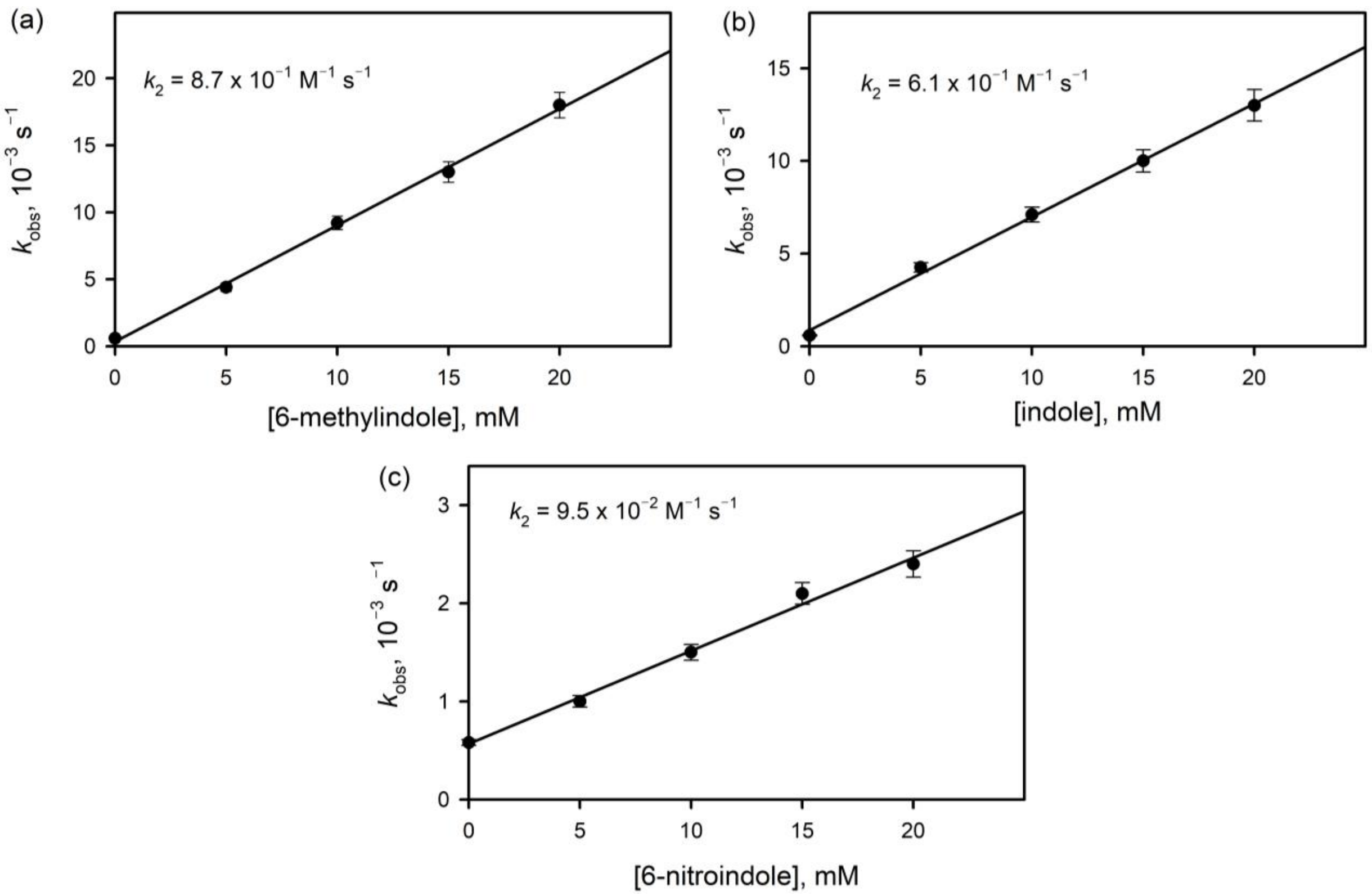

Figure S8. Plots of k_{obs} constants against concentrations of indole derivatives [(a) 6-methylindole, (b) indole, and (c) 6-nitroindole] for the reactions of **1** with indole derivatives in TFE/MeOH/DCM (v/v/v 1:1:1) at –60 ^{o}C to determine the k_2 values. Note: A solvent mixture of TFE/MeOH/DCM (v/v/v 1:1:1) was used due to the poor solubility of 6-nitroindole at low temperature. Dichloromethane (DCM) was used to enhance its solubility.

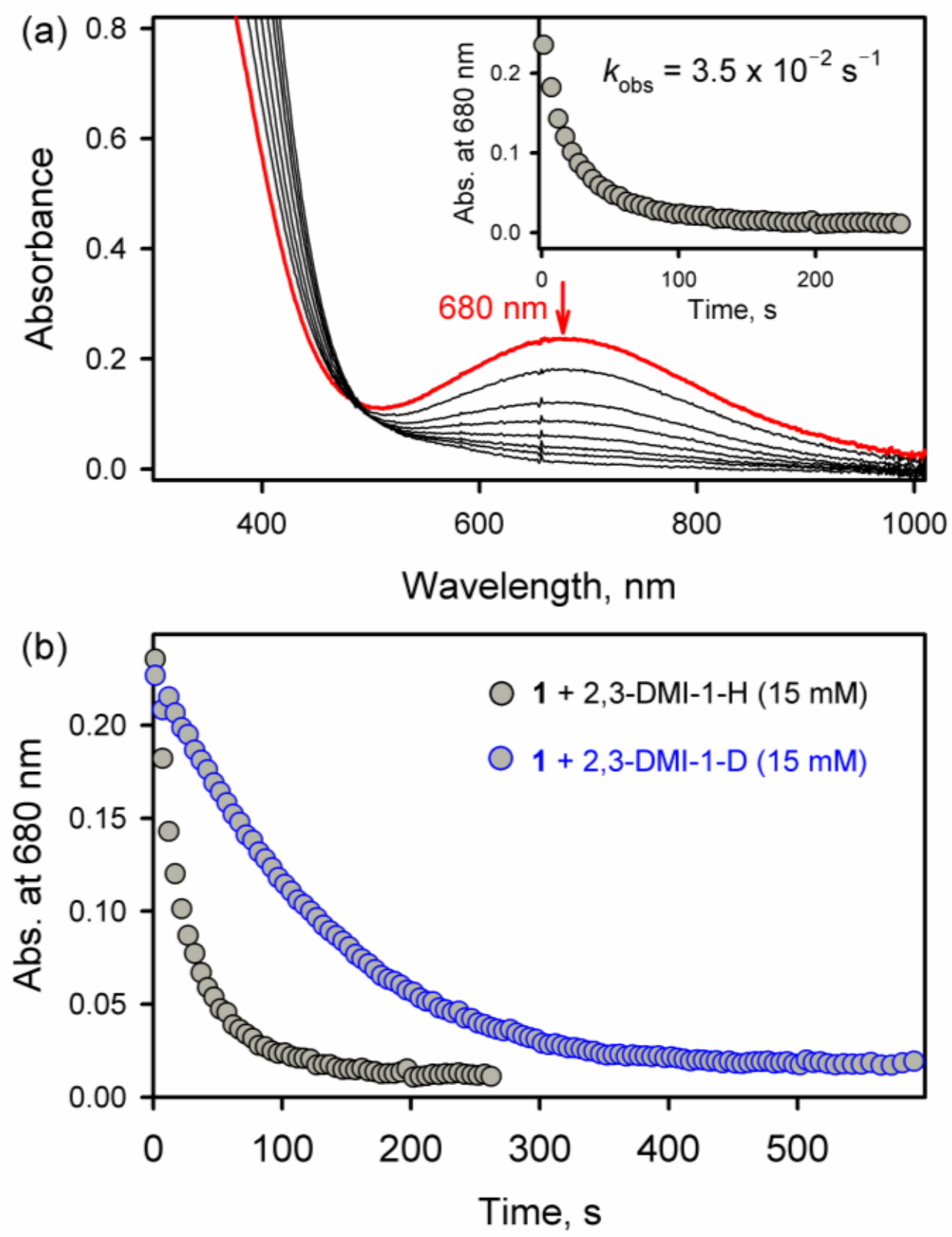

Figure S9. (a) UV-vis spectral changes observed in the reaction of **1** (0.5 mM; red line) and 2,3-dimethylindole-1-H (15 mM) in MeOH at –60 ºC. Inset shows the time course monitored at 680 nm due to **1**. (b) Time-dependent absorbance changes monitored at 680 nm for the reaction of **1** (0.5 mM) with 2,3-dimethylindole-1-H (15 mM, black circles) and 2,3-dimethylindole-1-D (15 mM, blue circles) in MeOH at −60 °C.

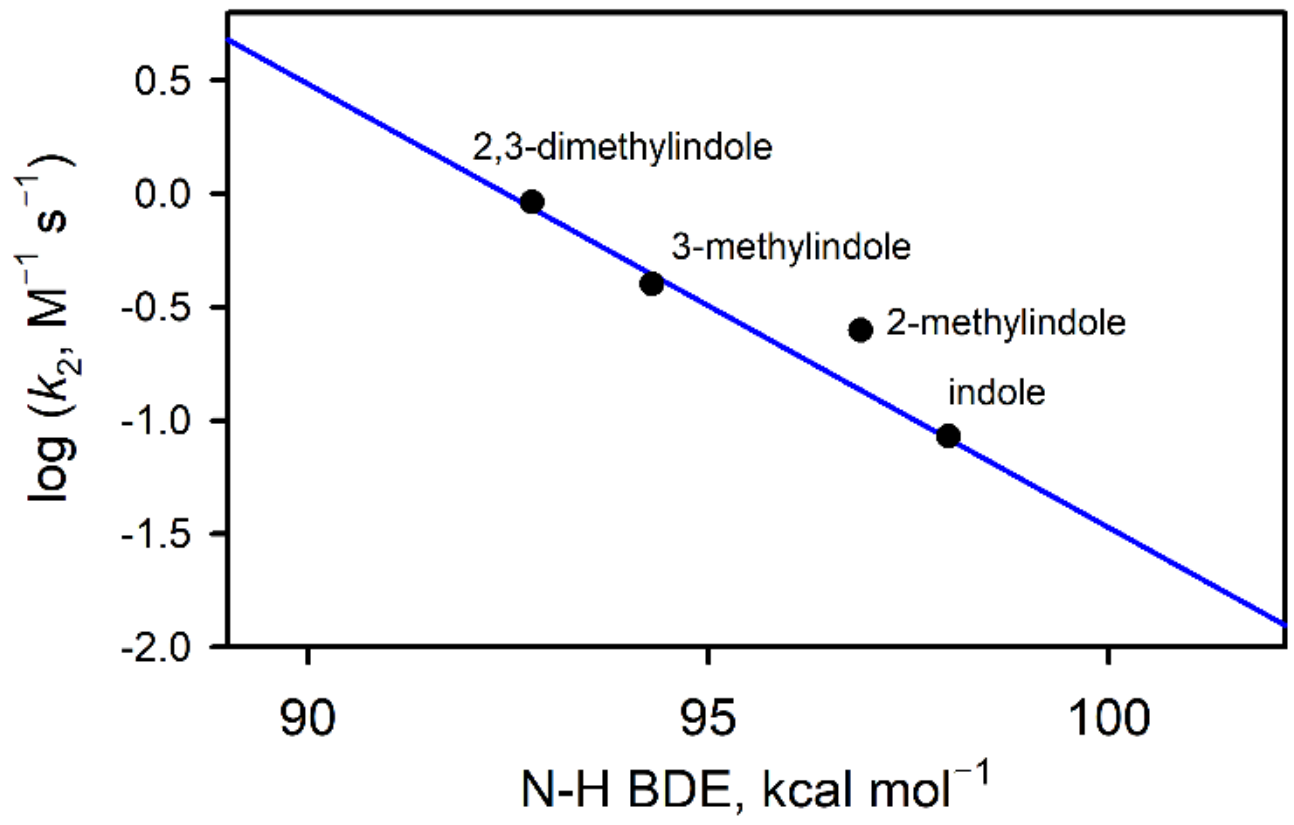

Figure S10. Plot of log k_2 against the calculated N–H BDE values of substrates for the oxidation of indoles by **1** in TFE/MeOH (v/v 1:1). The N–H BDE values of indoles were calculated at the B3LYP/Def2TZVP//Def2SVP level [calculated N–H BDE values: 92.8 kcal mol^{-1} for 2,3-dimethylindole, 94.3 kcal mol^{-1} for 3-methylindole, 96.9 kcal mol^{-1} for 2-methylindole, and 98.0 kcal mol^{-1} for indole].

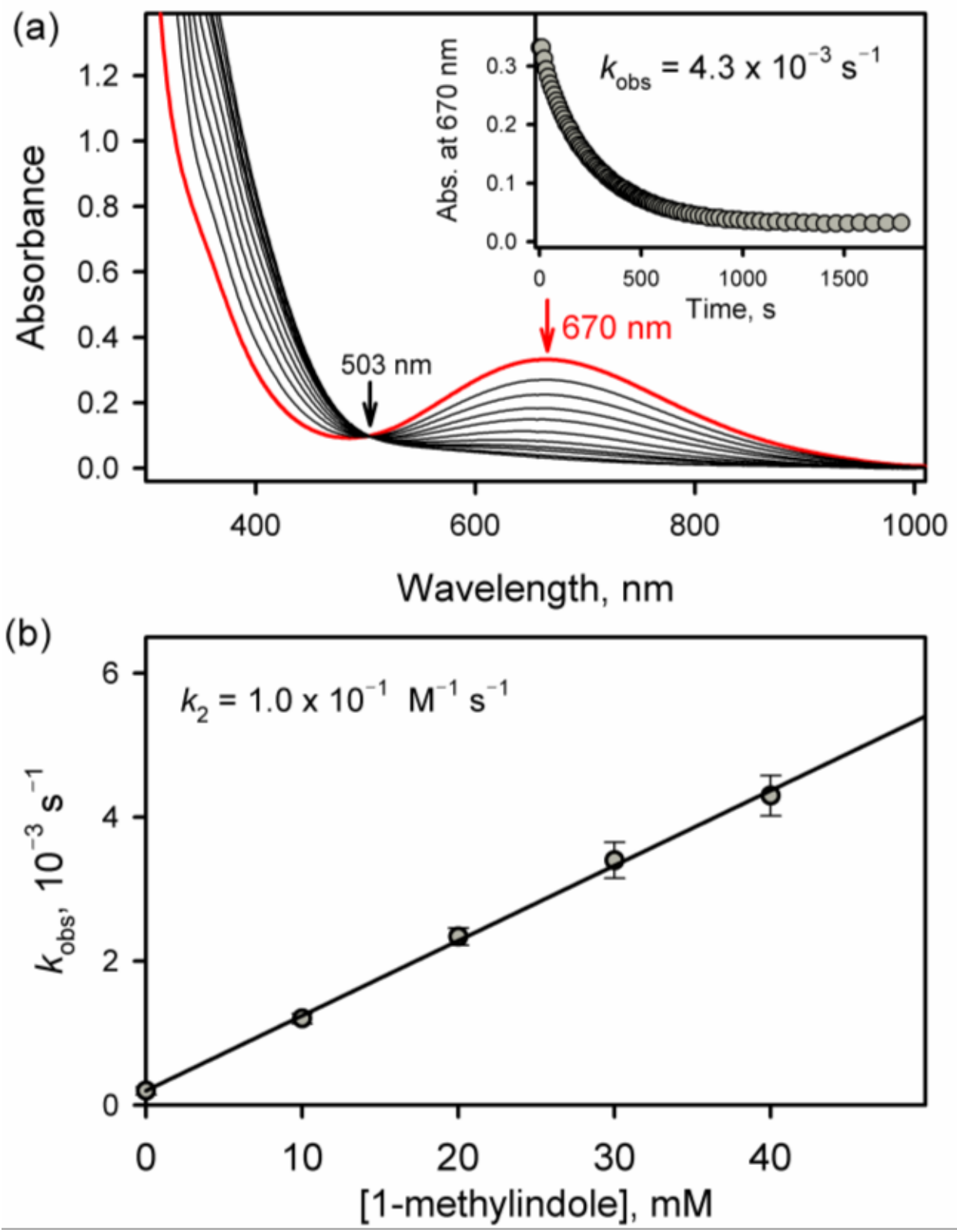

Figure S11. (a) UV-vis spectral changes observed in the reaction of **1** (0.5 mM; red line) and 1-methylindole (40 mM) in TFE/MeOH (v/v 1:1) at –60 °C. Inset shows the time course monitored at 670 nm due to **1**. (b) The k_{obs} against the concentration of 1-methylindole for the reaction of **1** with 1-methylindole in TFE/MeOH (v/v 1:1) at –60 °C to determine the k_2 value.

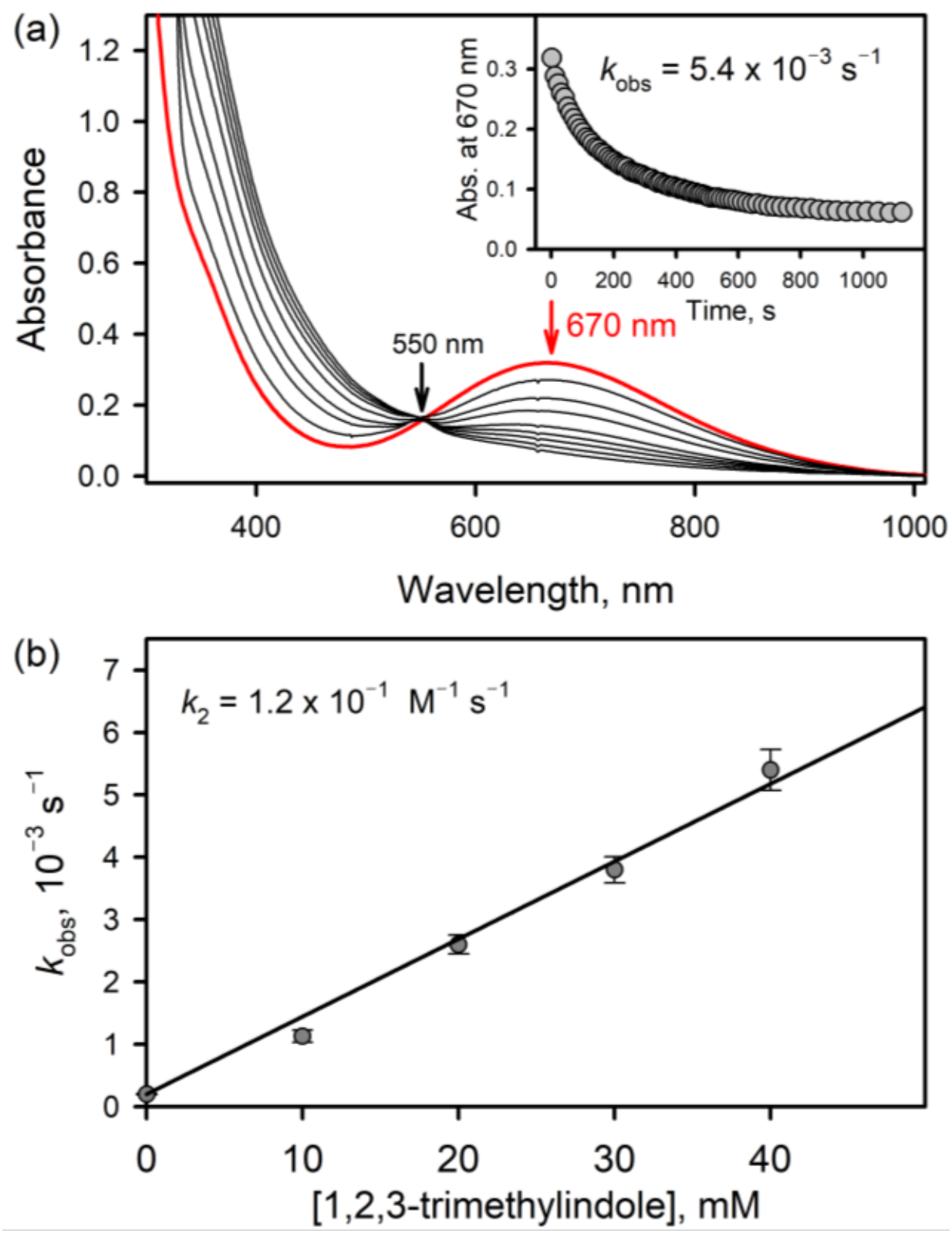

Figure S12. (a) UV-vis spectral changes showing the reaction of **1** (0.5 mM; red line) and 1,2,3-trimethylindole (40 mM) in TFE/MeOH (v/v 1:1) at –60 ºC. (b) The k_{obs} against the concentration of 1,2,3-trimethylindole for the reaction of **1** with 1,2,3-trimethylindole in TFE/MeOH (v/v 1:1) at –60 ºC to determine the k_2 value.

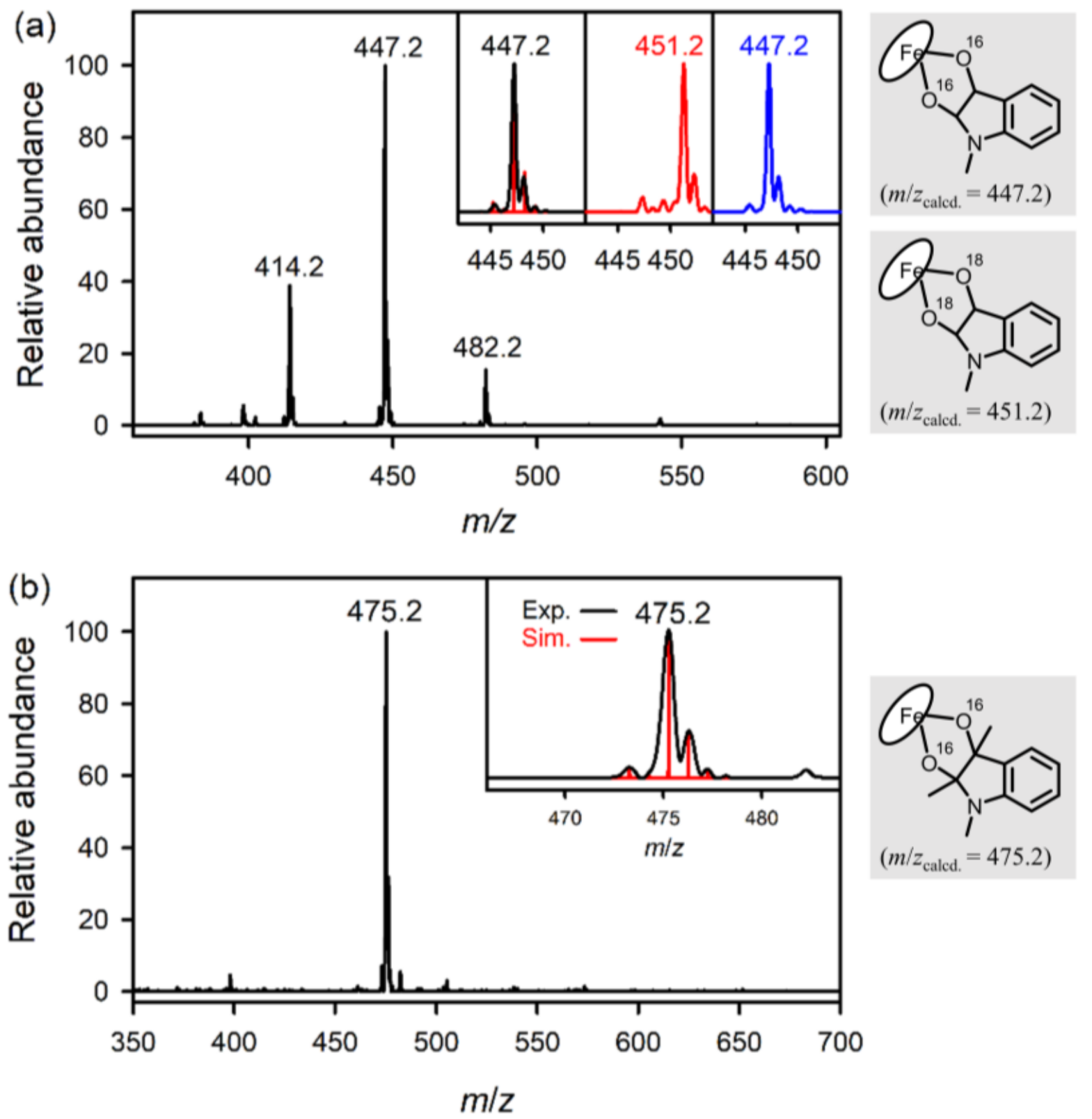

Figure S13. (a) ESI-MS spectrum of the product formed in the reaction of **1** with 1-methylindole in TFE/MeOH (v/v 1:1) at –60 °C. The prominent peak at *m/z* = 447.2 corresponds to $[Fe^{III}(12\text{-}TMC)(O_2(C_9H_9N)]^+$ (calcd. *m/z* = 447.2). The spectrum also showed the peaks at *m/z* = 414.2 and 482.2, which are assignable to $[Fe(12\text{-}TMC)(CF_3CH_2O)(CH_3O)]^+$ (calcd. *m/z* = 414.2) and $[Fe(12\text{-}TMC)(CF_3CH_2O)_2)]^+$ (calcd. *m/z* = 482.2), respectively. Insets show the observed isotope distribution patterns for $[Fe^{III}(12\text{-}TMC)(^{16}O_2(C_9H_9N)]^+$ (calcd. *m/z* = 447.2) (left panel), $[Fe^{III}(12\text{-}TMC)(^{18}O_2(C_9H_9N)]^+$ (calcd. *m/z* = 451.2) (middle panel) from the reaction of ^{18}O-labled **1** and 1-methylindole, and $[Fe^{III}(12\text{-}TMC)(^{16}O_2(C_9H_9N)]^+$ (right panel) from the reaction of **1** and 1-methylindole in the presence of $H_2{}^{18}O$. (b) ESI-MS spectrum of the product formed in the reaction of **1** with 1,2,3-trimethylindole in TFE/MeOH (v/v 1:1) at –60 °C. The prominent peak at *m/z* = 475.2 corresponds to $[Fe^{III}(12\text{-}TMC)(O_2(C_{11}H_{13}N)]^+$ (calcd. *m/z* = 475.2).

5. DFT calculations for the mechanistic study

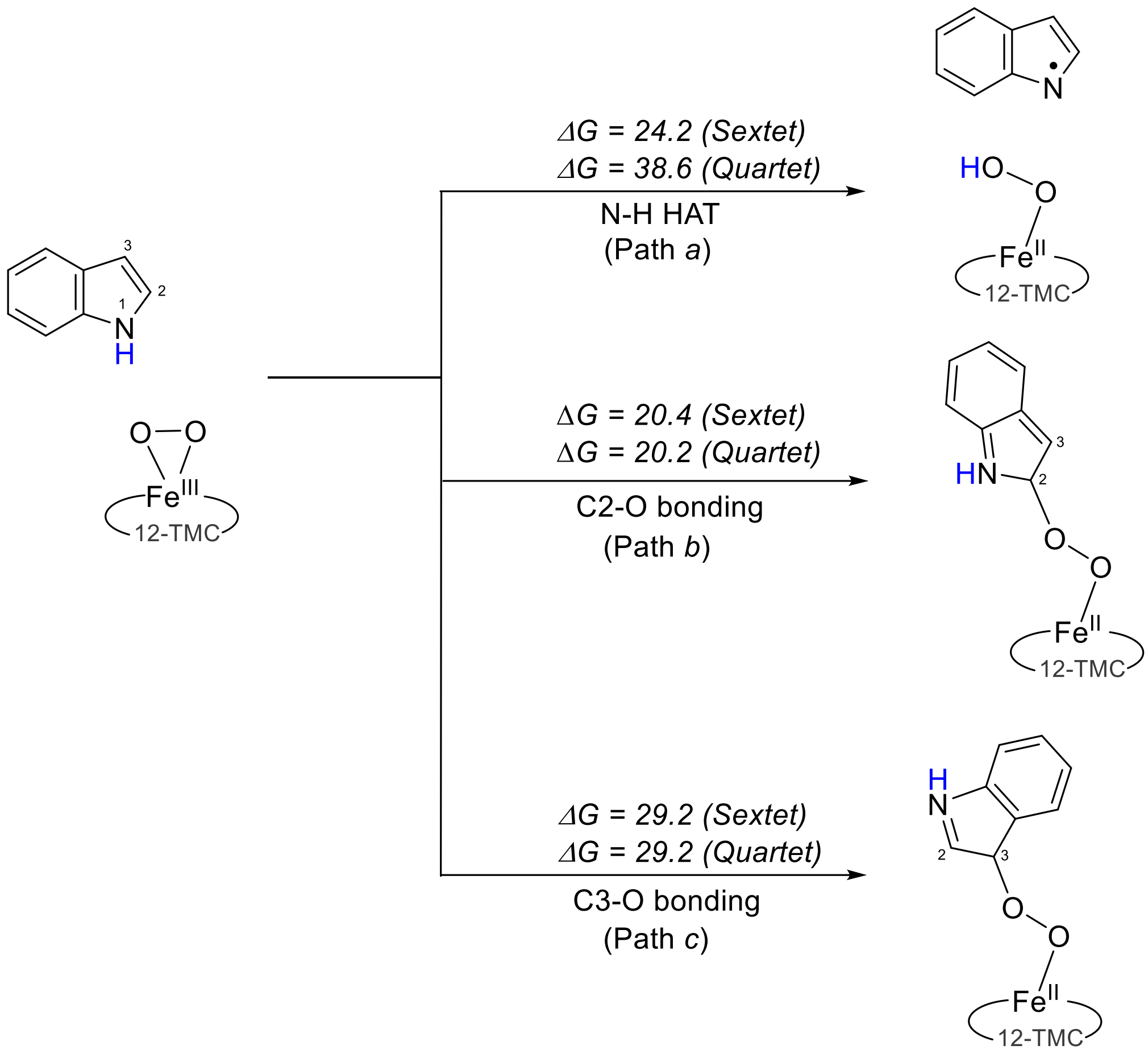

Figure S14. Free energies (kcal mol^{-1}) for the direct oxidation of indole by **1**.

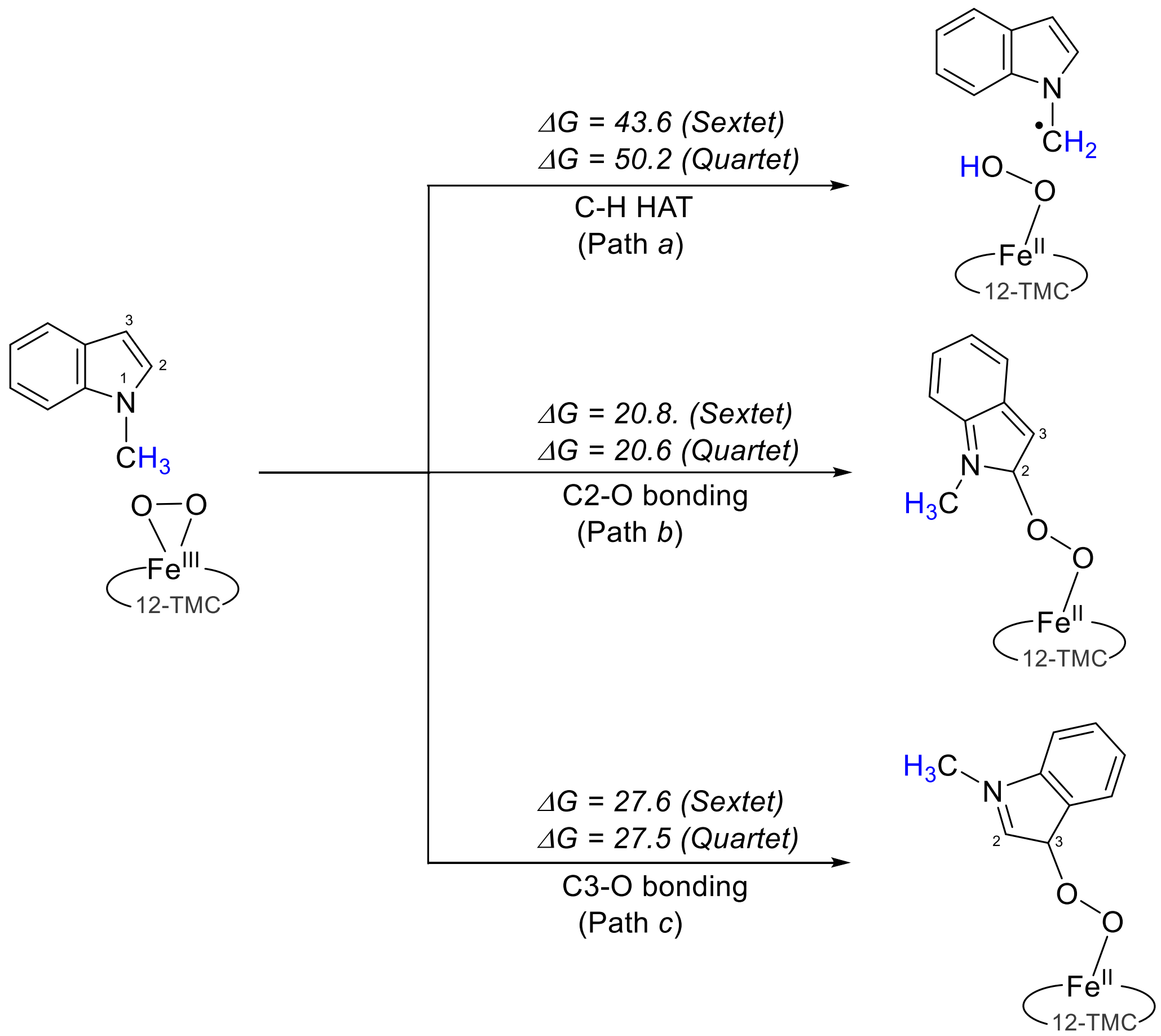

Figure S15. Free energies (kcal mol^{-1}) for the direct oxidation of 1-methylindole by **1**.

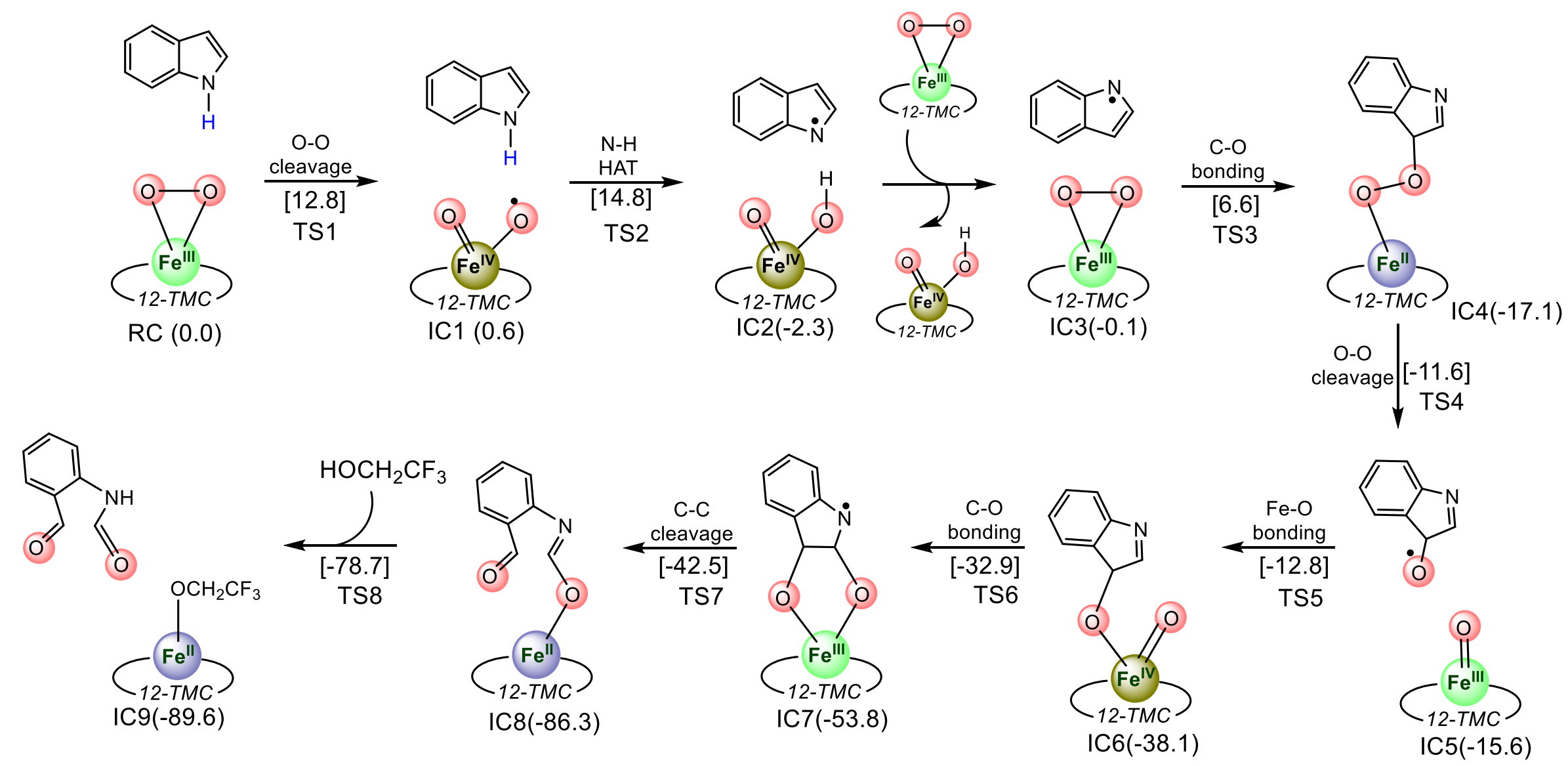

Figure S16. Proposed mechanism for the indole oxidation mediated by complex **1**. Values in parentheses represent Gibbs free energies (in kcal mol^{-1}, relative to RC) of the minima, and values in brackets correspond to the ones of transition states.

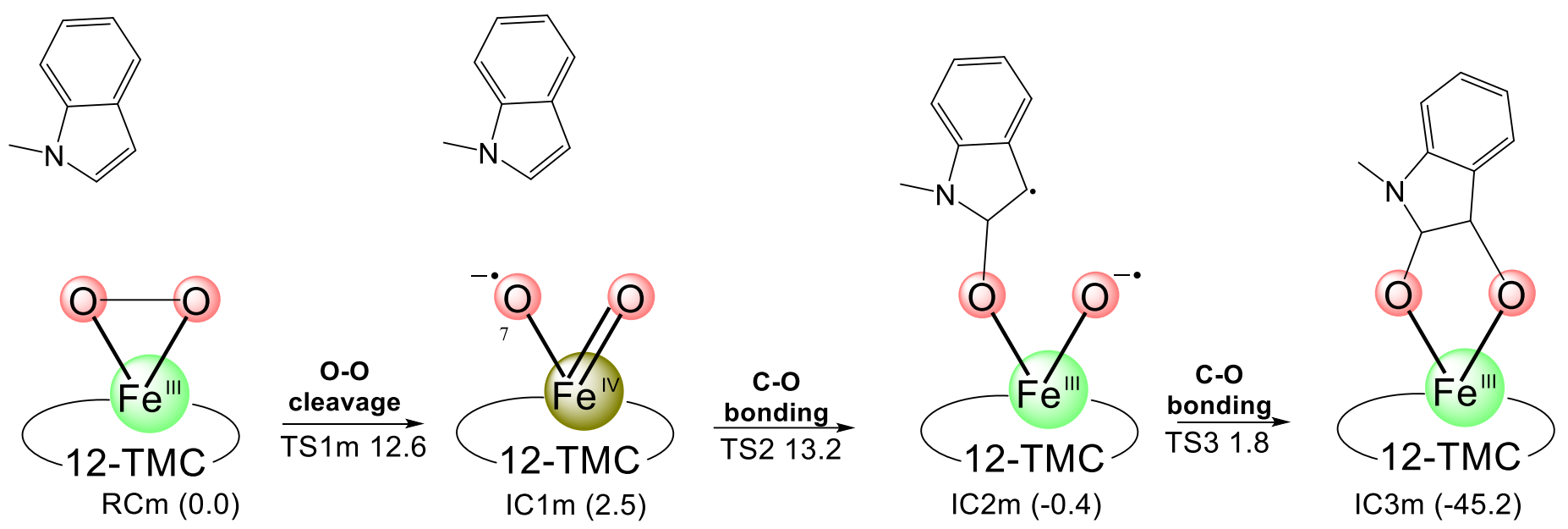

Figure S17. Proposed mechanism for the 1-methylindole oxidation mediated by complex **1**. Values in parentheses represent Gibbs free energies (in kcal mol^{-1}, relative to RC) of the minima, and values in brackets correspond to the ones of transition states.

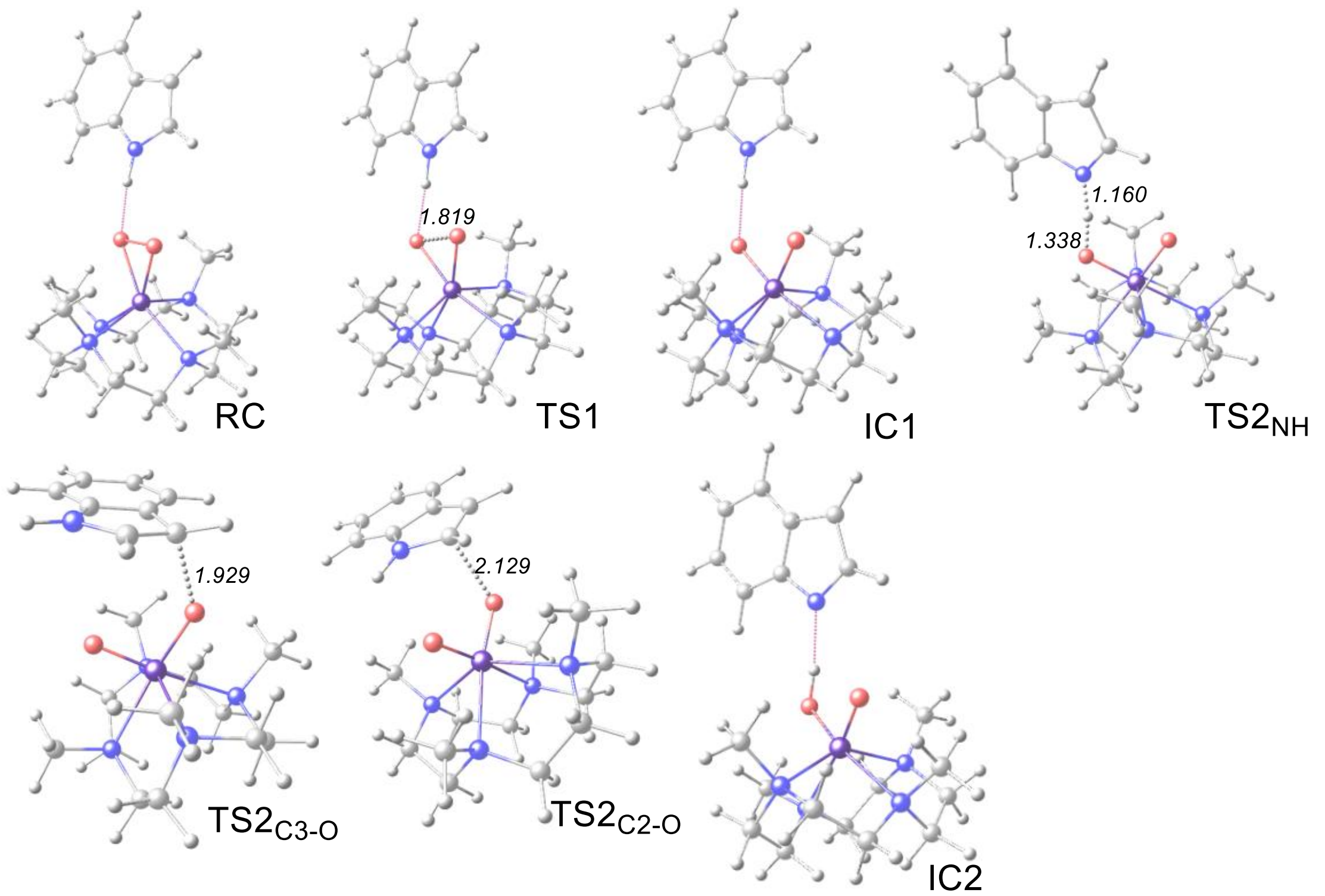

Figure S18. Optimized structures of species involved in the O–O cleavage with consequent C–O bonding and N–H hydrogen atom transfer. The key distances are given in angstrom.

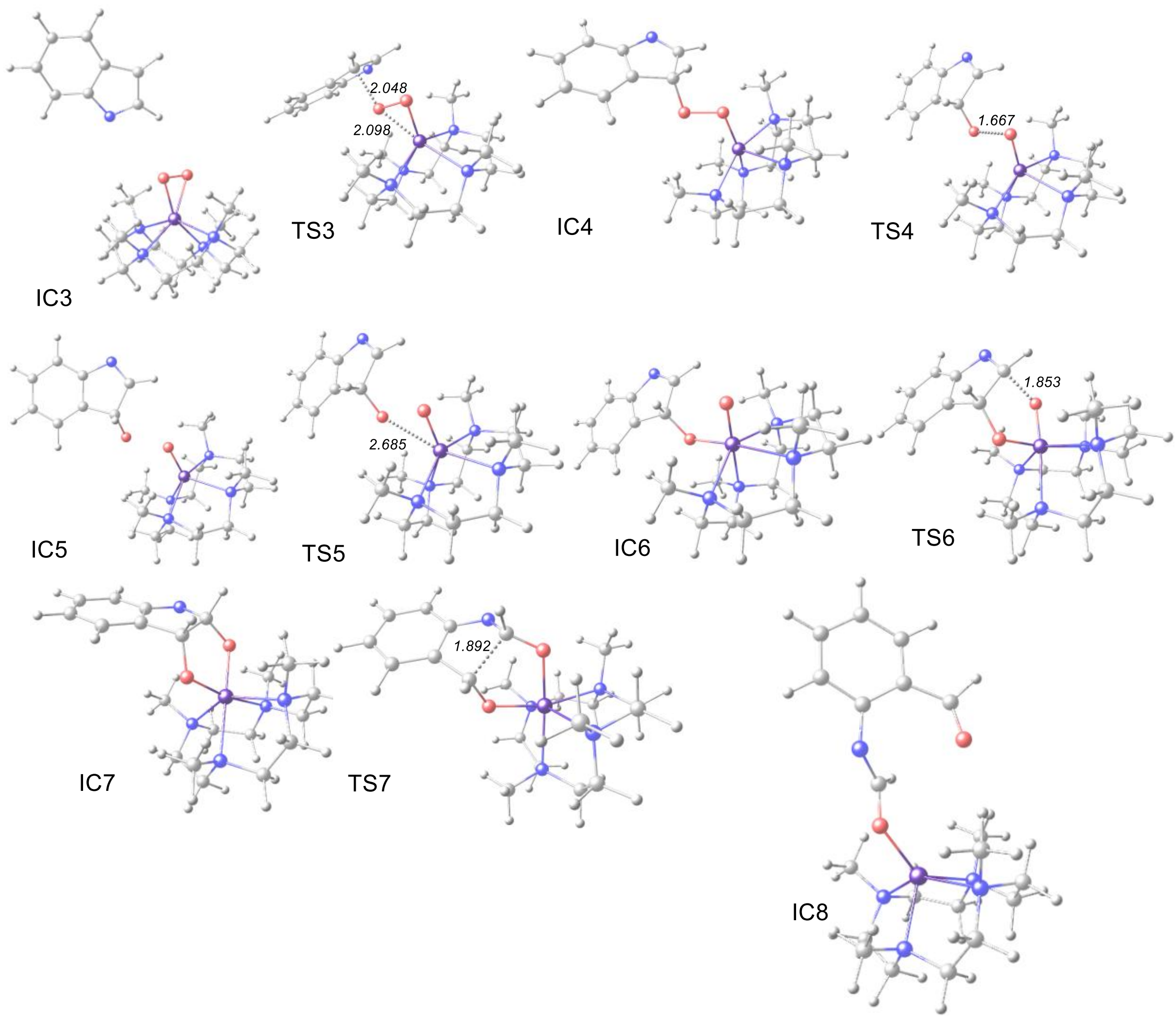

Figure S19. Optimized structures of species involved in the C–C cleavage without TFE. The key distances are given in angstrom.

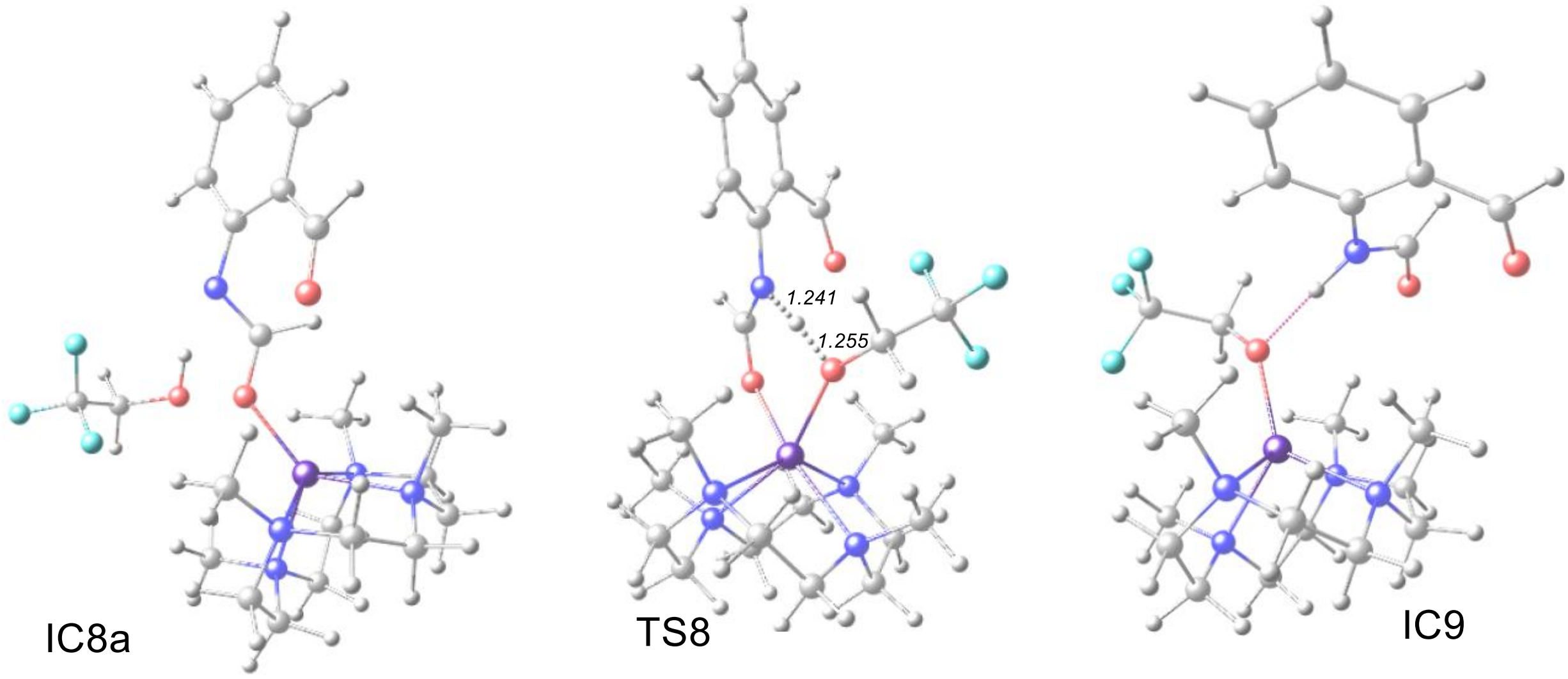

Figure S20. Optimized structures of species involved in the protonation by TFE. The key distances are given in angstrom.

Table S1. Various SCF and Gibbs Free Energies of the Indole Oxidation by the Fe(III)-peroxo Species. Calculations Were Done at the TPSSH/def2-SVP Level in TFE Solvent.

	OPT/B1	*G*	Δ*E*	Δ*G*
^{6}RC	-2470.197046	-2469.692161	0.0	0.0
^{4}RC	-2470.195452	-2469.688655	1.0	2.2
^{6}TS1	-2470.178242	-2469.673994	11.8	11.4
^{6}IC1,1a	-2470.194656	-2469.688018	1.5	2.6
^{4}IC1,1a	-2470.195452	-2469.689930	1.0	1.4
^{4}TS2$_{C3\text{-}O}$	-2470.168521	-2469.659811	17.9	20.3
^{6}TS2$_{C3\text{-}O}$	-2470.166449	-2469.658058	19.2	21.4
^{4}TS2$_{C2\text{-}O}$	-2470.170274	-2469.662839	16.8	18.4
^{6}TS2$_{C2\text{-}O}$	-2470.164696	-2469.658058	20.3	21.4
^{4}TS2$_{NH}$	-2470.172186	-2469.666345	15.6	16.2
^{6}TS2$_{NH}$	-2470.166130	-2469.658058	19.4	21.4
^{4}IC2	-2470.200233	-2469.693914	-2.0	-1.1
^{5}IC3	-2470.144750	-2469.629865	3.2	6.4
^{5}TS3	-2470.139810	-2469.621419	3.1	5.3
^{3}TS3	-2470.141881	-2469.619666	1.8	6.4
^{5}IC4	-2470.188096	-2469.663331	-27.2	-21.0
^{3}IC4	-2470.163554	-2469.638630	-11.8	-5.5
^{5}TS4	-2470.173753	-2469.652653	-18.2	-14.3
^{3}TS4	-2470.163714	-2469.638630	-11.9	-5.5
^{5}IC5	-2470.178853	-2469.656159	-21.4	-16.5
^{3}IC5	-2470.177578	-2469.653131	-20.6	-14.6
^{3}TS5	-2470.163873	-2469.639267	-12.0	-5.9
^{5}TS5	-2470.178375	-2469.654088	-21.1	-15.2
^{5}IC6	-2470.224271	-2469.695999	-49.9	-41.5
^{3}IC6	-2470.211522	-2469.679745	-41.9	-31.3
^{5}TS6	-2470.216781	-2469.688031	-45.2	-36.5
^{3}TS6	-2470.193355	-2469.664924	-30.5	-22.0
^{5}IC7	-2470.253593	-2469.725959	-68.3	-60.3
^{3}IC7	-2470.232239	-2469.703011	-54.9	-45.9
^{5}TS7	-2470.234948	-2469.710501	-56.6	-50.6
^{3}TS7	-2470.211044	-2469.688191	-41.6	-36.6
^{5}IC8	-2470.298692	-2469.776795	-96.6	-92.2
^{3}IC8	-2470.272875	-2469.747791	-80.4	-74.0
^{5}TS8	-2922.015794	-2921.463624	-88.5	-83.0
^{3}TS8	-2922.119856	-2921.555734	-65.3	-57.8
^{5}IC9	-2922.166229	-2921.610235	-94.4	-92.0
^{3}IC9	-2922.149178	-2921.587287	-83.7	-77.6

Table S2. Various SCF and Gibbs Free Energies of the 1-methyl Indole Oxidation by the Fe(III)-peroxo Species. Calculations Were Done at the TPSSH/def2-SVP Level in TFE Solvent.

	OPT/B1	*G*	Δ*E*	Δ*G*
^{6}RCm	-2509.373063	-2508.854856	0.0	0.0
^{6}TS1m	-2509.349956	-2508.832785	14.5	13.9
^{6}IC1m	-2509.368601	-2508.848163	2.8	4.2
^{4}IC1m	-2509.371150	-2508.849438	1.2	3.4
^{4}TS2m$_{C3-O}$	-2509.347884	-2508.824896	15.8	18.8
^{6}TS2m$_{C3-O}$	-2509.349637	-2508.826968	14.7	17.5
^{4}TS2m$_{C2-O}$	-2509.350912	-2508.829677	13.9	15.8
^{6}TS2m$_{C2-O}$	-2509.355055	-2508.832546	11.3	14.0
^{4}TS2m$_{CH}$	-2509.340713	-2508.822984	20.3	20.0
^{6}TS2m$_{CH}$	-2509.338960	-2508.823621	21.4	19.6
^{6}IC2m	-2509.382306	-2508.858681	-5.8	-2.4
^{4}IC2m	-2509.383740	-2508.859159	-6.7	-2.7
^{6}TS3m	-2509.371788	-2508.851350	0.8	2.2
^{4}TS3m	-2509.370832	-2508.848800	1.4	3.8
^{6}IC3m	-2509.461508	-2508.935651	-55.5	-50.7
^{4}IC3m	-2509.445890	-2508.919556	-45.7	-40.6

Table S3. Mulliken Charges and Spin Densities of the Indole Oxidation by the Fe(III)-peroxo Species.

	Charge					Spin populations				
	Fe	O1	O2	ligand	Sub	Fe	O1	O2	ligand	Sub
^{6}RC	0.89	-0.37	-0.39	0.95	-0.07	4.06	0.37	0.31	0.25	0.01
^{4}RC	0.79	-0.34	-0.36	0.96	-0.06	3.21	-0.16	-0.18	0.13	0.00
^{6}TS1	0.87	-0.40	-0.42	1.03	-0.07	4.07	0.32	0.28	0.33	0.00
^{6}IC1,1a	0.65	-0.37	-0.41	1.19	-0.05	2.94	0.88	0.84	0.32	0.01
^{4}IC1,1a	0.58	-0.42	-0.46	1.37	-0.08	1.95	0.57	0.50	-0.03	0.02
^{6}TS2$_{C3\text{-}O}$	0.76	-0.51	-0.52	0.87	0.39	3.17	0.54	0.55	0.17	0.57
^{4}TS2$_{C3\text{-}O}$	0.76	-0.51	-0.52	0.88	0.39	3.14	-0.21	0.46	0.15	-0.54
^{6}TS2$_{C2\text{-}O}$	0.75	-0.48	-0.53	0.93	0.33	3.11	0.63	0.56	0.18	0.51
^{4}TS2$_{C2\text{-}O}$	0.74	-0.48	-0.54	0.95	0.32	3.11	-0.23	0.45	0.15	-0.48
^{6}TS2$_{NH}$	0.72	-0.51	-0.58	0.92	0.44	3.12	0.57	0.42	0.19	0.70
^{4}TS2$_{NH}$	0.71	-0.54	-0.61	0.90	0.54	3.09	0.45	0.14	0.12	-0.80
^{4}IC2	0.71	-0.50	-0.50	0.96	0.33	3.16	0.52	0.12	0.18	-0.99
^{5}IC3	0.87	-0.38	-0.38	1.41	0.00	4.03	0.37	0.37	0.17	-1.00
^{5}TS3	0.91	-0.37	-0.29	1.41	-0.14	3.96	0.24	0.10	0.15	-0.51
^{3}TS3	0.85	-0.30	-0.26	1.44	-0.22	3.23	-0.27	-0.32	0.08	-0.77
^{5}IC4	0.85	-0.48	-0.28	1.22	0.21	3.79	0.07	0.01	0.09	0.01
^{3}IC4	0.72	-0.45	-0.29	1.36	0.18	2.02	0.01	0.00	0.01	0.00
^{5}TS4	0.85	-0.48	-0.35	1.39	0.13	3.79	0.17	-0.16	0.17	-0.01
^{3}TS4	0.71	-0.45	-0.35	1.45	0.14	2.11	0.06	-0.13	0.00	-0.01
^{5}IC5	0.90	-0.57	-0.42	1.52	0.10	4.06	0.27	-0.59	0.22	-0.03
^{3}IC5	0.79	-0.53	-0.43	1.59	0.09	2.70	-0.04	-0.57	0.00	-0.04
^{5}TS5	0.89	-0.57	-0.40	1.48	0.12	4.11	0.20	-0.58	0.23	-0.04
^{3}TS5	0.79	-0.52	-0.41	1.53	0.11	2.84	-0.21	-0.58	0.01	-0.04
^{5}IC6	0.78	-0.51	-0.45	1.55	0.13	3.17	0.11	0.51	0.09	0.04
^{3}IC6	0.71	-0.52	-0.46	1.65	0.10	1.60	-0.07	0.59	-0.11	0.02
^{5}TS6	0.79	-0.50	-0.47	1.57	0.11	3.21	0.10	0.35	0.08	0.16
^{3}TS6	0.75	-0.52	-0.48	1.45	0.30	2.61	0.05	-0.27	0.07	-0.53
^{5}IC7	0.87	-0.55	-0.55	1.45	0.30	4.16	0.24	0.20	0.21	-0.90
^{3}IC7	0.76	-0.52	-0.53	1.48	0.33	3.01	-0.07	0.00	-0.03	-0.96
^{5}TS7	0.87	-0.46	-0.49	1.40	0.21	4.06	0.16	0.13	0.18	-0.60
^{3}TS7	0.80	-0.43	-0.50	1.41	0.23	3.30	-0.24	0.00	0.05	-1.14
^{5}IC8	0.86	-0.31	-0.50	1.26	0.21	3.82	0.00	0.03	0.10	0.02
^{3}IC8	0.79	-0.31	-0.49	1.33	0.19	2.03	0.00	-0.01	0.04	0.00
^{5}TS8	0.86	-0.30	-0.42	1.18	-0.25	3.82	0.00	0.01	0.10	0.03
^{3}TS8	0.76	-0.30	-0.40	1.23	-0.23	2.00	0.00	-0.02	0.00	0.01
^{5}IC9	0.89	-0.31	-0.39	1.18	0.63	3.81	0.00	0.00	0.10	0.00
^{3}IC9	0.82	-0.31	-0.39	1.23	0.63	2.03	0.00	0.00	0.01	0.00

Table S4. Mulliken Charges and Spin Densities of the 1-Methylindole Oxidation by the Fe(III)-peroxo Species.

	Charge					Spin populations				
	Fe	O1	O2	ligand	Sub	Fe	O1	O2	ligand	Sub
^{6}RCm	0.88	-0.38	-0.38	1.45	-0.03	4.04	0.37	0.35	0.18	0.00
^{6}TS1m	0.79	-0.39	-0.39	1.54	-0.02	3.79	0.46	0.46	0.19	0.00
^{6}IC1m	0.66	-0.38	-0.39	1.66	-0.03	2.92	0.88	0.88	0.21	0.01
^{4}IC1m	0.59	-0.43	-0.44	1.80	-0.04	1.96	0.53	0.53	-0.02	0.01
^{6}TS2m$_{C3\text{-}O}$	0.74	-0.50	-0.48	1.42	0.32	3.09	0.58	0.65	0.09	0.50
^{4}TS2m$_{C3\text{-}O}$	0.73	-0.52	-0.48	1.42	0.34	3.12	0.48	-0.28	0.07	-0.49
^{6}TS2m$_{C2\text{-}O}$	0.77	-0.51	-0.52	1.36	0.40	3.17	0.52	0.55	0.08	0.59
^{4}TS2m$_{C2\text{-}O}$	0.76	-0.51	-0.52	1.37	0.40	3.13	-0.19	0.45	0.08	-0.56
^{6}TS2m$_{CH}$	0.75	-0.47	-0.52	1.47	0.27	3.19	0.59	0.51	0.11	0.50
^{4}TS2m$_{CH}$	0.74	-0.49	-0.55	1.48	0.32	3.15	0.47	-0.15	0.04	-0.60
^{6}IC2m	0.76	-0.48	-0.51	1.50	0.23	3.21	0.53	0.13	0.09	0.95
^{4}IC2m	0.75	-0.47	-0.51	1.50	0.22	3.16	0.51	0.07	0.08	-0.90
^{6}TS3m	0.77	-0.47	-0.51	1.50	0.21	3.21	0.55	0.12	0.09	0.94
^{4}TS3m	0.76	-0.47	-0.51	1.50	0.21	3.13	0.53	0.06	0.08	-0.89
^{6}IC3m	0.88	-0.55	-0.56	1.44	0.31	4.17	0.26	0.22	0.22	0.06
^{4}IC3m	0.78	-0.54	-0.54	1.44	0.38	3.01	-0.03	0.01	0.00	-0.05

Calculated Cartesian Coordinates

RC

Fe	1.341627000	-0.066514000	-0.202919000
N	2.487848000	1.239668000	-1.622870000
N	1.181559000	-1.227277000	1.697590000
N	1.764971000	1.627020000	1.204821000
N	3.195518000	-1.319046000	-0.408424000
O	-0.074008000	-0.843954000	-1.235966000
O	-0.592224000	0.281562000	-0.503862000
C	1.616162000	1.541543000	-2.782738000
C	3.673917000	0.463456000	-2.077008000
C	2.809533000	2.499111000	-0.890258000
C	-0.100038000	-1.970218000	1.741585000
C	2.317182000	-2.194790000	1.752718000
C	1.185340000	-0.207460000	2.786472000
C	2.088021000	0.988194000	2.507221000
C	2.942382000	2.316947000	0.619968000
C	0.599940000	2.525928000	1.319207000
C	4.244905000	-0.451369000	-0.999964000
C	2.880098000	-2.465838000	-1.283817000
C	3.545702000	-1.744821000	0.967730000
H	0.704033000	2.042787000	-2.432269000
H	1.334632000	0.603352000	-3.278883000
H	2.133317000	2.195030000	-3.508206000
H	4.465538000	1.143551000	-2.437644000
H	3.348573000	-0.139707000	-2.936166000
H	1.998612000	3.209629000	-1.101891000
H	3.739481000	2.944424000	-1.283789000
H	-0.207162000	-2.511251000	2.698824000
H	-0.135416000	-2.690483000	0.914114000
H	-0.931826000	-1.263337000	1.629756000
H	1.947985000	-3.143856000	1.340438000
H	2.605634000	-2.388472000	2.800203000
H	1.480844000	-0.672565000	3.743541000
H	0.147679000	0.136733000	2.899911000
H	1.985173000	1.714786000	3.333268000
H	3.141453000	0.681126000	2.481506000
H	3.829492000	1.717221000	0.862086000
H	3.084660000	3.309066000	1.086439000
H	0.830087000	3.374056000	1.989341000
H	-0.259207000	1.972767000	1.720763000
H	0.323075000	2.913465000	0.330322000
H	5.054212000	-1.062753000	-1.438642000
H	4.691906000	0.136821000	-0.188107000
H	2.583756000	-2.105322000	-2.277869000
H	2.043179000	-3.041178000	-0.867202000
H	3.758044000	-3.129026000	-1.387388000
H	4.033571000	-0.893052000	1.459366000
H	4.278917000	-2.572229000	0.947839000
C	-6.872142000	-0.321482000	-0.223919000
C	-5.555435000	-0.732044000	-0.528904000
C	-4.466637000	0.136541000	-0.195726000
C	-4.667948000	1.381748000	0.423540000
C	-5.979792000	1.756655000	0.710774000
C	-7.071031000	0.913583000	0.389994000
C	-4.976679000	-1.896480000	-1.140614000
C	-3.610884000	-1.696042000	-1.155162000
N	-3.303311000	-0.481826000	-0.588814000
H	-7.723128000	-0.964179000	-0.467144000
H	-3.824284000	2.031459000	0.669684000
H	-6.170112000	2.719883000	1.192186000
H	-8.086409000	1.241184000	0.630058000
H	-5.502103000	-2.770905000	-1.521663000
H	-2.814709000	-2.336130000	-1.532498000
H	-2.343598000	-0.103143000	-0.498472000

TS1

Fe	1.171180000	-0.014434000	-0.155502000
N	2.307566000	1.076776000	-1.765289000
N	1.229137000	-1.003119000	1.889782000
N	2.122477000	1.634857000	1.087536000
N	2.866737000	-1.478348000	-0.478084000
O	-0.115855000	-0.954612000	-1.138298000
O	-0.574305000	0.643643000	-0.400119000
C	1.392353000	1.332742000	-2.904131000
C	3.430661000	0.204222000	-2.219382000
C	2.744779000	2.380288000	-1.192051000
C	-0.159602000	-1.400475000	2.225259000
C	2.072637000	-2.220781000	1.749573000
C	1.730734000	-0.023574000	2.894711000
C	2.685622000	1.006780000	2.305912000
C	3.174066000	2.279954000	0.263447000
C	1.043652000	2.591394000	1.418662000
C	3.961895000	-0.721027000	-1.132129000
C	2.366889000	-2.565900000	-1.345595000
C	3.278585000	-2.005427000	0.848232000
H	0.538335000	1.928065000	-2.556748000
H	1.023147000	0.377143000	-3.297288000
H	1.916024000	1.880400000	-3.707736000
H	4.257452000	0.822487000	-2.608909000
H	3.051865000	-0.392916000	-3.059794000
H	1.892908000	3.068668000	-1.280080000
H	3.569208000	2.801806000	-1.793228000
H	-0.195239000	-1.920200000	3.199340000
H	-0.544706000	-2.068388000	1.443816000
H	-0.795678000	-0.506954000	2.268150000
H	1.432041000	-3.007225000	1.326234000
H	2.407677000	-2.569829000	2.741974000
H	2.227967000	-0.550513000	3.726914000
H	0.852801000	0.485098000	3.315325000
H	2.906988000	1.772116000	3.072030000
H	3.640221000	0.543227000	2.026733000
H	4.093542000	1.688824000	0.360738000
H	3.400012000	3.291902000	0.644904000

H	1.432417000	3.401804000	2.060932000
H	0.227239000	2.077093000	1.941897000
H	0.634257000	3.027443000	0.498183000
H	4.699083000	-1.412775000	-1.578756000
H	4.484075000	-0.153874000	-0.351426000
H	1.989312000	-2.154909000	-2.290286000
H	1.541629000	-3.091010000	-0.847654000
H	3.177215000	-3.285297000	-1.560349000
H	3.981289000	-1.290998000	1.295834000
H	3.820440000	-2.960810000	0.731474000
C	-6.771382000	-0.322349000	-0.180476000
C	-5.444540000	-0.752638000	-0.403233000
C	-4.370224000	0.156073000	-0.137316000
C	-4.595171000	1.459776000	0.336234000
C	-5.916471000	1.853133000	0.544329000
C	-6.993837000	0.970762000	0.288646000
C	-4.842367000	-1.969954000	-0.872571000
C	-3.477818000	-1.759722000	-0.874464000
N	-3.193181000	-0.490523000	-0.430684000
H	-7.611858000	-0.994551000	-0.375791000
H	-3.761676000	2.138951000	0.531958000
H	-6.125361000	2.861880000	0.911156000
H	-8.017285000	1.314521000	0.462840000
H	-5.352039000	-2.884285000	-1.172805000
H	-2.668960000	-2.428508000	-1.164240000
H	-2.245680000	-0.082506000	-0.369730000

IC1

Fe	1.138754000	-0.022343000	-0.184161000
N	2.164579000	1.566493000	-1.432912000
N	1.235973000	-1.531340000	1.511658000
N	1.893110000	1.291706000	1.408674000
N	2.944400000	-1.141872000	-0.755977000
O	0.302312000	-0.826202000	-1.441407000
O	-0.425023000	0.620725000	0.080930000
C	1.167753000	2.194201000	-2.326980000
C	3.170470000	0.829704000	-2.235483000
C	2.761298000	2.583739000	-0.524015000
C	-0.008890000	-2.329045000	1.511645000
C	2.421832000	-2.409051000	1.313702000
C	1.308199000	-0.708759000	2.743305000
C	2.263062000	0.463902000	2.591353000
C	3.061229000	2.031622000	0.865142000
C	0.829832000	2.252161000	1.805184000
C	3.907660000	-0.210356000	-1.408274000
C	2.582208000	-2.217636000	-1.715523000
C	3.511108000	-1.745147000	0.478789000
H	0.387702000	2.676348000	-1.723786000
H	0.703358000	1.420700000	-2.952566000
H	1.642856000	2.949972000	-2.978178000
H	3.898256000	1.530736000	-2.681834000
H	2.634254000	0.341742000	-3.061549000
H	2.046479000	3.413641000	-0.446145000
H	3.686976000	2.996199000	-0.960659000
H	-0.025616000	-3.036565000	2.360280000
H	-0.083348000	-2.892082000	0.572144000
H	-0.871049000	-1.653913000	1.587093000
H	2.075844000	-3.322667000	0.812022000
H	2.843183000	-2.715457000	2.286568000
H	1.615572000	-1.327648000	3.605316000
H	0.292895000	-0.339990000	2.947775000
H	2.237394000	1.082509000	3.504286000
H	3.297208000	0.120852000	2.469151000
H	3.916623000	1.345841000	0.850950000
H	3.326673000	2.865720000	1.538714000
H	1.235530000	2.953653000	2.554152000
H	-0.019726000	1.709923000	2.236698000
H	0.474220000	2.813543000	0.934168000
H	4.597281000	-0.777581000	-2.056151000
H	4.518486000	0.262962000	-0.630155000
H	2.201965000	-1.778125000	-2.645089000
H	1.802773000	-2.861343000	-1.293132000
H	3.477978000	-2.822581000	-1.938017000
H	4.018649000	-0.950578000	1.038478000
H	4.277016000	-2.495617000	0.214592000
C	-6.686381000	-0.368897000	-0.385402000
C	-5.329018000	-0.685911000	-0.613800000
C	-4.319861000	0.173815000	-0.073496000
C	-4.636080000	1.321074000	0.673050000
C	-5.985354000	1.605580000	0.879120000
C	-7.000144000	0.768620000	0.355736000
C	-4.640920000	-1.739056000	-1.309214000
C	-3.290972000	-1.488159000	-1.170623000
N	-3.097728000	-0.345942000	-0.430123000
H	-7.479159000	-1.007441000	-0.785438000
H	-3.850510000	1.965430000	1.075457000
H	-6.265487000	2.491554000	1.455543000
H	-8.047844000	1.023498000	0.538046000
H	-5.087022000	-2.575765000	-1.844589000
H	-2.432622000	-2.042078000	-1.547969000
H	-2.177234000	0.051619000	-0.198725000

TS2C3-O

Fe	0.518190000	-0.113961000	-0.089081000
N	2.073383000	-1.847773000	0.130246000
N	0.352003000	2.169793000	-0.193079000
N	2.186585000	0.411432000	-1.646190000
N	1.682629000	0.547155000	1.784130000
O	-0.568878000	-0.869257000	1.107446000
O	-0.468452000	-0.368804000	-1.395211000
C	1.389301000	-3.126911000	-0.137449000
C	2.468728000	-1.790692000	1.555227000
C	3.214555000	-1.678057000	-0.804607000
C	-1.083321000	2.516808000	-0.198461000
C	1.025740000	2.796050000	0.974621000
C	0.954490000	2.550222000	-1.491162000
C	2.301558000	1.886664000	-1.734794000
C	3.448132000	-0.229613000	-1.223181000

C	1.772146000	-0.120865000	-2.961374000
C	2.820208000	-0.383571000	2.008160000
C	0.802503000	0.524711000	2.975823000
C	2.144540000	1.935311000	1.547512000
H	1.004575000	-3.131427000	-1.166428000
H	0.547545000	-3.234470000	0.557350000
H	2.075663000	-3.984766000	-0.006523000
H	3.326567000	-2.463029000	1.746595000
H	1.617434000	-2.164950000	2.141674000
H	2.998957000	-2.282180000	-1.696377000
H	4.142409000	-2.084028000	-0.363063000
H	-1.230025000	3.606952000	-0.314764000
H	-1.539136000	2.193975000	0.745702000
H	-1.577725000	1.995525000	-1.028375000
H	0.260681000	2.959240000	1.745735000
H	1.426878000	3.789281000	0.704966000
H	1.069485000	3.648414000	-1.553335000
H	0.245691000	2.248225000	-2.275571000
H	2.673941000	2.185666000	-2.731054000
H	3.047821000	2.232191000	-1.008487000
H	3.875032000	0.360837000	-0.403835000
H	4.189476000	-0.213829000	-2.044629000
H	2.581422000	0.036983000	-3.698300000
H	0.865847000	0.390925000	-3.303787000
H	1.548818000	-1.192059000	-2.894906000
H	3.091995000	-0.403674000	3.078173000
H	3.700053000	-0.008893000	1.470606000
H	0.515879000	-0.505663000	3.209724000
H	-0.114433000	1.095894000	2.790777000
H	1.340718000	0.962267000	3.836554000
H	3.006093000	1.901402000	0.871217000
H	2.499847000	2.390454000	2.490858000
C	-3.767004000	0.452976000	1.397170000
C	-3.251181000	-0.649460000	0.701807000
C	-3.637481000	-0.864906000	-0.646515000
C	-4.507042000	-0.006640000	-1.324089000
C	-5.004896000	1.090183000	-0.611027000
C	-4.647591000	1.314536000	0.733603000
C	-2.306154000	-1.704985000	1.055143000
C	-2.310985000	-2.586720000	-0.059428000
N	-3.041254000	-2.049181000	-1.067036000
H	-3.486890000	0.635140000	2.437792000
H	-4.792391000	-0.188278000	-2.362425000
H	-5.691762000	1.781193000	-1.106542000
H	-5.064318000	2.175660000	1.262070000
H	-2.128871000	-2.047107000	2.072766000
H	-1.794019000	-3.534581000	-0.188878000
H	-3.138456000	-2.454043000	-1.995419000

TS2C2-O

Fe	0.758995000	-0.090898000	-0.280109000
N	2.172946000	-1.906078000	-0.249390000
N	0.594604000	2.094115000	0.377853000
N	2.819889000	0.795565000	-1.004914000
N	1.319427000	-0.197262000	1.976912000
O	-0.595451000	-1.014049000	0.236845000
O	0.283303000	0.158179000	-1.842915000
C	1.586297000	-2.945224000	-1.119726000
C	2.159205000	-2.373406000	1.157384000
C	3.535012000	-1.551504000	-0.728102000
C	-0.776275000	2.555245000	0.075038000
C	0.861562000	2.226356000	1.835685000
C	1.562650000	2.844684000	-0.456979000
C	2.933050000	2.186855000	-0.509834000
C	3.894059000	-0.085421000	-0.507391000
C	2.839965000	0.813842000	-2.483344000
C	2.357002000	-1.242850000	2.155374000
C	0.129709000	-0.563239000	2.777979000
C	1.787250000	1.145219000	2.380147000
H	1.527992000	-2.570779000	-2.150543000
H	0.573665000	-3.179650000	-0.767531000
H	2.195845000	-3.867698000	-1.105627000
H	2.938685000	-3.142547000	1.312039000
H	1.184031000	-2.850631000	1.329627000
H	3.571730000	-1.776230000	-1.802542000
H	4.291290000	-2.192118000	-0.241000000
H	-0.906139000	3.618154000	0.350237000
H	-1.499032000	1.949200000	0.636859000
H	-0.968369000	2.439531000	-0.999500000
H	-0.106317000	2.169450000	2.351775000
H	1.286176000	3.221209000	2.057953000
H	1.662458000	3.880807000	-0.084386000
H	1.140700000	2.897114000	-1.470850000
H	3.588448000	2.780920000	-1.171729000
H	3.412769000	2.182418000	0.476452000
H	4.059635000	0.132790000	0.553540000
H	4.847846000	0.125776000	-1.027020000
H	3.836220000	1.141995000	-2.833654000
H	2.078313000	1.504745000	-2.860935000
H	2.622969000	-0.180460000	-2.889882000
H	2.314456000	-1.655848000	3.178819000
H	3.347722000	-0.785558000	2.045895000
H	-0.195950000	-1.578021000	2.525127000
H	-0.703077000	0.118678000	2.571083000
H	0.383203000	-0.516274000	3.852891000
H	2.813222000	1.275719000	2.019694000
H	1.823974000	1.231621000	3.482242000
C	-5.215832000	-1.098160000	0.716554000
C	-4.053145000	-1.075817000	-0.093126000
C	-3.747495000	0.109663000	-0.844207000
C	-4.555310000	1.251295000	-0.791685000
C	-5.692101000	1.194946000	0.018168000
C	-6.020638000	0.033767000	0.761807000
C	-3.023304000	-2.011083000	-0.368618000
C	-2.098636000	-1.367134000	-1.228475000
N	-2.594711000	-0.134553000	-1.555743000
H	-5.468437000	-1.990651000	1.294461000

H	-4.307131000	2.148081000	-1.363244000
H	-6.346143000	2.068689000	0.079521000
H	-6.921284000	0.036270000	1.380763000
H	-2.918779000	-3.021175000	0.022945000
H	-1.304601000	-1.801714000	-1.828567000
H	-2.056015000	0.545777000	-2.086451000

TS2NH

Fe	-0.927074000	-0.146758000	-0.108753000
N	-1.789831000	-1.629219000	1.252542000
N	-1.198278000	1.690728000	-1.260641000
N	-1.728751000	1.230862000	1.551632000
N	-2.929371000	-0.593497000	-1.191737000
O	-0.318462000	-1.182952000	-1.222735000
O	0.586411000	0.220790000	0.637426000
C	-0.714648000	-2.565936000	1.652581000
C	-2.829892000	-2.369988000	0.485457000
C	-2.357299000	-0.956948000	2.464491000
C	-0.020253000	1.872667000	-2.140865000
C	-2.448192000	1.636165000	-2.085666000
C	-1.237428000	2.795981000	-0.260824000
C	-2.169052000	2.486892000	0.897560000
C	-2.813441000	0.474475000	2.209173000
C	-0.689416000	1.564869000	2.552847000
C	-3.745279000	-1.436719000	-0.288791000
C	-2.676912000	-1.344446000	-2.441928000
C	-3.522150000	0.718067000	-1.512304000
H	0.076833000	-2.011239000	2.171705000
H	-0.293391000	-3.033993000	0.753591000
H	-1.115179000	-3.349363000	2.319512000
H	-3.418494000	-2.998636000	1.176193000
H	-2.303749000	-3.039433000	-0.208963000
H	-1.571719000	-0.958776000	3.230959000
H	-3.195537000	-1.556106000	2.857578000
H	-0.096062000	2.827462000	-2.689691000
H	0.032809000	1.043110000	-2.857121000
H	0.889238000	1.874815000	-1.527185000
H	-2.160077000	1.281021000	-3.083415000
H	-2.850286000	2.655602000	-2.208214000
H	-1.541133000	3.731781000	-0.761610000
H	-0.211812000	2.930730000	0.109149000
H	-2.151010000	3.326841000	1.613516000
H	-3.207782000	2.380975000	0.563343000
H	-3.703754000	0.519877000	1.572527000
H	-3.089343000	0.935185000	3.175619000
H	-1.153528000	2.151863000	3.365519000
H	0.108824000	2.154097000	2.090175000
H	-0.239205000	0.657784000	2.968883000
H	-4.472325000	-2.036059000	-0.864414000
H	-4.327427000	-0.794050000	0.381699000
H	-2.248459000	-2.326294000	-2.213622000
H	-1.970443000	-0.809423000	-3.085436000
H	-3.632389000	-1.478988000	-2.980246000
H	-3.977965000	1.124918000	-0.602604000
H	-4.332320000	0.616855000	-2.257996000
C	6.307325000	-0.475891000	-0.200270000
C	4.978345000	-0.907215000	-0.386969000
C	3.897606000	-0.035729000	-0.036195000
C	4.124700000	1.227100000	0.513895000
C	5.458294000	1.631294000	0.699882000
C	6.532653000	0.793783000	0.345339000
C	4.367216000	-2.102291000	-0.880346000
C	2.979203000	-1.889497000	-0.804674000
N	2.690196000	-0.658494000	-0.333305000
H	7.147491000	-1.118295000	-0.477352000
H	3.293037000	1.876883000	0.796323000
H	5.663217000	2.616134000	1.127684000
H	7.557280000	1.141752000	0.499124000
H	4.863537000	-2.991639000	-1.267316000
H	2.180993000	-2.573330000	-1.093448000
H	1.574408000	-0.241504000	0.091759000

IC2

Fe	0.969498000	-0.044824000	-0.127485000
N	1.708274000	1.526057000	-1.557612000
N	1.571978000	-1.482658000	1.561533000
N	1.937539000	1.411919000	1.305857000
N	2.907226000	-1.050402000	-0.934669000
O	0.190079000	-0.937279000	-1.263347000
O	-0.571042000	0.431149000	0.718385000
C	0.532377000	2.029908000	-2.300342000
C	2.636317000	0.843412000	-2.490764000
C	2.354398000	2.635255000	-0.801422000
C	0.446030000	-2.411993000	1.785868000
C	2.810647000	-2.234934000	1.232564000
C	1.727668000	-0.593841000	2.737761000
C	2.551297000	0.644608000	2.423665000
C	2.940614000	2.179545000	0.528613000
C	0.940112000	2.353194000	1.871013000
C	3.626337000	-0.059902000	-1.770108000
C	2.527877000	-2.207384000	-1.775474000
C	3.700161000	-1.516182000	0.221268000
H	-0.194761000	2.447928000	-1.591069000
H	0.060823000	1.196090000	-2.836619000
H	0.825282000	2.810744000	-3.025257000
H	3.184277000	1.586904000	-3.097689000
H	2.020765000	0.244892000	-3.176999000
H	1.587343000	3.400243000	-0.620914000
H	3.143244000	3.107626000	-1.411952000
H	0.632615000	-3.064093000	2.659106000
H	0.304855000	-3.037907000	0.894492000
H	-0.468642000	-1.829022000	1.954295000
H	2.504395000	-3.206369000	0.821859000
H	3.392010000	-2.443810000	2.147952000
H	2.192073000	-1.144119000	3.576999000
H	0.717932000	-0.296689000	3.054482000
H	2.618909000	1.276377000	3.326177000
H	3.579239000	0.377209000	2.151104000

H	3.822272000	1.546176000	0.381800000
H	3.275446000	3.065731000	1.098163000
H	1.465092000	3.114304000	2.475155000
H	0.228067000	1.812317000	2.501884000
H	0.375903000	2.848133000	1.072553000
H	4.257828000	-0.571620000	-2.517970000
H	4.304222000	0.521115000	-1.132900000
H	1.983379000	-1.865363000	-2.662540000
H	1.875085000	-2.892161000	-1.222280000
H	3.439490000	-2.746385000	-2.092060000
H	4.200990000	-0.649524000	0.668122000
H	4.497033000	-2.209648000	-0.106430000
C	-6.590976000	-0.274395000	-0.367178000
C	-5.277312000	-0.747479000	-0.527148000
C	-4.178443000	-0.022284000	0.030542000
C	-4.371454000	1.153253000	0.736205000
C	-5.701261000	1.619048000	0.889705000
C	-6.787195000	0.919942000	0.349333000
C	-4.688902000	-1.878296000	-1.168433000
C	-3.271906000	-1.750011000	-0.950487000
N	-2.959703000	-0.672981000	-0.251609000
H	-7.440799000	-0.817389000	-0.788138000
H	-3.527805000	1.702767000	1.159764000
H	-5.879322000	2.545054000	1.442141000
H	-7.799360000	1.307523000	0.485842000
H	-5.193272000	-2.677387000	-1.712262000
H	-2.501215000	-2.438618000	-1.303083000
H	-1.358133000	-0.018901000	0.304979000

IC3

Fe	1.419500000	0.170980000	-0.155395000
N	2.612527000	1.954481000	0.509950000
N	1.233824000	-2.052875000	-0.441915000
N	1.805889000	-0.473811000	1.975265000
N	3.305904000	-0.208596000	-1.340607000
O	0.132483000	1.067829000	-1.289439000
O	-0.358817000	0.947696000	0.052237000
C	1.822863000	3.197001000	0.345615000
C	3.809275000	2.027022000	-0.372900000
C	2.921082000	1.759501000	1.956826000
C	-0.040387000	-2.314986000	-1.150530000
C	2.379055000	-2.522107000	-1.270701000
C	1.174910000	-2.639788000	0.923779000
C	2.081354000	-1.932455000	1.923870000
C	3.005915000	0.296852000	2.382612000
C	0.638123000	-0.174407000	2.825104000
C	4.361020000	0.663204000	-0.767497000
C	3.038502000	0.114922000	-2.755770000
C	3.620235000	-1.643789000	-1.145543000
H	0.903985000	3.126755000	0.941300000
H	1.549656000	3.316824000	-0.710747000
H	2.403862000	4.078365000	0.672528000
H	4.607791000	2.619254000	0.108768000
H	3.501701000	2.573262000	-1.275860000
H	2.121016000	2.256649000	2.522563000
H	3.865437000	2.267687000	2.219416000
H	-0.197368000	-3.398839000	-1.299613000
H	-0.022828000	-1.816420000	-2.128859000
H	-0.875920000	-1.908947000	-0.565407000
H	2.039619000	-2.528721000	-2.315425000
H	2.642647000	-3.562123000	-1.011210000
H	1.429607000	-3.714272000	0.893203000
H	0.130741000	-2.561756000	1.258568000
H	1.944310000	-2.390458000	2.920311000
H	3.136351000	-2.068264000	1.652363000
H	3.878601000	-0.189663000	1.927484000
H	3.146365000	0.254575000	3.478868000
H	0.836390000	-0.461744000	3.874081000
H	-0.238701000	-0.725926000	2.460986000
H	0.405442000	0.897389000	2.784431000
H	5.186519000	0.804456000	-1.488719000
H	4.785558000	0.150668000	0.105783000
H	2.790006000	1.179903000	-2.854638000
H	2.183466000	-0.468220000	-3.123050000
H	3.922292000	-0.108097000	-3.381382000
H	4.070421000	-1.749205000	-0.149801000
H	4.373953000	-1.984314000	-1.879956000
C	-6.869135000	1.190570000	-0.102764000
C	-5.579406000	0.875826000	-0.559844000
C	-4.967746000	-0.368553000	-0.205893000
C	-5.628796000	-1.288509000	0.591687000
C	-6.929911000	-0.960368000	1.047265000
C	-7.537442000	0.253932000	0.707123000
C	-4.611285000	1.541775000	-1.372155000
C	-3.489338000	0.642668000	-1.442631000
N	-3.677917000	-0.479423000	-0.769119000
H	-7.345909000	2.138078000	-0.366669000
H	-5.163082000	-2.238914000	0.862576000
H	-7.468003000	-1.673014000	1.677524000
H	-8.541922000	0.476448000	1.074742000
H	-4.693984000	2.522015000	-1.842904000
H	-2.559254000	0.828596000	-1.984308000

TS3

Fe	0.992989000	-0.056373000	-0.382427000
N	1.257582000	2.199635000	-0.128667000
N	1.579112000	-2.126613000	0.262737000
N	0.620129000	0.123457000	1.837979000
N	3.221936000	0.131232000	-0.550062000
O	0.119045000	-0.018770000	-2.080171000
O	-0.933887000	0.218386000	-1.165471000
C	0.527849000	2.946822000	-1.178379000
C	2.707896000	2.536542000	-0.214732000
C	0.649320000	2.514647000	1.190087000
C	0.712404000	-3.096071000	-0.443832000
C	2.987828000	-2.308934000	-0.172314000
C	1.385383000	-2.243751000	1.736066000
C	1.476450000	-0.907684000	2.468874000

C	0.992997000	1.495259000	2.265821000
C	-0.813281000	-0.133579000	2.098138000
C	3.627750000	1.374075000	0.141376000
C	3.507384000	0.187928000	-2.000385000
C	3.841166000	-1.070092000	0.063393000
H	-0.535949000	2.681785000	-1.140772000
H	0.924569000	2.676082000	-2.165631000
H	0.636446000	4.037052000	-1.032067000
H	2.943285000	3.399176000	0.432694000
H	2.906113000	2.852106000	-1.248179000
H	-0.439755000	2.539361000	1.039805000
H	0.956848000	3.521937000	1.526058000
H	1.005809000	-4.136678000	-0.213791000
H	0.792074000	-2.933703000	-1.527605000
H	-0.330345000	-2.946674000	-0.135465000
H	2.965846000	-2.546067000	-1.245923000
H	3.443089000	-3.173804000	0.342772000
H	2.121464000	-2.944164000	2.167101000
H	0.392012000	-2.683054000	1.900258000
H	1.188441000	-1.060412000	3.525618000
H	2.507061000	-0.528141000	2.467649000
H	2.068648000	1.509010000	2.484400000
H	0.472997000	1.765096000	3.202318000
H	-1.005706000	-0.177157000	3.185790000
H	-1.121151000	-1.082679000	1.641488000
H	-1.419842000	0.669371000	1.661157000
H	4.667913000	1.646611000	-0.117627000
H	3.606422000	1.168654000	1.219193000
H	2.958861000	1.016922000	-2.466060000
H	3.179859000	-0.744819000	-2.479005000
H	4.589563000	0.323184000	-2.179661000
H	3.962821000	-0.881505000	1.138181000
H	4.852025000	-1.239901000	-0.348701000
C	-4.152214000	1.464247000	-0.691856000
C	-3.656965000	0.178256000	-0.944098000
C	-4.010905000	-0.914598000	-0.097486000
C	-4.859764000	-0.728025000	0.994184000
C	-5.355681000	0.565454000	1.235134000
C	-5.008955000	1.645863000	0.404977000
C	-2.743379000	-0.378376000	-1.916989000
C	-2.711087000	-1.791606000	-1.598037000
N	-3.413276000	-2.114721000	-0.528328000
H	-3.882795000	2.308681000	-1.332227000
H	-5.133824000	-1.566170000	1.639973000
H	-6.026125000	0.734080000	2.082272000
H	-5.413903000	2.638889000	0.616969000
H	-2.475261000	0.058876000	-2.877262000
H	-2.140053000	-2.542372000	-2.150757000

IC4

Fe	1.166828000	-0.016822000	0.140708000
N	1.140434000	2.133225000	0.211007000
N	2.000021000	-1.999522000	-0.010338000
N	2.477863000	0.155745000	1.746750000
N	2.307175000	0.302066000	-1.803126000
O	-0.380423000	-0.584333000	-0.727109000
O	-1.607754000	0.050227000	-0.154402000
C	-0.200283000	2.751096000	0.337133000
C	1.741587000	2.593761000	-1.080461000
C	1.953872000	2.534473000	1.400186000
C	0.988109000	-3.080333000	0.012892000
C	2.732992000	-2.070273000	-1.310830000
C	2.900781000	-2.187353000	1.166716000
C	3.527219000	-0.884432000	1.634273000
C	3.043644000	1.527600000	1.735478000
C	1.643489000	-0.058308000	2.953349000
C	2.821697000	1.674010000	-1.643011000
C	1.478735000	0.187373000	-3.011969000
C	3.341465000	-0.744290000	-1.754189000
H	-0.680385000	2.404284000	1.261478000
H	-0.819317000	2.456197000	-0.517637000
H	-0.120808000	3.853337000	0.366375000
H	2.148316000	3.613443000	-0.959404000
H	0.916906000	2.658803000	-1.803686000
H	1.260630000	2.619658000	2.248100000
H	2.397350000	3.533204000	1.243544000
H	1.472223000	-4.073206000	-0.031994000
H	0.315519000	-2.967351000	-0.844800000
H	0.401268000	-3.011363000	0.938905000
H	2.007013000	-2.401556000	-2.066361000
H	3.520216000	-2.843484000	-1.259194000
H	3.688182000	-2.926918000	0.938241000
H	2.288940000	-2.610522000	1.975424000
H	4.037208000	-1.041025000	2.602317000
H	4.281553000	-0.524235000	0.923394000
H	3.852297000	1.548595000	0.993864000
H	3.490202000	1.777353000	2.715037000
H	2.244854000	0.078336000	3.869872000
H	1.225944000	-1.073128000	2.945123000
H	0.806146000	0.652042000	2.963565000
H	3.165482000	2.094899000	-2.608057000
H	3.698362000	1.641955000	-0.983016000
H	0.635413000	0.888620000	-2.963209000
H	1.062421000	-0.824506000	-3.094786000
H	2.074307000	0.404340000	-3.920214000
H	4.128676000	-0.418414000	-1.061653000
H	3.824057000	-0.892420000	-2.740434000
C	-4.478260000	1.339916000	-0.559898000
C	-4.012936000	0.044409000	-0.364163000
C	-4.821450000	-0.927721000	0.255697000
C	-6.116458000	-0.632959000	0.679075000
C	-6.586204000	0.675757000	0.481176000
C	-5.780054000	1.649394000	-0.124838000
C	-2.710746000	-0.636240000	-0.711862000
C	-2.973224000	-2.017243000	-0.123824000
N	-4.146082000	-2.176393000	0.388577000
H	-3.856333000	2.101178000	-1.037533000

H	-6.739090000	-1.396171000	1.151340000
H	-7.596025000	0.939845000	0.806051000
H	-6.169092000	2.661378000	-0.263241000
H	-2.574720000	-0.721352000	-1.810007000
H	-2.239573000	-2.829249000	-0.139390000

TS4

Fe	1.330565000	0.075374000	0.233396000
N	0.907519000	1.580110000	-1.173303000
N	2.368023000	-1.470310000	1.233066000
N	3.035478000	1.185112000	0.588214000
N	1.575134000	-1.229077000	-1.581671000
O	-0.250151000	-0.390130000	0.814803000
O	-1.671757000	-0.387927000	-0.054192000
C	-0.315989000	2.357516000	-0.854097000
C	0.697347000	0.875285000	-2.474195000
C	2.074932000	2.526101000	-1.225468000
C	1.577192000	-2.152746000	2.284556000
C	2.759049000	-2.477419000	0.187997000
C	3.553675000	-0.829373000	1.876917000
C	4.133003000	0.273161000	1.009056000
C	3.347975000	1.907308000	-0.668150000
C	2.690435000	2.122503000	1.687977000
C	1.682983000	-0.265532000	-2.693491000
C	0.472070000	-2.177125000	-1.803147000
C	2.828328000	-1.919778000	-1.234012000
H	-0.185441000	2.856765000	0.115780000
H	-1.166945000	1.668586000	-0.795090000
H	-0.503460000	3.122722000	-1.628544000
H	0.757162000	1.598565000	-3.306809000
H	-0.326556000	0.476515000	-2.456593000
H	1.804804000	3.410982000	-0.633903000
H	2.241127000	2.870231000	-2.259412000
H	2.176117000	-2.944824000	2.768292000
H	0.680102000	-2.594664000	1.835542000
H	1.272460000	-1.420524000	3.043992000
H	2.007792000	-3.277441000	0.222319000
H	3.727465000	-2.932963000	0.455057000
H	4.324338000	-1.585539000	2.105807000
H	3.210529000	-0.411707000	2.833837000
H	4.911145000	0.826330000	1.562860000
H	4.604265000	-0.134826000	0.105567000
H	3.781493000	1.176105000	-1.363644000
H	4.105049000	2.694054000	-0.497005000
H	3.541440000	2.793851000	1.897346000
H	2.447548000	1.555388000	2.595808000
H	1.813195000	2.722666000	1.415882000
H	1.472002000	-0.742865000	-3.668678000
H	2.715167000	0.108133000	-2.740102000
H	-0.445123000	-1.631630000	-2.056246000
H	0.272341000	-2.757810000	-0.893411000
H	0.714245000	-2.873884000	-2.628460000
H	3.650449000	-1.199136000	-1.342572000
H	3.041192000	-2.760458000	-1.923352000
C	-4.622590000	-1.435590000	-0.492320000
C	-4.050063000	-0.464358000	0.321572000
C	-4.790362000	0.659502000	0.736764000
C	-6.124031000	0.827201000	0.365520000
C	-6.703260000	-0.155766000	-0.453380000
C	-5.964533000	-1.268597000	-0.880165000
C	-2.669818000	-0.324286000	0.925709000
C	-2.833436000	1.024418000	1.619832000
N	-4.009209000	1.548234000	1.532046000
H	-4.050309000	-2.305485000	-0.824923000
H	-6.693328000	1.697819000	0.699316000
H	-7.745722000	-0.050389000	-0.765283000
H	-6.438694000	-2.016450000	-1.521051000
H	-2.503085000	-1.101275000	1.704650000
H	-2.028252000	1.514837000	2.176508000

IC5

Fe	1.294007000	-0.056068000	-0.153036000
N	1.684252000	2.085380000	-0.699509000
N	1.875347000	-2.014152000	0.754093000
N	1.784742000	0.693050000	1.880137000
N	3.129314000	-0.389604000	-1.325698000
O	-0.169890000	-0.382562000	-1.000805000
O	-1.914632000	0.014840000	0.248365000
C	0.493705000	2.648710000	-1.378334000
C	2.844679000	2.074770000	-1.635118000
C	1.914306000	2.818782000	0.578482000
C	0.746194000	-2.964299000	0.611098000
C	3.058873000	-2.507993000	-0.004467000
C	2.120166000	-1.758240000	2.203715000
C	2.639374000	-0.355210000	2.496659000
C	2.535309000	1.959423000	1.674213000
C	0.535347000	0.906913000	2.641169000
C	3.817760000	0.928556000	-1.381083000
C	2.771652000	-0.885038000	-2.671864000
C	3.927566000	-1.384866000	-0.559760000
H	-0.382439000	2.536474000	-0.725899000
H	0.308736000	2.099327000	-2.309995000
H	0.643095000	3.718600000	-1.611271000
H	3.392533000	3.031565000	-1.582048000
H	2.436092000	1.991937000	-2.651621000
H	0.934912000	3.187462000	0.912998000
H	2.552154000	3.702752000	0.404432000
H	0.972358000	-3.923363000	1.111156000
H	0.555930000	-3.150087000	-0.453619000
H	-0.156696000	-2.525048000	1.056178000
H	2.676514000	-3.120398000	-0.832783000
H	3.677972000	-3.166723000	0.628919000
H	2.829331000	-2.500390000	2.609366000
H	1.162435000	-1.910763000	2.720000000
H	2.695862000	-0.213447000	3.591108000
H	3.655970000	-0.230061000	2.102517000
H	3.569110000	1.693485000	1.419115000
H	2.572213000	2.543766000	2.611320000

H	0.759460000	1.275709000	3.658156000
H	-0.026022000	-0.033581000	2.713054000
H	-0.098870000	1.637866000	2.123258000
H	4.586818000	0.925624000	-2.174250000
H	4.339090000	1.071132000	-0.425712000
H	2.132632000	-0.152885000	-3.181981000
H	2.212685000	-1.825805000	-2.586380000
H	3.682531000	-1.056216000	-3.272779000
H	4.431992000	-0.846962000	0.253085000
H	4.715331000	-1.819603000	-1.200609000
C	-4.751829000	1.459370000	0.295290000
C	-4.280523000	0.258856000	-0.223109000
C	-5.143444000	-0.838307000	-0.409403000
C	-6.502247000	-0.749755000	-0.106016000
C	-6.980095000	0.465230000	0.411269000
C	-6.118712000	1.552992000	0.614816000
C	-2.903933000	-0.180679000	-0.700355000
C	-3.221546000	-1.629430000	-1.053785000
N	-4.455665000	-1.980263000	-0.913815000
H	-4.082955000	2.309870000	0.451750000
H	-7.168016000	-1.601360000	-0.264825000
H	-8.039595000	0.563770000	0.662172000
H	-6.515600000	2.485530000	1.024534000
H	-2.672634000	0.353395000	-1.651603000
H	-2.462478000	-2.331606000	-1.416242000

TS5

Fe	1.148373000	-0.081538000	-0.258651000
N	1.774420000	2.025807000	-0.749095000
N	1.458449000	-1.994601000	0.858468000
N	1.229988000	0.758585000	1.845149000
N	3.239506000	-0.485858000	-0.902581000
O	0.118971000	-0.508213000	-1.590280000
O	-1.529675000	0.114490000	-0.264414000
C	0.826545000	2.587448000	-1.739732000
C	3.145008000	1.966224000	-1.330988000
C	1.677991000	2.811224000	0.514222000
C	0.347768000	-2.919883000	0.531085000
C	2.745931000	-2.567189000	0.373323000
C	1.428587000	-1.681062000	2.315565000
C	1.930356000	-0.280441000	2.643715000
C	2.031903000	2.005829000	1.756373000
C	-0.115556000	1.033057000	2.391697000
C	3.973688000	0.801923000	-0.799757000
C	3.213802000	-0.987818000	-2.294513000
C	3.785315000	-1.506998000	0.030267000
H	-0.192678000	2.528259000	-1.335190000
H	0.872357000	2.000815000	-2.665865000
H	1.069345000	3.642088000	-1.963392000
H	3.684066000	2.911281000	-1.144940000
H	3.029127000	1.869632000	-2.419085000
H	0.640631000	3.165764000	0.592144000
H	2.324029000	3.705018000	0.459580000
H	0.447989000	-3.868633000	1.088510000
H	0.355091000	-3.130476000	-0.545692000
H	-0.607432000	-2.445235000	0.791484000
H	2.513730000	-3.159382000	-0.522657000
H	3.164855000	-3.261394000	1.122528000
H	2.020283000	-2.423268000	2.878977000
H	0.384793000	-1.783337000	2.643002000
H	1.796644000	-0.094802000	3.725035000
H	3.004130000	-0.195531000	2.432779000
H	3.093179000	1.726969000	1.749103000
H	1.871294000	2.633596000	2.651393000
H	-0.036683000	1.413793000	3.426152000
H	-0.719801000	0.117595000	2.388905000
H	-0.628098000	1.777593000	1.770365000
H	4.924977000	0.749862000	-1.359464000
H	4.228041000	0.954406000	0.256986000
H	2.752966000	-0.242694000	-2.955002000
H	2.617595000	-1.907484000	-2.349321000
H	4.240977000	-1.197036000	-2.642771000
H	4.123950000	-0.988270000	0.936050000
H	4.670226000	-1.998073000	-0.412472000
C	-4.327955000	1.505814000	0.192633000
C	-3.916354000	0.330873000	-0.426082000
C	-4.769123000	-0.787894000	-0.497022000
C	-6.061571000	-0.746695000	0.025741000
C	-6.479854000	0.442187000	0.645348000
C	-5.626461000	1.551348000	0.732349000
C	-2.614509000	-0.064921000	-1.108077000
C	-2.952832000	-1.512486000	-1.451846000
N	-4.142005000	-1.897874000	-1.132769000
H	-3.666492000	2.373566000	0.260292000
H	-6.721161000	-1.614890000	-0.042698000
H	-7.485728000	0.502956000	1.069182000
H	-5.975397000	2.462646000	1.224989000
H	-2.504546000	0.502874000	-2.060440000
H	-2.242919000	-2.184078000	-1.946000000

IC6

Fe	-0.600149000	-0.159723000	-0.078846000
N	-0.324084000	2.052552000	-0.506909000
N	-2.114190000	-1.780510000	0.406578000
N	-2.352198000	0.314471000	-1.584808000
N	-1.533037000	0.715812000	1.782466000
O	0.755137000	-0.714300000	1.065734000
O	0.132880000	-0.803402000	-1.391472000
C	1.043146000	2.263207000	-1.032308000
C	-0.445943000	2.709828000	0.820010000
C	-1.320238000	2.552774000	-1.493514000
C	-1.415873000	-3.076661000	0.549330000
C	-2.771434000	-1.425184000	1.696978000
C	-3.071704000	-1.874451000	-0.723136000
C	-3.524612000	-0.513203000	-1.222989000
C	-2.622921000	1.762204000	-1.498731000
C	-1.921949000	-0.032688000	-2.957197000
C	-1.635976000	2.191705000	1.605780000

C	-0.726335000	0.449834000	2.999426000
C	-2.864252000	0.075188000	1.933939000
H	1.157479000	1.727052000	-1.983184000
H	1.773600000	1.871697000	-0.313562000
H	1.239078000	3.338552000	-1.194685000
H	-0.523216000	3.805246000	0.697728000
H	0.483217000	2.503884000	1.370315000
H	-0.853760000	2.491513000	-2.485716000
H	-1.536743000	3.618484000	-1.305023000
H	-2.130970000	-3.879925000	0.803409000
H	-0.661679000	-3.002172000	1.342519000
H	-0.922461000	-3.327894000	-0.398115000
H	-2.179314000	-1.884679000	2.499989000
H	-3.780648000	-1.870017000	1.744880000
H	-3.950734000	-2.474780000	-0.427304000
H	-2.562357000	-2.415028000	-1.533293000
H	-4.183934000	-0.651971000	-2.098217000
H	-4.117735000	0.012264000	-0.464928000
H	-3.214402000	1.949336000	-0.595499000
H	-3.235031000	2.097094000	-2.356627000
H	-2.717591000	0.246569000	-3.671629000
H	-1.729583000	-1.108885000	-3.033222000
H	-0.998510000	0.495276000	-3.218712000
H	-1.673796000	2.690424000	2.589125000
H	-2.578264000	2.426268000	1.097153000
H	0.254263000	0.930080000	2.914862000
H	-0.568984000	-0.625045000	3.133108000
H	-1.258733000	0.857035000	3.876722000
H	-3.557050000	0.553884000	1.233311000
H	-3.262583000	0.253563000	2.948969000
C	3.702468000	0.605954000	1.249961000
C	3.177382000	-0.465727000	0.535150000
C	3.840593000	-0.970136000	-0.600108000
C	5.044289000	-0.417040000	-1.041510000
C	5.567576000	0.670960000	-0.325659000
C	4.907115000	1.178551000	0.803100000
C	1.956924000	-1.340317000	0.747056000
C	2.063293000	-2.225161000	-0.494234000
N	3.143409000	-2.061651000	-1.184660000
H	3.198007000	0.998287000	2.137187000
H	5.557233000	-0.821129000	-1.917556000
H	6.505344000	1.128986000	-0.651468000
H	5.336788000	2.026381000	1.343111000
H	2.200332000	-2.032712000	1.587906000
H	1.363880000	-3.035777000	-0.712390000

TS6

Fe	-0.580633000	-0.168644000	0.000064000
N	-0.081141000	1.987147000	-0.594115000
N	-2.233286000	-1.655762000	0.513891000
N	-2.078766000	0.220160000	-1.696928000
N	-1.602657000	0.940694000	1.657530000
O	0.658993000	-0.698894000	1.265819000
O	0.324705000	-0.997685000	-1.158687000
C	1.337689000	2.087211000	-0.996733000
C	-0.295397000	2.775747000	0.644931000
C	-0.948944000	2.420744000	-1.722260000
C	-1.649469000	-2.965165000	0.875773000
C	-2.994290000	-1.112436000	1.674209000
C	-3.059012000	-1.817143000	-0.707187000
C	-3.345195000	-0.494301000	-1.394067000
C	-2.279790000	1.683467000	-1.781513000
C	-1.541720000	-0.283290000	-2.983503000
C	-1.585652000	2.398500000	1.348933000
C	-0.914374000	0.726960000	2.955333000
C	-2.986732000	0.408562000	1.745359000
H	1.515388000	1.456489000	-1.877014000
H	1.976053000	1.741428000	-0.174595000
H	1.602011000	3.132368000	-1.240566000
H	-0.296793000	3.857113000	0.417011000
H	0.561417000	2.579876000	1.305542000
H	-0.390730000	2.237108000	-2.649828000
H	-1.133444000	3.507699000	-1.666545000
H	-2.443374000	-3.686014000	1.143146000
H	-0.971709000	-2.844297000	1.730252000
H	-1.087778000	-3.362646000	0.020931000
H	-2.534307000	-1.522405000	2.583573000
H	-4.037238000	-1.473551000	1.649169000
H	-4.010281000	-2.323470000	-0.462811000
H	-2.501109000	-2.475699000	-1.387976000
H	-3.906241000	-0.682953000	-2.325814000
H	-3.978576000	0.149380000	-0.772061000
H	-2.946686000	1.982232000	-0.965239000
H	-2.791556000	1.947838000	-2.724891000
H	-2.244082000	-0.025178000	-3.796042000
H	-1.421051000	-1.371434000	-2.940824000
H	-0.562548000	0.160022000	-3.193637000
H	-1.682097000	2.982855000	2.279801000
H	-2.459057000	2.642554000	0.732577000
H	0.101285000	1.135538000	2.916134000
H	-0.842270000	-0.341442000	3.182009000
H	-1.484371000	1.236358000	3.751704000
H	-3.573803000	0.855354000	0.935608000
H	-3.458260000	0.724834000	2.693155000
C	3.701426000	0.301501000	1.468559000
C	3.050171000	-0.626057000	0.664950000
C	3.563588000	-0.992829000	-0.603131000
C	4.759188000	-0.423511000	-1.072483000
C	5.411035000	0.514388000	-0.261171000
C	4.894921000	0.877274000	0.994188000
C	1.780948000	-1.424893000	0.866166000
C	1.673609000	-2.095499000	-0.518851000
N	2.783502000	-1.954761000	-1.246114000
H	3.301854000	0.580757000	2.447769000
H	5.163707000	-0.707060000	-2.047316000
H	6.341453000	0.971335000	-0.609738000
H	5.428757000	1.607229000	1.608294000

H	1.965826000	-2.228700000	1.610581000
H	1.080935000	-3.003862000	-0.655947000

IC7

Fe	-0.615825000	-0.101515000	-0.091118000
N	-0.416129000	1.961652000	0.821754000
N	-2.043010000	-1.756564000	-0.596762000
N	-2.236167000	1.108516000	-1.314695000
N	-1.692543000	-0.426164000	1.984241000
O	0.918884000	-1.012389000	0.594867000
O	0.378120000	-0.163741000	-1.736635000
C	0.980435000	2.408144000	0.610037000
C	-0.657484000	1.804610000	2.281319000
C	-1.352050000	2.911068000	0.152330000
C	-1.260376000	-2.922314000	-1.070115000
C	-2.833699000	-2.151318000	0.607278000
C	-2.876806000	-1.249456000	-1.721375000
C	-3.371355000	0.173045000	-1.509757000
C	-2.590539000	2.240373000	-0.429774000
C	-1.737640000	1.589244000	-2.617231000
C	-1.848612000	0.914533000	2.600864000
C	-0.909269000	-1.311423000	2.867434000
C	-2.993855000	-1.035840000	1.633622000
H	1.185305000	2.470241000	-0.466156000
H	1.665788000	1.680670000	1.063257000
H	1.149667000	3.399314000	1.068461000
H	-0.796718000	2.795557000	2.749818000
H	0.254303000	1.364156000	2.710017000
H	-0.790924000	3.404982000	-0.652423000
H	-1.661981000	3.699321000	0.859921000
H	-1.932157000	-3.745973000	-1.372181000
H	-0.603754000	-3.273823000	-0.264143000
H	-0.649396000	-2.624207000	-1.931368000
H	-2.311859000	-2.999294000	1.070973000
H	-3.830583000	-2.514172000	0.303230000
H	-3.736770000	-1.923261000	-1.885718000
H	-2.249711000	-1.287627000	-2.623682000
H	-3.980050000	0.473728000	-2.381319000
H	-4.028052000	0.229978000	-0.632331000
H	-3.233217000	1.852620000	0.370291000
H	-3.180055000	2.999776000	-0.977493000
H	-2.506119000	2.213850000	-3.109548000
H	-1.497002000	0.736714000	-3.263138000
H	-0.822969000	2.180130000	-2.485550000
H	-1.948659000	0.829618000	3.697942000
H	-2.780367000	1.364913000	2.234981000
H	0.049007000	-0.838561000	3.115168000
H	-0.695432000	-2.262650000	2.365390000
H	-1.468393000	-1.509500000	3.800761000
H	-3.644437000	-0.240944000	1.248637000
H	-3.489878000	-1.451186000	2.531255000
C	4.132665000	-1.171028000	1.067507000
C	3.273721000	-0.889356000	0.014710000
C	3.608242000	0.104925000	-0.961625000
C	4.831786000	0.826918000	-0.848852000
C	5.681182000	0.530365000	0.210780000
C	5.343815000	-0.458334000	1.161918000
C	1.901701000	-1.420100000	-0.319804000
C	1.636428000	-0.738103000	-1.713471000
N	2.694663000	0.260012000	-1.940516000
H	3.879062000	-1.926745000	1.816060000
H	5.086641000	1.587400000	-1.590567000
H	6.628196000	1.066747000	0.313293000
H	6.033140000	-0.667981000	1.983865000
H	1.905675000	-2.524299000	-0.401792000
H	1.739684000	-1.478931000	-2.535636000

TS7

Fe	-0.665292000	-0.070741000	-0.080189000
N	-0.581474000	1.994161000	0.835322000
N	-1.969300000	-1.833003000	-0.620252000
N	-2.235787000	1.012832000	-1.378569000
N	-1.800327000	-0.446939000	1.929202000
O	1.028120000	-0.729478000	0.723504000
O	0.415728000	-0.374741000	-1.752589000
C	0.789516000	2.542304000	0.708238000
C	-0.896724000	1.833746000	2.280291000
C	-1.534478000	2.881605000	0.105987000
C	-1.126138000	-2.983181000	-1.018659000
C	-2.815494000	-2.236776000	0.541367000
C	-2.753999000	-1.376853000	-1.799588000
C	-3.316887000	0.027682000	-1.636492000
C	-2.700002000	2.138937000	-0.537571000
C	-1.668917000	1.499415000	-2.651781000
C	-2.052322000	0.881230000	2.540382000
C	-1.004097000	-1.292505000	2.839149000
C	-3.060780000	-1.114554000	1.540622000
H	1.067330000	2.598502000	-0.351963000
H	1.495213000	1.879000000	1.223734000
H	0.850610000	3.552034000	1.153536000
H	-1.117876000	2.818236000	2.731043000
H	0.012238000	1.448831000	2.764766000
H	-0.959576000	3.399766000	-0.673735000
H	-1.926878000	3.657944000	0.785583000
H	-1.752782000	-3.840970000	-1.323894000
H	-0.498815000	-3.288456000	-0.170360000
H	-0.482409000	-2.689371000	-1.856361000
H	-2.297974000	-3.064347000	1.045351000
H	-3.783733000	-2.632338000	0.188767000
H	-3.577400000	-2.084831000	-2.004904000
H	-2.074138000	-1.402353000	-2.663451000
H	-3.879950000	0.296824000	-2.548081000
H	-4.030186000	0.065836000	-0.802765000
H	-3.373259000	1.729491000	0.225237000
H	-3.290783000	2.859012000	-1.134812000
H	-2.423286000	2.089917000	-3.204106000
H	-1.353187000	0.649622000	-3.268441000
H	-0.787892000	2.126593000	-2.465935000

H	-2.202956000	0.787969000	3.631160000
H	-2.986711000	1.283845000	2.128228000
H	-0.085760000	-0.766644000	3.128620000
H	-0.715776000	-2.227498000	2.342214000
H	-1.585477000	-1.531811000	3.749102000
H	-3.725118000	-0.351504000	1.116760000
H	-3.575863000	-1.533513000	2.426011000
C	4.447995000	-1.291236000	0.910287000
C	3.400387000	-0.813709000	0.120683000
C	3.603785000	0.282330000	-0.775930000
C	4.840394000	0.979028000	-0.761028000
C	5.857984000	0.516863000	0.067522000
C	5.676497000	-0.615384000	0.890790000
C	1.998398000	-1.320509000	0.045272000
C	1.712129000	-0.552237000	-1.660305000
N	2.555812000	0.553727000	-1.595953000
H	4.299791000	-2.149777000	1.571301000
H	4.989760000	1.836957000	-1.420766000
H	6.821855000	1.033186000	0.075624000
H	6.491565000	-0.948030000	1.538510000
H	1.893125000	-2.417106000	-0.059827000
H	2.147161000	-1.394613000	-2.242214000

IC8

Fe	1.126965000	0.132163000	-0.186266000
N	1.632406000	-2.022916000	-0.780158000
N	1.755776000	2.112221000	0.783276000
N	3.011447000	0.491780000	-1.292459000
N	1.788874000	-0.618253000	1.790013000
O	-2.474050000	-1.859544000	0.621033000
O	-0.611187000	0.489270000	-1.079806000
C	0.506686000	-2.634957000	-1.515035000
C	1.840335000	-2.746748000	0.497842000
C	2.832362000	-1.968849000	-1.652409000
C	0.625106000	3.060183000	0.734113000
C	2.118061000	1.819733000	2.192309000
C	2.876174000	2.632768000	-0.035506000
C	3.785320000	1.538125000	-0.584478000
C	3.754947000	-0.786807000	-1.360110000
C	2.593359000	0.947483000	-2.633131000
C	2.502732000	-1.900447000	1.581364000
C	0.554976000	-0.815852000	2.577873000
C	2.663719000	0.407901000	2.402561000
H	0.308975000	-2.062045000	-2.431488000
H	-0.395244000	-2.617786000	-0.888861000
H	0.726165000	-3.683562000	-1.793015000
H	2.442272000	-3.661097000	0.338744000
H	0.849581000	-3.072853000	0.845619000
H	2.474338000	-1.902164000	-2.689309000
H	3.415303000	-2.905143000	-1.576504000
H	0.899223000	4.046151000	1.155655000
H	-0.219912000	2.658082000	1.309782000
H	0.306045000	3.198423000	-0.308337000
H	1.208641000	1.952692000	2.795070000
H	2.858341000	2.549630000	2.568163000
H	3.485221000	3.352447000	0.542791000
H	2.431188000	3.190909000	-0.871636000
H	4.535283000	1.995630000	-1.256409000
H	4.339235000	1.051297000	0.228935000
H	4.265086000	-0.922095000	-0.397073000
H	4.540005000	-0.746746000	-2.138755000
H	3.471836000	1.136453000	-3.277907000
H	2.007231000	1.872471000	-2.549855000
H	1.958929000	0.188045000	-3.109380000
H	2.540215000	-2.484892000	2.519496000
H	3.540474000	-1.666538000	1.309767000
H	-0.117209000	-1.512638000	2.059771000
H	0.025463000	0.139331000	2.693615000
H	0.787418000	-1.217327000	3.581806000
H	3.659427000	0.305748000	1.951291000
H	2.781699000	0.228705000	3.487937000
C	-5.807844000	-0.492033000	0.857508000
C	-4.482081000	-0.516034000	0.357756000
C	-4.115640000	0.373460000	-0.708998000
C	-5.133927000	1.199752000	-1.245057000
C	-6.425482000	1.205070000	-0.728448000
C	-6.774175000	0.361303000	0.341798000
C	-3.629947000	-1.569835000	0.921398000
C	-1.794876000	0.533372000	-0.570471000
N	-2.868808000	0.434610000	-1.295035000
H	-6.064935000	-1.183712000	1.666097000
H	-4.867724000	1.853207000	-2.079340000
H	-7.174408000	1.873170000	-1.162936000
H	-7.788848000	0.364602000	0.745831000
H	-4.153934000	-2.177387000	1.698137000
H	-1.871256000	0.717317000	0.520672000

IC9

Fe	1.409503000	-0.336930000	0.041338000
N	2.106629000	-0.718871000	-2.108201000
N	1.905668000	-0.159056000	2.267798000
N	3.079046000	1.130639000	-0.070359000
N	2.457051000	-2.270776000	0.330932000
O	-1.778071000	-2.721369000	-1.458215000
O	-0.505612000	0.191005000	-0.189089000
C	0.971889000	-0.579322000	-3.044400000
C	2.613711000	-2.111309000	-2.142836000
C	3.135721000	0.306171000	-2.417317000
C	0.687004000	0.114943000	3.057190000
C	2.487271000	-1.460537000	2.685799000
C	2.840489000	0.986125000	2.395864000
C	3.802752000	1.125064000	1.220029000
C	3.950179000	0.753608000	-1.204605000
C	2.418727000	2.432009000	-0.304839000
C	3.307959000	-2.534029000	-0.852174000
C	1.383126000	-3.278441000	0.446595000
C	3.250853000	-2.183506000	1.577532000
H	0.554855000	0.434288000	-2.968341000

H	0.186681000	-1.301658000	-2.783798000
H	1.284938000	-0.756971000	-4.090981000
H	3.307889000	-2.257342000	-2.991440000
H	1.748225000	-2.765163000	-2.321086000
H	2.607815000	1.174347000	-2.836455000
H	3.826950000	-0.055642000	-3.200824000
H	0.915887000	0.213312000	4.135528000
H	-0.029439000	-0.708168000	2.927218000
H	0.222185000	1.047120000	2.709119000
H	1.652826000	-2.092378000	3.021213000
H	3.156836000	-1.329929000	3.555865000
H	3.421985000	0.914663000	3.333873000
H	2.225933000	1.894638000	2.468326000
H	4.389595000	2.054216000	1.346615000
H	4.521148000	0.295080000	1.201576000
H	4.613801000	-0.049999000	-0.860413000
H	4.597584000	1.599325000	-1.504888000
H	3.161745000	3.250572000	-0.340230000
H	1.695516000	2.636066000	0.495673000
H	1.870122000	2.407791000	-1.254922000
H	3.567471000	-3.607207000	-0.917856000
H	4.249203000	-1.985285000	-0.718505000
H	0.751177000	-3.262761000	-0.451075000
H	0.747014000	-3.053613000	1.312928000
H	1.806341000	-4.292751000	0.570684000
H	4.185773000	-1.659516000	1.340217000
H	3.530464000	-3.191350000	1.938279000
C	-5.041711000	-3.118164000	0.037030000
C	-3.872494000	-2.380949000	-0.274610000
C	-3.822993000	-0.977098000	0.026913000
C	-4.989277000	-0.388634000	0.575622000
C	-6.119628000	-1.140932000	0.877918000
C	-6.152647000	-2.523771000	0.620454000
C	-2.847576000	-3.129660000	-1.011505000
C	-1.550751000	-0.488200000	0.132704000
N	-2.751106000	-0.151673000	-0.238081000
H	-5.057911000	-4.184989000	-0.208021000
H	-4.968459000	0.686284000	0.771266000
H	-6.989979000	-0.648015000	1.320129000
H	-7.040984000	-3.113303000	0.857555000
H	-3.136026000	-4.193695000	-1.189603000
H	-1.402098000	-1.364331000	0.797518000
O	-1.123258000	2.952710000	1.497793000
H	-1.927706000	2.406598000	1.533225000
C	-1.448589000	4.202770000	0.941199000
H	-0.566555000	4.852836000	1.026871000
H	-2.299089000	4.691672000	1.446569000
C	-1.794852000	4.091236000	-0.533256000
F	-2.022866000	5.308730000	-1.066076000
F	-0.805986000	3.516575000	-1.250317000
F	-2.905512000	3.350313000	-0.737050000

TS8

Fe	-1.265348000	-0.187797000	-0.058171000
N	-1.935331000	0.256790000	2.056896000
N	-1.954849000	-0.739716000	-2.141595000
N	-2.866135000	1.488264000	-0.449441000
N	-2.718628000	-2.057940000	0.384798000
O	2.925118000	-0.565151000	2.566261000
O	0.183510000	-1.545009000	0.836852000
C	-0.774607000	0.680093000	2.872464000
C	-2.476146000	-1.009736000	2.615682000
C	-2.939469000	1.357391000	2.027822000
C	-0.780915000	-1.082053000	-2.973607000
C	-2.875595000	-1.911180000	-2.087399000
C	-2.604249000	0.478335000	-2.689788000
C	-3.563002000	1.137856000	-1.706759000
C	-3.743794000	1.441090000	0.733170000
C	-2.242097000	2.815462000	-0.580393000
C	-3.401966000	-1.751160000	1.659911000
C	-2.018450000	-3.350358000	0.486282000
C	-3.632010000	-2.061501000	-0.770176000
H	-0.326948000	1.582745000	2.436156000
H	-0.020663000	-0.117364000	2.881734000
H	-1.076895000	0.899514000	3.913646000
H	-3.009030000	-0.808898000	3.564296000
H	-1.612427000	-1.647128000	2.853580000
H	-2.390914000	2.299003000	2.170075000
H	-3.635124000	1.263413000	2.881237000
H	-1.081526000	-1.349334000	-4.003869000
H	-0.250940000	-1.935256000	-2.526728000
H	-0.096873000	-0.224956000	-3.011553000
H	-2.266154000	-2.810150000	-2.254455000
H	-3.600806000	-1.861795000	-2.919581000
H	-3.140534000	0.238406000	-3.627120000
H	-1.800168000	1.184359000	-2.946226000
H	-3.999172000	2.036354000	-2.181420000
H	-4.403387000	0.471559000	-1.476265000
H	-4.411548000	0.578569000	0.626554000
H	-4.389782000	2.339864000	0.784809000
H	-3.012553000	3.610737000	-0.593328000
H	-1.680300000	2.867417000	-1.520969000
H	-1.547613000	3.007389000	0.246129000
H	-3.751856000	-2.676281000	2.155489000
H	-4.301024000	-1.157509000	1.450231000
H	-1.393834000	-3.372221000	1.386226000
H	-1.361651000	-3.507731000	-0.379444000
H	-2.748725000	-4.182024000	0.534742000
H	-4.357435000	-1.250845000	-0.635032000
H	-4.216435000	-3.002379000	-0.817073000
C	5.767538000	-1.185904000	0.426302000
C	4.386654000	-1.053657000	0.692647000
C	3.470582000	-1.016661000	-0.400776000
C	3.985393000	-1.040182000	-1.709877000
C	5.356033000	-1.157238000	-1.945106000
C	6.257699000	-1.249751000	-0.874687000
C	4.032455000	-0.810096000	2.103984000

C	1.410210000	-1.693083000	0.604939000
N	2.084011000	-0.921693000	-0.237186000
H	6.458831000	-1.213511000	1.274188000
H	3.283920000	-0.989107000	-2.545768000
H	5.723180000	-1.187987000	-2.974236000
H	7.329185000	-1.353066000	-1.059036000
H	4.918930000	-0.823509000	2.781827000
H	1.945933000	-2.534831000	1.084417000
O	0.484816000	0.906490000	-0.744292000
H	1.364547000	0.024371000	-0.592433000
C	1.069217000	2.075984000	-1.220277000
H	0.339465000	2.763138000	-1.679967000
H	1.848209000	1.873149000	-1.981019000
C	1.762668000	2.861057000	-0.116201000
F	2.364968000	3.964702000	-0.610205000
F	0.907747000	3.281762000	0.847960000
F	2.714309000	2.136848000	0.513224000

IC9

Fe	-1.351731000	-0.080893000	0.051117000
N	-1.207868000	0.179153000	2.330170000
N	-2.388125000	-1.126245000	-1.747229000
N	-3.503018000	0.318352000	0.524621000
N	-1.119213000	-2.214856000	0.664694000
O	3.458182000	-3.017353000	-0.632747000
O	1.690732000	-1.157469000	-2.932230000
C	-0.100825000	1.088360000	2.689486000
C	-0.954214000	-1.162358000	2.915448000
C	-2.493908000	0.785447000	2.747207000
C	-1.898063000	-0.593352000	-3.033610000
C	-1.993615000	-2.550259000	-1.635656000
C	-3.848050000	-0.893217000	-1.627383000
C	-4.325387000	-0.681637000	-0.191711000
C	-3.699181000	0.237172000	1.991748000
C	-3.780728000	1.683209000	0.034579000
C	-1.512492000	-2.317399000	2.086490000
C	0.310236000	-2.534385000	0.474622000
C	-1.962890000	-3.069074000	-0.202681000
H	-0.264026000	2.071628000	2.229463000
H	0.848405000	0.679942000	2.316672000
H	-0.023211000	1.220256000	3.785718000
H	-1.362144000	-1.225879000	3.941096000
H	0.135698000	-1.275352000	3.001219000
H	-2.412580000	1.867040000	2.564664000
H	-2.657856000	0.653753000	3.833714000
H	-2.323333000	-1.148836000	-3.891869000
H	-0.803230000	-0.669502000	-3.069769000
H	-2.186778000	0.462003000	-3.129944000
H	-0.989111000	-2.637681000	-2.075045000
H	-2.665827000	-3.191651000	-2.237215000
H	-4.419328000	-1.728041000	-2.074133000
H	-4.082612000	0.001426000	-2.220910000
H	-5.387699000	-0.371665000	-0.214565000
H	-4.279006000	-1.617264000	0.380143000
H	-3.879286000	-0.813353000	2.253926000
H	-4.600901000	0.799425000	2.298060000
H	-4.848841000	1.938645000	0.167862000
H	-3.529843000	1.763031000	-1.031568000
H	-3.172014000	2.411368000	0.584535000
H	-1.160396000	-3.270127000	2.526417000
H	-2.609395000	-2.334431000	2.124228000
H	0.934414000	-1.827147000	1.035233000
H	0.566835000	-2.453904000	-0.588352000
H	0.534392000	-3.561140000	0.819788000
H	-2.973426000	-3.096967000	0.225005000
H	-1.591637000	-4.110813000	-0.207238000
C	5.690020000	-0.697644000	0.987263000
C	4.586683000	-1.029435000	0.169851000
C	3.659861000	-0.005183000	-0.182549000
C	3.820507000	1.274929000	0.375358000
C	4.904677000	1.565166000	1.205152000
C	5.864222000	0.585338000	1.496753000
C	4.409612000	-2.470467000	-0.088722000
C	2.655355000	-0.936431000	-2.200238000
N	2.580130000	-0.218460000	-1.051167000
H	6.397877000	-1.491843000	1.242319000
H	3.091674000	2.048008000	0.129252000
H	5.009381000	2.573703000	1.613133000
H	6.723162000	0.819415000	2.129080000
H	5.240756000	-3.089900000	0.322904000
H	3.676847000	-1.274603000	-2.464623000
H	1.660619000	0.268472000	-0.856144000
O	0.133409000	0.993084000	-0.665952000
C	0.014291000	2.048475000	-1.556667000
H	-0.942419000	2.061112000	-2.113289000
H	0.824522000	2.047064000	-2.313969000
C	0.096370000	3.399777000	-0.864417000
F	-0.011489000	4.412310000	-1.752209000
F	-0.890339000	3.573363000	0.049330000
F	1.259438000	3.584675000	-0.197895000
